# Phase II study of ipilimumab and nivolumab in leptomeningeal carcinomatosis

**DOI:** 10.1038/s41467-021-25859-y

**Published:** 2021-10-12

**Authors:** Priscilla K. Brastianos, Matthew R. Strickland, Eudocia Quant Lee, Nancy Wang, Justine V. Cohen, Ugonma Chukwueke, Deborah Anne Forst, April Eichler, Beth Overmoyer, Nancy U. Lin, Wendy Y. Chen, Aditya Bardia, Dejan Juric, Ibiayi Dagogo-Jack, Michael D. White, Jorg Dietrich, Naema Nayyar, Albert E. Kim, Christopher Alvarez-Breckenridge, Maura Mahar, Joana L. Mora, Brian V. Nahed, Pamela S. Jones, Helen A. Shih, Elizabeth R. Gerstner, Anita Giobbie-Hurder, Scott L. Carter, Kevin Oh, Daniel P. Cahill, Ryan J. Sullivan

**Affiliations:** 1 Massachusetts General Hospital, 55 Fruit Street, Boston, MA 02114 USA; 2 Dana-Farber Cancer Institute, 450 Brookline Avenue, Boston, MA 02215 USA

**Keywords:** Immunotherapy, Cancer therapy, Metastasis, Tumour immunology

## Abstract

Leptomeningeal disease (LMD) is a common complication from solid tumor malignancies with a poor prognosis and limited treatment options. We present a single arm Phase II study of 18 patients with LMD receiving combined ipilimumab and nivolumab until progression or unacceptable toxicity (NCT02939300). The primary end point is overall survival at 3 months (OS3). Secondary end points include toxicity, cumulative time-to-progression at 3 months, and progression-free survival. A Simon two-stage design is used to compare a null hypothesis OS3 of 18% against an alternative of 44%. Median follow up based on patients still alive is 8.0 months (range: 0.5 to 15.9 months). The study has met its primary endpoint as 8 of 18 (OS3 0.44; 90% CI: 0.24 to 0.66) patients are alive at three months. One third of patients have experienced one (or more) grade-3 or higher adverse events. Two patients have discontinued protocol treatment due to unacceptable toxicity (hepatitis and colitis, respectively). The most frequent adverse events include fatigue (N = 7), nausea (N = 6), fever (N = 6), anorexia (N = 6) and rash (N = 6). Combined ipilimumab and nivolumab has an acceptable safety profile and demonstrates promising activity in LMD patients. Larger, multicenter clinical trials are needed to validate these results.

## Abstract (web summary)

Leptomeningeal metastases from solid tumors are a rare complication with a very poor prognosis. Here the authors report the efficacy and safety of combined ipilimumab and nivolumab in patients with leptomeningeal carcinomatosis.

## Introduction

Leptomeningeal disease (LMD), also known as leptomeningeal carcinomatosis or carcinomatosis meningitis, is a phenomenon characterized by metastatic dissemination of cancer cells to the leptomeninges. LMD has been reported to occur in up to 10% of solid tumors and in 5–15% of hematological malignancies^1–3^. Incidence is increasing owing to improved systemic therapies resulting in longer patient survival as well as development of more sensitive detection methods. Primary solid tumor histologies most frequently associated with development of LMD include those from breast, lung, and skin (melanoma) origins. Although LMD is often identified concurrently with parenchymal disease, it can also occur in the absence of parenchymal brain metastases. Presenting symptoms can include headache, nausea, vomiting, neurological deficits, and gait instability. Unfortunately, the prognosis for patients that develop LMD is poor ranging from 3 to 7 weeks on average^1,4–13^.

Although several treatment modalities are available to patients with LMD, there is significant practice variation due to a paucity of data from prospective clinical trials^13^. Multimodality therapy is often employed for palliation of symptoms and optimizing quality of life as opposed to prolonging survival. Radiation therapy approaches include stereotactic radiosurgery (SRS) typically for large, bulky, and symptom-causing lesions, whole-brain radiation therapy (WBRT) for those with poor performance status who are unlikely to tolerate other treatment modalities and craniospinal irradiation (CSI). In clinical trials, systemic and intrathecal therapies (IT) have demonstrated mixed results for survival benefit, likely owing to multiple factors such as heterogeneous primary tumor biology, clinical trial enrollment bias toward more fit patients, and low accrual. Toxicities from these traditional treatment approaches remain a major concern and can include leukoencephalopathy, subacute encephalopathy, acute cerebellar syndrome, aseptic meningitis (up to 43% of patients), and infectious meningitis (8–24% of patients receiving IT therapy)^3,14,15^. Patients with LMD have an urgent need for therapies that can provide a meaningful survival benefit with an acceptable toxicity profile.

Recently, there have been remarkable advances in the treatment of parenchymal brain metastases with immune checkpoint inhibitors such as ipilimumab—a cytotoxic T-lymphocyte antigen 4 (CTLA4) inhibitor—and nivolumab—an anti-programmed death 1 agent (PD1) inhibitor—demonstrating promising intracranial activity in patients who have traditionally been excluded from clinical trials^16–20^. While increasing participation of patients with brain metastases in modern investigational efforts is encouraging, patients with leptomeningeal carcinomatosis represent an understudied population and could be uniquely poised to derive improved outcomes in the era of immunotherapy. Here, we present a phase II single-arm clinical trial in patients with LMD from diverse primary solid tumor histologies showing promising intracranial activity of combined ipilimumab and nivolumab associated with improved overall survival compared to historical controls, and an acceptable safety profile.

## Results

### Patients

Eighteen patients were enrolled at the Massachusetts General Hospital and the Dana-Farber Cancer Institute from February 2018 to April 2019. The majority of patients were female (61%) and white (77.8%) with a median age of 54 years (range 36–70; Table 1). Primary diagnosis of breast cancer occurred in 44% (*N* = 8) of patients; 50% of these primary tumors were estrogen receptor (ER) positive, 25% were progesterone receptor (PR) positive, 25% were human epidermal growth factor receptor 2 (HER2) positive and 25% were triple negative. Primary diagnoses of anaplastic astrocytoma, esophageal adenocarcinoma, ependymoma, gastroesophageal junction adenocarcinoma, glioblastoma, non-small cell lung cancer (NSCLC), ovarian carcinoma, and small cell lung cancer occurred, respectively, in one patient each. Two patients had a primary diagnosis of melanoma. No patients in the enrolled cohort had a known *BRAF* or *EGFR* mutation or an *ALK* rearrangement in their primary tumor. Patient co-morbidities are summarized in Supplementary Table 1. The median time between primary cancer diagnosis and study enrollment was 29.5 months (range: 3–276 months). At the time of primary tumor diagnosis, 22% (*N* = 4) of patients had brain metastases, one patient had cerebrospinal fluid (CSF) involvement and 11% (*N* = 2) had known extracranial disease. At the time of trial enrollment, LMD was confirmed in 72.2% of patients (*N* = 13) by positive CSF cytology and 27.7% (*N* = 5) by imaging), and 72.2% (*N* = 13) of patients had two or more parenchymal brain metastases. Sixty-one percent (*N* = 11) had known extracranial disease with most common sites being lung (*N* = 6) and lymph nodes (*N* = 3). Patients were heavily pre-treated having received an average of 3.1 prior systemic therapies (range: 1–8). No patients received concurrent radiation, surgery nor intrathecal therapy while on protocol. Thirty-three percent (*N* = 6) of patients received prior radiation therapy for their primary tumor while 50% (*N* = 9) of patients received prior radiation for metastatic disease (Table 2). Sixty-one percent (*N* = 11) received CNS-directed radiation; 44% (*N* = 8) completed their course prior to enrollment and 17% (*N* = 3) completed their course after trial participation. Among patients who received CNS-directed radiation, 45% (*N* = 5) received whole-brain radiation therapy (WBRT), 18% (*N* = 2) received stereotactic radiosurgery, 18% (*N* = 2) received intensity-modulated radiation therapy (IMRT), and 18% (*N* = 2) received craniospinal irradiation. Seventy-two percent (*N* = 13) received prior surgery for their primary tumor. Fifty percent (*N* = 9) reported additional operations for metastatic disease (median number of operations of 2 (average 2.7, range: 1–8)). Twenty-eight percent (*N* = 5) of patients received CNS-directed surgery for disease prior to trial enrollment; one of these patients received an additional operation after trial participation. Eighty-three percent (*N* = 15) of patients had a ventriculoperitoneal shunt and/or ommaya reservoir placed but no concurrent intrathecal therapies were administered during trial enrollment. Thirty-nine percent (*N* = 7) of patients reported additional malignancies which were not considered to be active or progressing including Hodgkin’s lymphoma (*N* = 1), melanoma (*N* = 1), carcinoma of cervix (*N* = 1), neuro-endocrine carcinoid tumor of the appendiceal orifice (*N* = 1), esophageal carcinoma (*N* = 1), and breast cancer (*N* = 1). One patient had a compound nevus with mild melanocytic dysplasia (*N* = 1). One patient had a stable lung nodule whose biopsy status was unknown. Seventy-eight percent (*N* = 14) of patients received corticosteroids at any point while enrolled on trial. Of these 14 patients, 50% (*N* = 7) were receiving corticosteroids at baseline during trial enrollment while the other 50% (*N* = 7) initiated corticosteroids throughout the course of trial participation. Of those 7 patients already receiving corticosteroids at baseline, 5 later required increased dosing, one later tolerated decreased dosing, and one of these patients required no corticosteroid dose change over the course of the trial.

**Table 1:** Patient demographics and baseline disease characteristics.

Sex, no. (%)	*N*
Female	11 (61.1)
Male	7 (38.9)
Median age, years (range)	54 (36–70)
Race	
White	14 (77.8)
Asian	2 (11.1)
Other	2 (11.1)
ECOG performance status, no. (%)	
0	4 (22.2)
1	10 (55.6)
2	4 (22.2)
Primary tumor diagnosis, no. (%)	
Breast Cancer	8 (44.4)
ER + PR− HER2−	2 (11.1)
ER + PR + HER2−	2 (11.1)
ER + PR + HER2+	0 (0)
ER− PR− HER2+	2 (11.1)
Triple negative	2 (11.1)
Melanoma	2 (11.1)
Anaplastic astrocytoma	1 (5.6)
Esophageal adenocarcinoma	1 (5.6)
Ependymoma	1 (5.6)
GE junction adenocarcinoma	1 (5.6)
Glioblastoma	1 (5.6)
Lung adenocarcinoma	1 (5.6)
Ovarian	1 (5.6)
Small cell lung carcinoma	1 (5.6)
Brain metastases at the time of LMD diagnosis, no. (%)	
Yes	13 (72.2)
No	5 (16.7)
Extracranial disease at the time of LMD diagnosis, no. (%)	
Yes	11 (61.1)
No	5 (27.8)
Unknown	2 (11.1)
Bone	2 (11.1)
Liver	2 (11.1)
Lung	6 (33.3)
Lymph nodes	3 (16.7)
Skin	1 (5.6)
Breast	2 (11.1)
Spleen	1 (5.6)
Additional malignancy (non-active)	7 (38.9)

*ECOG* Eastern Cooperative Oncology Group, *ER* estrogen receptor, *PR* progesterone receptor, *LMD* leptomeningeal dissemination of cancer.

**Table 2:** Patient demographics: other therapy.

	No. (%)
Radiation for primary disease	6 (33.3)
Radiation for metastatic disease	9 (50.0)
Radiation for CNS disease	11 (61.1)
Before trial participation	8 (44.4)
After trial participation	3 (16.7)
Surgery for primary disease	13 (72.2)
Surgery for metastatic disease	9 (50.0)
Surgery for CNS disease	5 (27.8)
Before trial participation	5 (27.8)
After trial participation	1 (5.6)
Systemic therapy for primary disease	11 (61)
Systemic therapy for metastatic disease	9 (50.0)
*Patients receiving corticosteroids, no. (%)*	14 (77.8)

Trial participation defined as time enrolled on study.

*CNS* central nervous system.

### Efficacy

Patients received a total of 40 doses of ipilimumab with a median of 2 doses per patient (range: 1–4), and a total of 51 doses of nivolumab with a median of 2 doses per patient (range: 1–10). All regimens were delivered intravenously. Eight patients (44%) were alive at 3 months after enrollment (OS3 proportion = 0.44; 90% CI: 0.24–0.66, Table 3, Supplemental Table 3). Per the pre-specified criteria, the requirement for a successful primary endpoint under the study design was six or more patients alive at 3 months. Therefore, the study met its primary endpoint.

**Table 3:** Efficacy.

	*N*	%
*Rate of overall survival at 3 months*		
Alive at 3 months	8	44
Not alive at 3 months	10	56
*Best response iRANO*		
Complete response	1	5.6
Stable disease	7	38.9
Progressive disease	4	22.2
Not evaluable^a^	6	33.3
*Best response RECIST*^b^		
Partial response	1	5.6
Stable disease	3	16.7
Progressive disease	3	16.7
Not evaluable^a^	11	61.1

^a^Includes cases where there is no evaluable disease at baseline or follow-up exams or where restaging scans not obtained.

^b^Responses as assessed by immune-related response criteria (irRC) were consistent with RECIST criteria.

**Fig. 1:**
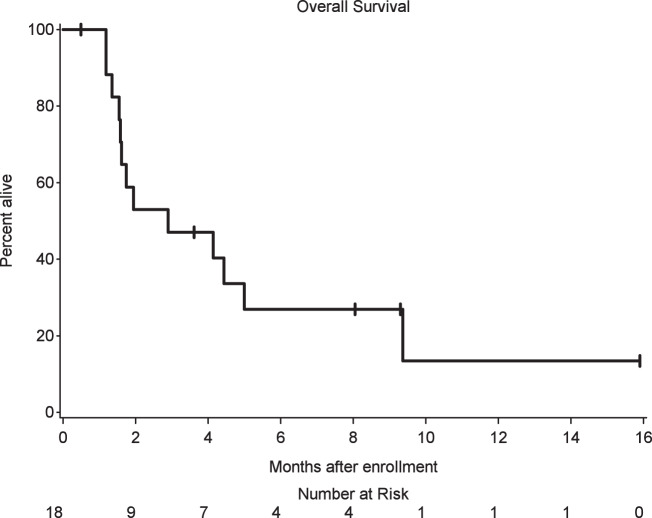
Overall survival in 18 patients with LMD from solid tumors. Median overall survival was 2.9 months (90% CI: 1.6–5.0 months).

There were 13 deaths out of 18 patients. Median survival was 2.9 months (90% CI: 1.6–5.0 months) (Fig. 1). One patient withdrew consent and stopped participation in the study. For CNS disease including LMD response, responses were assessed every 6–8 weeks with MRI using iRANO criteria, with one patient showing a complete response, and 7 of the 18 patients showing stable disease in the CNS. Post-treatment initiation CSF cytology was only available for 4 patients and so interpretation of LMD response rate by cytology was limited. Of available data for patients with progressive disease by iRANO criteria, cumulative incidence of time to progression for intracranial sites at 3 months was 45% (90% CI: 25–80%) and the intracranial progression-free survival (PFS) median was 1.93 months (90% CI: 1.28–2.66 months). For extracranial disease, responses were assessed every 6–8 weeks with CT chest/abdomen/pelvis using RECIST criteria. Of the 7 patients with evaluable disease at baseline and restaging scans obtained, 1 patient had a partial response, 3 demonstrated stable disease, and 3 demonstrated progressive disease. Of available data for evaluable patients with progressive disease by RECIST criteria, the cumulative incidence of time to progression for extracranial sites at 3 months was 33% (90% CI: 8–62%) and the progression-free survival for extracranial disease was a median of 1.94 months (90% CI: 1.35–4.44 months). Responses by immune-related response criteria (irRC) were consistent with RECIST data by comparison. Comparing concordance for intra- and extracranial best responses, there were 6 patients with evaluable data. One patient demonstrated concordant treatment responses, 3 patients demonstrated concordant stable disease, one patient demonstrated concordant progression of disease, and finally, one patient demonstrated discordant responses (intracranial disease remained stable while extracranial disease progressed).

We evaluated cell counts, glucose, and protein from CSF samples collected as part of clinical care before and during treatment with ipilimumab and nivolumab (Supplementary Figure 1, Supplementary Table 2). From available data, there was no relationship between protein level nor absolute lymphocyte count in the CSF and overall survival. Data from all 18 patients for CSF glucose values suggested that a higher pre-treatment value (>57.5) was associated with longer OS of 5.0 months (90% CI: 1.6-not reached) versus 1.7 months (90% CI: 1.3–4.4) but this was not statistically significant (*p* = 0.09). Results from additional correlative studies, including single-cell sequencing data from the CSF, show evidence of an immune response (increased CD8+ T cell outgrowth and increased IFN-γ signaling) in the CSF with systemically administered PD1-axis and CTLA4 blockade^21^.

### Adverse events

Sixteen of 18 patients had one or more adverse events (AEs) that were considered to be possibly treatment-related (Table 4, Supplementary Table 4). Six patients had one or more grade-3 or higher AEs that were at least possibly related to treatment (Supplementary Table 5). The observed percentage is 33% (90% exact CI: 16–55%). Two patients discontinued protocol treatment due to unacceptable toxicity (hepatitis and colitis, respectively). The most frequently occurring AEs deemed possibly related to protocol therapy were fatigue (*N* = 7), nausea (*N* = 6), fever (*N* = 6), anorexia (*N* = 6), and rash (*N* = 6). There were six fatal events, none of which were deemed to be related to treatment.

**Table 4:** Treatment-related events occurring in at least 15% of patients, all grade 3 or 4 toxicities and immune-related adverse events.

Toxicity description CTCAE v4.0		Grade 1 or 2	Grade 3	Grade 4
		*N* (%)	*N* (%)	*N* (%)
Gastrointestinal disorders	Abdominal pain or discomfort	4 (22%)	–	–
	Constipation	4 (22%)	–	–
	Diarrhea	3 (17%)	1 (6%)	–
	Nausea	6 (33%)	–	–
	Vomiting	5 (28%)	–	–
Constitutional	Fatigue	7 (39%)	–	–
	Fever	6 (33%)	–	–
	Weight loss	4 (22%)	–	–
	Anorexia	6 (33%)	–	–
Hepatobiliary disorders infections	Hepatitis	–	1 (6%)	–
	Alanine aminotransferase increased^a^	3 (17%)	2 (11%)	–
	Aspartate aminotransferase increased^a^	4 (22%)	2 (11%)	–
	Alkaline phosphatase increased^a^	2 (11%)	–	–
	Amylase increased	–	1 (6%)	–
	Lipase increased	–	–	1 (6%)
	Aspiration pneumonia	–	1 (6%)	–
	Sepsis	–	1 (6%)	–
Renal	Acute kidney injury	–	1 (6%)	–
	Hypokalemia	–	1 (6%)	–
Musculoskeletal	Back pain	4 (22%)	–	–
Nervous system	Encephalopathy	–	1 (6%)	–
	Headache	4 (22%)	–	–
Skin disorders	Pruritus	4 (22%)	–	–
	Rash	6 (33%)	–	–
Vascular disorders	Hypotension	–	1 (6%)	–

^a^Immune-related adverse events.

## Discussion

LMD represents a significant and increasingly frequent challenge in the care for cancer patients. Current treatment options rarely improve clinical outcomes and there is an urgent need for effective novel therapies. Unfortunately, the developmental pipeline for investigational agents is hindered by several factors owing to the late manifestation of LMD such as heavy pre-treatment history, widely metastatic cancer often with acquired resistance, poor performance status, and dismal prognosis at the time of diagnosis measured in weeks. A synergy of these factors drives a vicious cycle of patients in dire need of new treatment options who are typically excluded from clinical trial participation. In the era of immunotherapy, there is renewed hope for advances in the treatment of LMD. In this Phase 2 study, we demonstrate that combination treatment with CTLA4 and PD1 inhibitors was safe and associated with a 3-months OS of 44% in a heavily pre-treated population of patients with LMD.

We designed our trial with several LMD-specific considerations in mind given the difficulty in assessing treatment response clinically, radiographically, and pathologically^13^. Our patient cohort was heavily pretreated with an average of 3.1 prior systemic therapies and 78% of patients had a performance status of 1 or higher. Using a Simon two-stage design, we selected a stringent overall survival threshold at 3 months as a primary endpoint—nearly double the poor median survival of 3–7 weeks observed in this population in historical series^1,4,6,8–11,13^. We deliberately avoided surrogate endpoints and instead relied on modern iRANO criteria^22^ to more objectively assess responses in the CNS and showed that 39% of patients had stable disease in the CNS as their best response. Our cohort included a dominant fraction of patients with breast cancer—a histology with a high propensity to develop LMD—and other primary tumor histologies such as glioblastoma and other primary brain tumors which have previously been felt to demonstrate limited response to immune checkpoint inhibition (ICI)^23,24^. In fact, of the 8 patients alive at 3 months, 5 had breast cancer as their primary tumor diagnosis while two had LMD arising from primary brain tumors (glioblastoma and ependymoma).

To our knowledge, this is one of the first prospective studies focused on an LMD-specific patient population with mixed primary tumor histologies. Although there has been exciting progress in recent years studying the efficacy of ICI for the treatment of brain metastases^16–20,25^, patients with LMD have generally been excluded. For example, seminal work by Tawbi et al. showed impressive intracranial activity, concordant extracranial response, and prolonged overall survival with combined ipilimumab/nivolumab in patients with brain metastases from melanoma^19^. However, patients with LMD were specifically excluded in their trial. Long et al. similarly studied combined ipilimumab/nivolumab and single-agent nivolumab in patients with brain metastases from melanoma and included 4 patients with LMD treated with nivolumab single-agent—none of whom demonstrated a clinical intracranial response. One retrospective series found data suggesting some patients with LMD from NSCLC will derive clinical benefit from ICI that can be durable^20^. Thus, our study provides prospective evidence that ICI can lead to intra- and extracranial activity in this understudied patient population similar to results seen in patients with brain metastases.

While historically the CNS has been considered an immune privileged or “sanctuary” site, we hypothesize that peripheral immune cells can gain entry to the CSF compartment analogous to patients with brain metastases who may have blood–brain barrier disruption. Work by Taggart et al. showed evidence that CD8+ T cells and NK cells were required for observed intracranial activity by combined CTLA4 and PD1 blockade in murine models of metastatic melanoma with brain metastases^26^. Further, Taggart et al. demonstrated that intracranial activity resulted at least in part from peripheral expansion of CD8+ T cells with subsequent trafficking to intracranial tumor sites^26^. Current mechanistic understanding of immune cell entry into the CNS (and various compartments such as brain parenchyma or CSF) remains limited, however. Further study in larger patient populations with biomarker correlative work is needed in order to fully explore this hypothesis.

Our data demonstrate a safety and toxicity profile comparable to previous reports using ICI with no unexpected events, deaths nor excess neurological toxicities observed^17–19^. Given median follow-up based on patients alive of 8.0 months (range: 0.5–15.9 months) it is possible that some immune-related adverse events (irAEs) may have developed that were not captured in the timeframe of our data analysis. Given that 78% of patients received corticosteroids while on trial—and that two-thirds of all patients either initiated or increased dosing of corticosteroids throughout participation in the trial—it is possible that this common corticosteroid requirement may have mitigated some toxicities and confounded a low observed frequency of adverse events. In the same way, the frequent corticosteroid usage could have potentially diminished clinical activity of ICI. While this possibility remains speculative, it is nevertheless encouraging that the study still met its primary endpoint. All four patients who did not receive corticosteroids at any point while on trial were among those alive at 3 months, and thus contributed to the positive primary endpoint. This association remains descriptive, however, due to such a small sample size. Importantly, the majority of our patient cohort had a performance status of 1 or higher suggesting ICI should not be withheld for deconditioned patients. We interpret these data to support combined ICI as a safe and tolerable therapeutic option for patients with LMD warranting further study in larger patient populations.

Our study had several limitations. Small sample size and high attrition due to declining clinical condition limits the generalizability of our findings. In addition, our patient cohort had a heterogeneous group of primary tumor histologies with relatively low representation of melanoma and NSCLC—two cancer types frequently associated with the development of LMD. Discovery of predictive biomarkers that will guide appropriate patient selection is paramount. Dosing regimen and schedule for ipilimumab and nivolumab varied depending on primary tumor histology. While this design was per manufacturer’s guidelines, varied dosing/administration schedule could have contributed to variability in treatment responses. It is also possible that a naturally more indolent illness course for some of these select patients could account for longer overall survival compared to historical precedent. Finally, the increasingly common integration of ICIs into frontline therapy for metastatic melanoma and lung cancer may limit the feasibility of considering these agents for treating LMD which typically manifests as a late and sequentially distal event.

In conclusion, our results show that treatment of patients with LMD with combined ipilimumab and nivolumab can demonstrate both intra- and extracranial clinical activity leading to extended overall survival compared to historical controls. The safety profile of this combined regimen was similar to previous reports and did not demonstrate excessive neurological or autoimmune toxicity. Further study of ICI in patients with LMD is needed in larger patient populations and in combination with other therapeutic modalities such as radiation therapy or chemotherapy.

## Methods

### Study design and patients

#### Study oversight

The study (Clinicaltrials.gov identifier NCT02939300) was designed by the principal investigators and conducted in accordance with the provision of the Declaration of Helsinki and Good Clinical Practice guidelines. The Dana-Farber Harvard Cancer Center (DF/HCC) Institutional review board committee approved the protocol. Funding was provided by Bristol Myers Squibb and Massachusetts General Hospital. The full study protocol is available as Supplementary Note 1 in the Supplementary Information file.

#### Patients

Eligible patients were >18 years of age, had histologically confirmed disease from any solid tumor with an ECOG PS ≤ 2, normal organ and marrow function, life expectancy >3 weeks, were on a stable dose of dexamethasone of 2 mg or less for 7 days prior to initiation of treatment, and had leptomeningeal meningitis as defined by positive cytology or biopsy, or had imaging findings consistent with leptomeningeal meningitis if cerebrospinal fluid cytology was negative. There were no specific radiographic criteria for eligibility. Patients who had received prior CNS-directed treatment, including prior treatment for leptomeningeal meningitis, were eligible. Patients were excluded if they had a diagnosis of immunodeficiency or active autoimmune disease, had a known history of active non-infectious pneumonitis, received prior treatment with a PD-1 or PD-L1 inhibitor or had received systemic immunosuppressive treatments aside from corticosteroids within 3 months of study drug. Written informed consent was obtained for all participants. Eighteen patients were enrolled at the Massachusetts General Hospital and the Dana-Farber Cancer Institute from February 2018 to April 2019.

#### Study design, treatment, and endpoints

This was a single-arm Phase II study of ipilimumab and nivolumab in patients with LMD from solid tumors. The following dosing regimens specific to primary tumor histology were administered intravenously as directed by the manufacturer’s (Bristol Meyers Squibb) guidelines: patients with melanoma were treated with nivolumab (1 mg/kg) combined with ipilimumab (3 mg/kg) every 3 weeks for a total of 4 doses followed by nivolumab monotherapy (480 mg) every 4 weeks until progression or unacceptable toxicity, patients with non-small cell lung cancer were treated with nivolumab (3 mg/kg) every 2 weeks combined with ipilimumab (1 mg/kg) every 6 weeks, patients with small cell lung cancer and breast cancer were treated with nivolumab (1 mg/kg) combined with ipilimumab (3 mg/kg) every 3 weeks for a total of 4 doses followed by nivolumab (240 mg) every 2 weeks and patients with other solid tumors (not listed above) were treated with nivolumab (3 mg/kg) combined with ipilimumab (1 mg/kg) every 3 weeks for a total of 4 doses followed by nivolumab (480 mg) every 4 weeks. No intrathecal therapies were permitted while patients were enrolled in the trial. A brain MRI and CT chest/abdomen/pelvis were obtained every 6 weeks for restaging in all patients except for patients with melanoma, breast cancer, small cell lung cancer, bladder cancer, and other solid tumors in the maintenance phase of treatment who received a brain MRI and CT chest/abdomen/pelvis every 8 weeks. The primary endpoint was the rate of overall survival at 3 months (OS3). Any patient whose vital status at 3 months could not be determined was counted as having died. Secondary objectives included toxicity, cumulative time-to-progression at 3 months, and progression-free survival for both intracranial sites using immune Response Assessment in Neurooncology (iRANO)^22^ as well as extracranial sites using Response Evaluation Criteria in Solid Tumors (RECIST) 1.1. All radiographic images were reviewed centrally by radiologists through the Tumor Imaging Metrics Core (TIMC) using these prespecified imaging criteria. Clinical data were collected using InForm software (version 6.2). All participants were re-evaluated for response every 6 weeks. In addition to a baseline scan, confirmatory scans were obtained 3 weeks following initial documentation of objective response.

### Statistical analysis

With a historical median overall survival of ~3–7 weeks in this patient population (as determined from previous reports in the literature^1,4–13^ and our institutional database), a Simon two-stage design was used to compare a null hypothesis that OS3 would be 18% against an alternative of 44%. Nine patients were to be enrolled in the first stage. If 0 or 1 patients are alive at 3 months, the trial would stop early. If 2 or more patients are alive at 3 months, an additional 9 patients would be enrolled. If at least 6 patients among the total of 18 patients are alive at 3 months, then the treatment would be considered promising in the cohort. This design had a type-I error of 8% (target 10%) and power of 86% (target 85%). If the null hypothesis were true, then the probability would be 0.50 of stopping at the end of the first stage of the Simon design. On October 23, 2018, a third patient was alive at 3 months; therefore, enrollment continued without pause into the second stage.

The primary endpoint, OS3, and CNS and extracranial response rates are summarized with 90% exact binomial confidence intervals. Toxicities that were new or worsening relative to baseline are summarized according to the worst grade occurring for each patient. The distribution of overall survival is presented using the method of Kaplan–Meier with 90% confidence intervals estimated using log(−log) methods. Statistical analyses were conducted using SAS 9.4 (SAS Institute Inc., Cary, NC, USA).

### Reporting summary

Further information on research design is available in the Nature Research Reporting Summary linked to this article.

## Supplementary information

Supplementary Information

Reporting Summary

## Data Availability

The full study protocol is available as Supplementary Note 1 in the Supplementary Information file. Any requests for additional clinical data will be reviewed by the Dana-Farber/Harvard Cancer Center (DF/HCC) Institutional Review Board (IRB). Patient-related data not included in the paper were generated as part of a clinical trial and are subject to patient confidentiality. Any data and materials (e.g., tissue samples or imaging data) that can be shared will need approval from the DF/HCC IRB and a Material Transfer Agreement in place. All data shared will be de-identified. Any requests for clinical data should be addressed to the corresponding author Priscilla K. Brastianos (pbrastianos@mgh.harvard.edu).

## References

[1] Grossman SA, Krabak MJ (1999). Leptomeningeal carcinomatosis. Cancer Treat. Rev..

[2] Le Rhun E, Preusser M, van den Bent M, Andratschke N, Weller M (2019). How we treat patients with leptomeningeal metastases. ESMO Open.

[3] Beauchesne P (2010). Intrathecal chemotherapy for treatment of leptomeningeal dissemination of metastatic tumours. Lancet Oncol..

[4] Boyle R, Thomas M, Adams JH (1980). Diffuse involvement of the leptomeninges by tumour—a clinical and pathological study of 63 cases. Postgrad. Med. J..

[5] Chamberlain MC (2012). Neurotoxicity of intra-CSF liposomal cytarabine (DepoCyt) administered for the treatment of leptomeningeal metastases: a retrospective case series. J. Neurooncol..

[6] Chuang TY, Yu CJ, Shih JY, Yang PC, Kuo SH (2008). Cytologically proven meningeal carcinomatosis in patients with lung cancer: clinical observation of 34 cases. J. Formos. Med. Assoc..

[7] Clamon G, Doebbeling B (1987). Meningeal carcinomatosis from breast cancer: spinal cord vs. brain involvement. Breast Cancer Res. Treat..

[8] Grossman SA (1993). Randomized prospective comparison of intraventricular methotrexate and thiotepa in patients with previously untreated neoplastic meningitis. Eastern Cooperative Oncology Group. J. Clin. Oncol..

[9] Lara-Medina F (2012). Clinical features and prognostic factors in patients with carcinomatous meningitis secondary to breast cancer. Breast J..

[10] Little JR, Dale AJ, Okazaki H (1974). Meningeal carcinomatosis. Clinical manifestations. Arch. Neurol..

[11] Ongerboer de Visser BW (1983). Intraventricular methotrexate therapy of leptomeningeal metastasis from breast carcinoma. Neurology.

[12] Rosen ST (1982). Carcinomatous leptomeningitis in small cell lung cancer: a clinicopathologic review of the National Cancer Institute experience. Medicine.

[13] Wang N, Bertalan MS, Brastianos PK (2018). Leptomeningeal metastasis from systemic cancer: Review and update on management. Cancer.

[14] Chamberlain MC (2010). Leptomeningeal metastasis. Semin Neurol..

[15] Chamberlain MC, Kormanik PA, Barba D (1997). Complications associated with intraventricular chemotherapy in patients with leptomeningeal metastases. J. Neurosurg..

[16] Goldberg SB (2016). Pembrolizumab for patients with melanoma or non-small-cell lung cancer and untreated brain metastases: early analysis of a non-randomised, open-label, phase 2 trial. Lancet Oncol..

[17] Long GV (2018). Combination nivolumab and ipilimumab or nivolumab alone in melanoma brain metastases: a multicentre randomised phase 2 study. Lancet Oncol..

[18] Margolin K (2012). Ipilimumab in patients with melanoma and brain metastases: an open-label, phase 2 trial. Lancet Oncol..

[19] Tawbi HA (2018). Combined nivolumab and ipilimumab in melanoma metastatic to the brain. N. Engl. J. Med..

[20] Hendriks LEL (2019). Survival of patients with non-small cell lung cancer having leptomeningeal metastases treated with immune checkpoint inhibitors. Eur. J. Cancer.

[21] Prakadan, S. M. et al. Genomic and transcriptomic correlates of immunotherapy response within the tumor microenvironment of leptomeningeal metastases. *Nat. Commun.*10.1038/s41467-021-25860-5 (2021). 10.1038/s41467-021-25860-5 PMC8511044 34642316

[22] Okada H (2015). Immunotherapy response assessment in neuro-oncology: a report of the RANO working group. Lancet Oncol..

[23] Dirix LY (2018). Avelumab, an anti-PD-L1 antibody, in patients with locally advanced or metastatic breast cancer: a phase 1b JAVELIN Solid Tumor study. Breast Cancer Res. Treat..

[24] Lim M, Xia Y, Bettegowda C, Weller M (2018). Current state of immunotherapy for glioblastoma. Nat. Rev. Clin. Oncol..

[25] Duchnowska R (2016). Immune response in breast cancer brain metastases and their microenvironment: the role of the PD-1/PD-L axis. Breast Cancer Res..

[26] Taggart D (2018). Anti-PD-1/anti-CTLA-4 efficacy in melanoma brain metastases depends on extracranial disease and augmentation of CD8(+) T cell trafficking. Proc. Natl Acad. Sci. USA.

