# Supplementary material for: Phase II study of ipilimumab and nivolumab in leptomeningeal carcinomatosis
Source: Nat Commun. 2021 Oct 12;12:5954. doi: 10.1038/s41467-021-25859-y (PMC8511104; doi:10.1038/s41467-021-25859-y)
Supplement: Supplementary file 1 — Supplementary Information [file 41467_2021_25859_MOESM1_ESM.pdf]

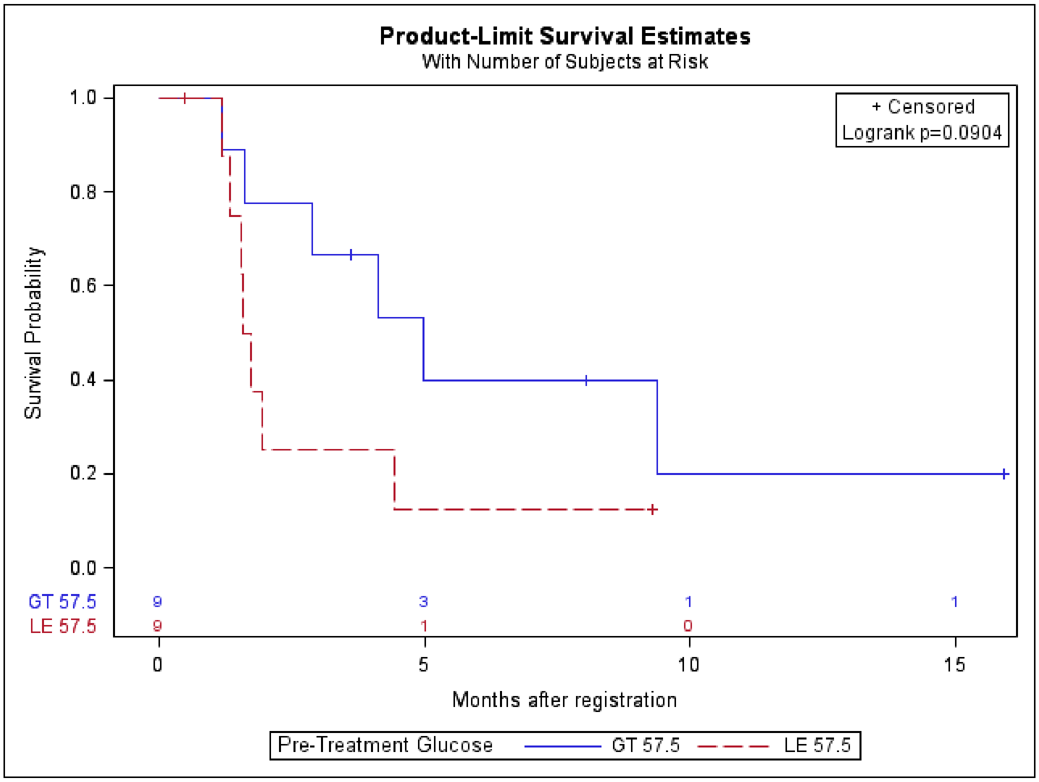

Supplementary Figure 1. Kaplan-Meier plot showing overall survival stratified by high (N=9) versus low (N=9) CSF glucose pre-treatment values (divided at median). Glucose low: 1.7 months (90% CI: 1.3 – 4.4). Glucose high: 5.0 months (90% CI: 1.6 – not reached). Log-rank statistical test: p=0.0904. GT, greater than; LE, less than or equal to.

Supplementary Table 1. Patient co-morbidities

Medical Co-morbidities	
Type I diabetes mellitus	1 (5.6)
Hypothyroidism	1 (5.6)
Allergies	1 (5.6)
Hypertension	4 (22.2)
Coronary artery disease	2 (11.1)
History of myocardial infarction	1 (5.6)
Ischemic cardiomyopathy	1 (5.6)
Left ventricular thrombus	1 (5.6)
Atrial enlargement	1 (5.6)
Cardiac arrhythmia	3 (16.7)

Supplementary Table 2. Fold-change (FC) in CSF glucose, protein and absolute lymphocytes in pre- versus post-treatment with ipilimumab and nivolumab using the Wilcoxon signed-rank test.

	N	*Mean*	*Std*	*Min*	*Median*	*Max*	*p-Value*
FC glucose	5	0.95	0.17	0.77	0.86	1.16	p=0.63
FC protein	5	1.74	1.93	0.11	1.03	5.00	p=0.81
FC absolute lymphocytes	2	8.28	11.26	0.32	8.28	16.24	p=0.99

Supplementary Table 3. Clinicopathologic features for patients alive at 3 months

Case number	OS (months)	Primary Tumor	Positive CSF cytology at time of LMD diagnosis?	Best Response to Therapy	Duration of trial participation (days)	ECOG performance status at enrollment	# lines of prior systemic therapy	Prior intracranial radiation	Prior CNS surgery	Baseline corticosteroid use at enrollment	Change in corticosteroid dose during trial participation	# of lines of systemic treatments post-trial participation	Post-trial intracranial radiation	Post-trial CNS-directed surgery
2	9.4	Melanoma	no	PD	22	1	3	none	no	yes	increase	3	WBRT	none
3	15.9	Ependymoma	yes	SD	61	1	1	IMRT, SRS	yes	yes	no	none	none	yes
7	5.0	ER/PR neg, Her2+ Breast Cancer	yes	SD	65	1	3	WBRT	yes	no	no	1	none	none
9	4.4	ER/PR neg, Her2+ Breast Cancer	yes	SD	78	1	3	WBRT	no	yes	initiation	none	none	none
11	9.3	ER+, PR neg, Her2 neg Breast Cancer	yes	unknown	27	1	1	none	none	no	no	1	WBRT	none
12	3.6	Glioblastoma	no	SD	109	1	1	IMRT	yes	no	no	1	none	none
14	8.0	Triple Negative Breast Cancer	yes	CR	154	2	3*	none	no	no	no	0#	none	none
17	4.1	ER/PR+, Her2 neg Breast Cancer	yes	SD	116	1	9*	none	no	yes	decrease	none	none	none

OS=overall survival, CSF=cerebrospinal fluid, LMD=leptomeningeal carcinomatosis/disease, ECOG=eastern cooperative oncology group, CNS=central nervous sytem, PD=progressive disease, SD=stable disease, CR=complete response, WBRT=whole brain radiation therapy, IMRT=intensity-modulated radiation therapy, SRS=stereotactic radiosurgery, ER=estrogen receptor, PR=progesterone receptor, HER2=human epidermal growth factor receptor-2

*including intrathecal therapy

#patient continued maintenance nivolumab until data cut off: May 2020

Supplementary Table 4. All adverse events at least possibly related to treatment

		Toxicity Grade CTCAE v4.0			
		01 - Mild	*02 - Moderate*	*03 - Severe*	*04 - Life-Threatening*
		N	*N*	*N*	*N*
Toxicity Category CTCAE v4.0	***Toxicity Description CTCAE v4.0***				
Blood and lymphatic system disorders	*Anemia*	1	-	-	-
	Thrombocytopenia	1	-	-	-
	Transaminitis	-	-	1	-
Cardiac disorders	*Sinus tachycardia*	1	-	-	-
	Ventricular tachycardia	1	-	-	-
Ear and labyrinth disorders	*Vertigo*	1	-	-	-
Endocrine disorders	*Hyperthyroidism*	2	-	-	-
	Hypothyroidism	1	-	-	-
Eye disorders	*Dry eye*	1	-	-	-
Gastrointestinal disorders	*Abdominal Discomfort*	1	-	-	-
	Abdominal Fullness	1	-	-	-
	Abdominal pain	2	-	-	-
	Colitis	-	1	-	-
	Constipation	4	-	-	-
	Diarrhea	3	-	1	-
	Dry mouth	1	-	-	-
	Dysphagia	1	-	-	-
	Nausea	5	1	-	-
	Vomiting	3	2	-	-
General disorders and admin site conditions	*Fatigue*	3	4	-	-
	Fever	5	1	-	-
	Infusion related reaction	-	1	-	-
	Pain	2	-	-	-
	Right Arm Pain	1	-	-	-
Hepatobiliary disorders	*Hepatitis*	-	-	1	-
Infections and infestations	*Aspiration Pneumonia*	-	-	1	-
	Sepsis	-	-	1	-
	Sinusitis	1	-	-	-
Injury, poisoning and procedural complications	*Fall*	1	-	-	-

		Toxicity Grade CTCAE v4.0			
		01 - Mild	02 - Moderate	03 - Severe	04 - Life-Threatening
		N	N	N	N
Investigations	*Alanine aminotransferase increased*	2	1	2	-
	Alkaline phosphatase increased	1	1	-	-
	Aspartate aminotransferase increased	3	1	2	-
	Decreased RBC	1	-	-	-
	Elevated LDH	2	-	-	-
	Lipase increased	1	-	-	1
	Lymphocyte count decreased	-	1	-	-
	Platelet count decreased	1	-	-	-
	Serum amylase increased	-	-	1	-
	Weight loss	3	1	-	-
	White blood cell decreased	2	-	-	-
Metabolism and nutrition disorders	*Anorexia*	6	-	-	-
	Dehydration	1	-	-	-
	Hyperglycemia	1	1	-	-
	Hypermagnesemia	1	-	-	-
	Hypoalbuminemia	1	-	-	-
	Hypokalemia	-	-	1	-
	Hypophosphatemia	-	1	-	-
	Muscle Soreness	1	-	-	-
Musculoskeletal and connective tissue disorders	*Arthralgia*	2	-	-	-
	Back pain	4	-	-	-
	Generalized muscle weakness	2	-	-	-
	Muscle Cramping (Thigh)	1	-	-	-
	Myalgia	2	-	-	-
	Neck pain	1	-	-	-
	Pain in extremity	-	1	-	-
Nervous system disorders	*Balance Difficulties*	1	-	-	-
	Dizziness	1	1	-	-
	Dysesthesia	1	-	-	-
	Encephalopathy	-	-	1	-
	Headache	1	3	-	-
	Peripheral motor neuropathy	-	1	-	-
	Tingling, Hands	1	-	-	-
Psychiatric disorders	*Confusion*	1	-	-	-
	Insomnia	2	-	-	-
Renal and urinary disorders	*Acute kidney injury*	-	-	1	-
	Urine discoloration	1	-	-	-

		Toxicity Grade CTCAE v4.0			
		01 - Mild	02 - Moderate	03 - Severe	04 - Life-Threatening
		N	N	N	N
Respiratory, thoracic and mediastinal disorders	Cough	2	-	-	-
	Dyspnea	1	-	-	-
	Respiratory, thoracic and mediastinal disorders	1	-	-	-
	Wheezing	1	-	-	-
Skin and subcutaneous tissue disorders	Bilateral Upper Extremity, Right Groin, Right Lower Back and Right Ankle Rash	-	1	-	-
	Dry skin	1	-	-	-
	Erythema Nodosum (Bilateral Legs)	1	-	-	-
	Erythema of left breast.	1	-	-	-
	Pruritic Rash on Chest, Back, Neck, Shoulders, and Bilateral Upper Legs	-	1	-	-
	Pruritus	4	-	-	-
	Rash maculo-papular	4	2	-	-
Vascular disorders	Hypotension	-	-	1	-

Supplementary Table 5. Grade-3 or higher adverse events listed by patient

Patient number	***Description***	***Grade***
3	Hepatitis	3
	Alanine aminotransferase increased	3
	Aspartate aminotransferase increased	3
4	Aspiration Pneumonia	3
	Alanine aminotransferase increased	3
	Aspartate aminotransferase increased	3
11	Diarrhea	3
13	Transaminitis	3
	Sepsis	3
	Acute kidney injury	3
	Hypotension	3
14	Serum amylase increased	3
	Serum lipase increased	4
16	Hypokalemia	3
	Encephalopathy	3

Supplementary Note 1

NCI Protocol #: **N/A**

DF/HCC Protocol #: [16-136]

TITLE: Phase II Trial of Ipilimumab and Nivolumab in Leptomeningeal Metastases

Coordinating Center: Massachusetts General Hospital

***Principal Investigator (PI):**

Priscilla Brastianos
Massachusetts General Hospital
55 Fruit Street, Yawkey 9E
pbrastianos@partners.org

Ryan Sullivan, MD
MGH Cancer Center
Boston, MA
RSULLIVAN7@mgh.harvard.edu

Other Investigators:

Daniel P. Cahill, MD, PhD
Massachusetts General Hospital
Boston, MA

Elizabeth Gerstner, MD
Massachusetts General Hospital
Boston, MA

Kevin Oh, MD
Massachusetts General Hospital
Boston, MA

Sandro Santagata, MD, PhD
Dana-Farber Cancer Institute
Boston, MA

Statistician:
Anita Giobbie-Hurder
Department of Biostatistics
& Computational Biology
Dana-Farber Cancer Institute

Study Coordinator:
Panha Kosal
Massachusetts General Hospital
Yawkey 9E, 55 Fruit Street
Telephone: 617-643-6221
Fax: 617-643-7715
pkosal@mgh.harvard.edu

Responsible Research Nurse
Nicola Gribbin, RN
Massachusetts General Hospital
Yawkey 9E, 55 Fruit Street
Telephone: 617-726-9579
Fax: 617-643-0572
NGRIBBIN@PARTNERS.ORG

Responsible Data Manager:
Panha Kosal
Massachusetts General Hospital
Yawkey 9E, 55 Fruit Street
Telephone: 617-643-6221
Fax: 617-643-7715
pkosal@mgh.harvard.edu

Agents: ipilimumab and nivolumab

Below, please describe the IND Status of this study by choosing IND #/Sponsor OR Exemption from IND requirements, making sure to delete the inapplicable field(s).

IND #: *IND Exempt*
IND Sponsor: Priscilla Brastianos, MD

Protocol Type / Version # / Version Date:[Version 7.0 / Version Date: 24 January 2019]

SCHEMA

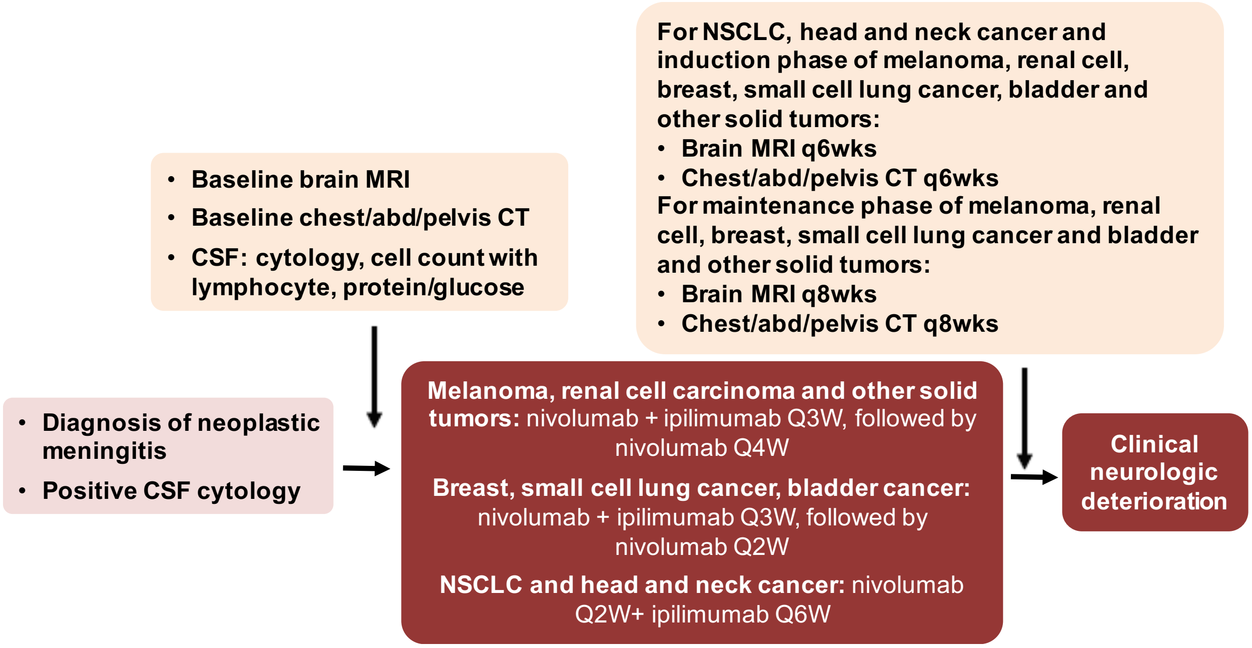
For NSCLC, head and neck cancer and induction phase of melanoma, renal cell, breast, small cell lung cancer, bladder and other solid tumors:
• Brain MRI q6wks
• Chest/abd/pelvis CT q6wks
For maintenance phase of melanoma, renal cell, breast, small cell lung cancer and bladder and other solid tumors:
• Brain MRI q8wks
• Chest/abd/pelvis CT q8wks
• Baseline brain MRI
• Baseline chest/abd/pelvis CT
• CSF: cytology, cell count with lymphocyte, protein/glucose
• Diagnosis of neoplastic meningitis
• Positive CSF cytology
Melanoma, renal cell carcinoma and other solid tumors: nivolumab + ipilimumab Q3W, followed by nivolumab Q4W
Breast, small cell lung cancer, bladder cancer: nivolumab + ipilimumab Q3W, followed by nivolumab Q2W
NSCLC and head and neck cancer: nivolumab Q2W+ ipilimumab Q6W
Clinical neurologic deterioration

TABLE OF CONTENTS

SCHEMA 3

1. OBJECTIVES 7
   - 1.1 Study Design 7
   - 1.2 Primary Objectives 7
   - 1.3 Secondary Objectives 7
   - 1.4 Correlative Objectives 7

2. BACKGROUND 7
   - 2.1 Study Disease(s) 7
   - 2.2 IND Agents 8
   - 2.3 Rationale 15
   - 2.4 Correlative Studies Background 16

3. PARTICIPANT SELECTION 17
   - 3.1 Eligibility Criteria 17
   - 3.2 Exclusion Criteria 19
   - 3.3 Inclusion of Women and Minorities 20

4. REGISTRATION PROCEDURES 20
   - 4.1 General Guidelines for DF/HCC Institutions 20
   - 4.2 Registration Process for DF/HCC Institutions 21
   - 4.3 General Guidelines for Other Investigative Sites 21
   - 4.4 Registration Process for Other Investigative Sites 21

5. TREATMENT PLAN 21
   - 5.1 Treatment Regimen 21
   - 5.2 Pre-Treatment Criteria 22
   - 5.3 Agent Administration 23
   - 5.4 General Concomitant Medication and Supportive Care Guidelines 24
   - 5.5 Criteria for Taking a Participant Off Protocol Therapy 24
   - 5.6 Duration of Follow Up 25
   - 5.7 Criteria for Taking a Participant Off Study 25

6. DOSING DELAYS/DOSE MODIFICATIONS 25

7. ADVERSE EVENTS: LIST AND REPORTING REQUIREMENTS 30
   - 7.1 Adverse Event Characteristics 30
   - 7.2 Expedited Adverse Event Reporting 31
   - 7.3 Expedited Reporting to Hospital Risk Management 31
   - 7.4 Routine Adverse Event Reporting 31
   - 7.5 Definitions 32
   - 7.6 Potential Drug Induced Liver Injury (DILI) 33

7.7 Pregnancy....33
7.8 Overdose....34
7.9 Other Safety Considerations....34
7.10 Adverse event reporting to BMS....34

8. PHARMACEUTICAL INFORMATION....36
8.1 Product Description of Ipilimumab and Nivolumab....36
Sponsor/Investigator drug destruction is allowed provided the following minimal standards are met:....38
• On-site disposal practices must not expose humans to risks from the drug.....38
• On-site disposal practices and procedures are in agreement with applicable laws and regulations, including any special requirements for controlled or hazardous substances.....38
• Written procedures for on-site disposal are available and followed. The procedures must be filed with the Sponsor SOPs and a copy provided to BMS upon request....38
• Note vials and remaining IP are sent to destruction after dose preparation in the IV room as per site SOP. Whole vials are signed out on the accountability logs by the research pharmacy staff.....38
• Accountability and disposal records are complete, up-to-date, and available for BMS to review throughout the clinical trial period as per the study agreement.....38
If conditions for destruction cannot be met, please contact BMS.....38
It is the Sponsor Investigator's responsibility to arrange for disposal of all empty containers, provided that procedures for proper disposal have been established according to applicable federal, state, local, and institutional guidelines and procedures, and provided that appropriate records of disposal are kept....38
8.2 Nivolumab....39
8.3 Ipilimumab....40

9. BIOMARKER, CORRELATIVE, AND SPECIAL STUDIES....41
9.1 Background of exploratory biomarker studies:....42
9.2 Methodology....42
9.3 Collection and Shipping of Tissue Specimen(s)....43

10. STUDY CALENDAR....44

11. MEASUREMENT OF EFFECT....50
11.1 Antitumor Effect – Extracranial lesion assessments....50
11.2 Antitumor Effect – Intracranial lesion assessments....56
11.3 Response Review....61
11.4 Other Response Parameters....61

12. DATA REPORTING / REGULATORY REQUIREMENTS....62
12.1 Data Reporting....63

12.2 Data Safety Monitoring....63
12.3 Multicenter Guidelines....63
12.4 Collaborative Agreements Language....64

13. STATISTICAL CONSIDERATIONS....64
13.1 Study Design/Endpoints....64
13.2 Sample Size, Accrual Rate and Study Duration....64
13.3 Stratification Factors....65
13.4 Interim Monitoring Plan....65
13.5 Analysis of Primary Endpoints....65
13.6 Analysis of Secondary Endpoints....65
13.7 Analysis of Exploratory Endpoints....66
13.8 Reporting and Exclusions....66

14. PUBLICATION PLAN....67

REFERENCES....68

APPENDIX A PERFORMANCE STATUS CRITERIA....70

APPENDIX B Tumor assessment per immune-related response criteria....71

1. OBJECTIVES

1.1 Study Design

This is a Phase II trial of nivolumab and ipilimumab in leptomeningeal carcinomatosis.

1.2 Primary Objectives

To estimate the overall survival of the combination of nivolumab and ipilimumab in leptomeningeal metastases

1.3 Secondary Objectives

1. To describe the toxicity of nivolumab and ipilimumab in brain and leptomeningeal metastases.
2. To estimate the central nervous system response rate (parenchymal brain metastases) of nivolumab and ipilimumab in patients with leptomeningeal disease
3. To estimate the systemic response rate of nivolumab and ipilimumab in patients with brain metastases
4. To estimate leptomeningeal disease response of nivolumab and ipilimumab in patients with leptomeningeal metastases.
5. To estimate the systemic progression free-survival of patients receiving nivolumab and ipilumamb.
6. To estimate the intracranial progression free-survival of patients receiving nivolumab and ipilimumab.

1.4 Correlative Objectives

1. To determine molecular biomarkers of response using next generation sequencing techniques.

2. BACKGROUND

2.1 Study Disease(s)

Approximately 5-8% of patients with cancer develop leptomeningeal carcinomatosis[1]. While virtually any malignancy can metastasize to the leptomeninges, this most commonly occurs with lung cancer, breast cancer and melanoma. Approximately 5-7% of patients with melanoma develop leptomeningeal carcinomatosis and there seems to be an association with the presence of parenchymal brain metastases and the development of leptomeningeal carcinomatosis[2]. As patients with cancer survive longer and diagnostic imaging techniques improve, leptomeningeal carcinomatosis is being diagnosed more frequently. Patients typically present with signs of increased intracranial pressure, cranial nerve palsies, seizures, stroke-like symptoms, encephalopathy, radiculopathies and cauda equina syndrome[1]. The median survival of patients

with leptomeningeal metastases without treatment is approximately 4-6 weeks. Death usually results from rapid progressive neurologic deterioration. Treatment involves radiation to symptomatic sites of the neuraxis and to sites of bulk disease seen on imaging studies. Systemically administered chemotherapy has limited access to the CSF due to the blood-brain barrier. Intrathecal chemotherapy is designed to treat tumor cells floating in the cerebrospinal fluid (CSF), thereby preventing the development of new symptomatic sites of disease; however treatment-related toxicities of intrathecal chemotherapy are common, and include chemical meningitis, seizures and severe headaches[3]. Radiotherapy and intrathecal chemotherapy provide local control and may extend median survival of patients with leptomeningeal carcinomatosis to 3-6 months[3,4]. Novel therapies are needed to improve outcomes in patients with leptomeningeal carcinomatosis.

2.2 IND Agents

2.2.1 Ipilimumab

Ipilimumab (BMS-734016, MDX010, MDX-CTLA4) is a fully human monoclonal immunoglobulin (Ig) G1κ specific for human cytotoxic T lymphocyte antigen 4 (CTLA-4, CD152), which is expressed on a subset of activated T cells. CTLA-4 is a negative regulator of T-cell activity. Ipilimumab is a monoclonal antibody that binds to CTLA-4 and blocks the interaction of CTLA-4 with its ligands, CD80/CD86. Blockade of CTLA-4 has been shown to augment T-cell activation and proliferation, including the activation and proliferation of tumor infiltrating T-effector cells. Inhibition of CTLA-4 signaling can also reduce T-regulatory cell function, which may contribute to a general increase in T-cell responsiveness, including the anti-tumor response. Yervoy™ (ipilimumab) has been approved for use in over 47 countries including the United States (US, Mar-2011), the European Union (EU, Jul-2011), and Australia (Jul-2011).

Nonclinical Studies

Ipilimumab has specificity and a high affinity for human CTLA-4. The calculated dissociation constant value from an average of several studies was 5.25 nM. Binding of ipilimumab to purified, recombinant human CTLA-4 antigen was also demonstrated by enzyme-linked immunosorbent assay with half-maximal binding at 15 ng/mL, whereas saturation was observed at approximately 0.1 μg/mL. No cross-reactivity was observed against human CD28. Ipilimumab completely blocked binding of B7.1 and B7.2 to human CTLA-4 at concentrations higher than 6 μg/mL and 1 μg/mL, respectively.

Effects in Humans

Bristol-Myers Squibb (BMS) and Medarex, Inc. (MDX, acquired by BMS in Sep-2009) have co-sponsored an extensive clinical development program for ipilimumab, encompassing more than 19,500 subjects (total number of subjects enrolled in ipilimumab studies) in several cancer types in completed and ongoing studies, as well as a compassionate use program. The focus of the clinical program is in melanoma, prostate cancer, and lung cancer, with advanced melanoma being the most comprehensively studied indication. Ipilimumab is being investigated both as monotherapy and in combination with other modalities such as chemotherapy, radiation therapy, and other immunotherapies. Phase 3 programs are ongoing in melanoma, prostate cancer, and lung cancer. In melanoma, 2 completed Phase 3 studies (MDX010-20 and CA184024) have

demonstrated a clinically meaningful and statistically significant survival benefit in pretreated advanced melanoma and previously untreated advanced melanoma, respectively. The safety profile of ipilimumab is generally consistent across these trials with a) the majority adverse events (AEs) being inflammatory in nature consistent with the proposed mechanism of action of ipilimumab, b) the same types of such immune-mediated events in the gastrointestinal (GI) tract, skin, liver, and endocrine system being reported, and c) most of these events being manageable with immune suppressive therapies.

In melanoma, 2 BMS-sponsored Phase 3 studies are ongoing in subjects with high-risk Stage III melanoma (CA184029, with adjuvant immunotherapy) and pretreated and treatment-naïve advanced melanoma (CA184169, 3 mg/kg versus 10 mg/kg ipilimumab). The completed Phase 3 study CA184043 evaluated ipilimumab in subjects with metastatic castration-resistant prostate cancer (mCRPC) who had progressed during or following docetaxel. Eligible subjects were randomized to a single dose of bone-directed radiotherapy (RT), followed by either ipilimumab 10 mg/kg or placebo (799 randomized: 399 ipilimumab, 400 placebo). This study did not meet its primary endpoint of overall survival (OS). The hazard ratio (HR) of 0.85 (95% confidence interval [CI]: 0.72 to 1.00) for survival favored ipilimumab but did not reach statistical significance with a P-value of 0.053. Planned sensitivity analyses favored ipilimumab, where the greatest benefit appeared to be in subgroups defined by good prognostic features and low burden of disease. Additional evidence of ipilimumab activity observed in the study included a reduced risk of disease progression relative to placebo (HR = 0.70) and superior clinical outcomes compared to placebo in tumor regression and declines in prostate specific antigen (PSA). The safety profile in this study was consistent with the previously defined AE profile at the same dose.

A second Phase 3 study CA184095 evaluating ipilimumab 10 mg/kg versus placebo in men with asymptomatic or minimally symptomatic, chemotherapy-naïve mCRPC with no visceral metastases has completed enrollment and is currently in follow-up. In addition, a completed large Phase 2 study (CA184041) has investigated the addition of ipilimumab to carboplatin and paclitaxel using 2 different schedules (concurrent and phased) in subjects with non-small cell lung cancer (NSCLC) or small cell lung cancer (SCLC, a secondary endpoint). Ipilimumab, given in combination with paclitaxel/carboplatin in a phased schedule improved irPFS compared to the control treatment, but no improvement was seen when ipilimumab was given in a concurrent schedule. Phased ipilimumab also improved PFS according to mWHO criteria and showed a trend for improved OS. The efficacy and safety of ipilimumab in a phased schedule with carboplatin/paclitaxel is also being investigated in an ongoing Phase 3 study in subjects with advanced squamous NSCLC (CA184104). The efficacy and safety of ipilimumab in a phased schedule with etoposide/platinum in subjects with extensive stage disease SCLC is being investigated in an ongoing Phase 3 study (CA184156).

The unique immune-based mechanism of action is reflected in the clinical patterns of anti-cancer activity in some patients. Ipilimumab induces an immunologic response, and measurable clinical effects emerge after the immunological effects. Tumor infiltration with lymphocytes and the associated inflammation (documented by biopsy in some subjects) is likely the cornerstone of the effect of ipilimumab and can manifest in various patterns of clinical activity leading to tumor control. In some cases, inflammation may not be noted by radiological examination and objective response is observed with the first tumor assessment in a manner seen in patients receiving other

types of anti-cancer treatments. In other cases, response may be preceded by an apparent increase in initial tumor volume and/or the appearance of new lesions, which may be mistaken for tumor progression on radiological evaluations. Therefore, in patients who are not experiencing rapid clinical deterioration, confirmation of progression is recommended, at the investigator's discretion, to better understand the prognosis as well as to avoid unnecessarily initiating potentially toxic alternative therapies in subjects who might be benefiting from treatment. Immune-related response criteria were developed based on these observations to systematically categorize novel patterns of clinical activity and are currently being prospectively evaluated in clinical studies. In metastatic diseases, stabilization is more common than response, and in some instances is associated with a slow, steady decline in tumor burden over many months, sometimes improving to partial and/or complete responses. Thus, the immune-based mechanism of action of ipilimumab results in durable disease control, sometimes with novel patterns of response, which contribute to its unique improvement in OS.

The unique immune-based mechanism of action is also reflected in the safety profile. The most common treatment-related AEs are inflammatory in nature, consistent with the mechanism of action of the drug and generally medically manageable with topical and/or systemic immunosuppressants. Such immunological safety events are described as immune-related adverse events (irAEs) or immune-mediated adverse reactions (imARs). The irAEs are described as AEs of unknown etiology, which were consistent with an immune phenomenon and were considered causally related to drug exposure by investigators. The irAEs primarily involve the GI tract and skin. Immune-related AEs in the liver were also observed, particularly in subjects receiving 10 mg/kg. Endocrinopathy and neuropathy were important irAEs observed less frequently. The imARs were adjudicated in a blinded fashion based on sponsor-physician data review to exclude noninflammatory etiologies, such as infection or tumor progression, and to consider available evidence of inflammation, such as tumor biopsies or responsiveness to steroids, in an effort to determine whether specific AEs or abnormal hepatic laboratory values were likely to be immune mediated and associated with ipilimumab treatment. The early diagnosis of inflammatory events is important to initiate therapy and minimize complications. Inflammatory events are generally manageable using symptomatic or immuno-suppressive therapy as recommended through detailed diagnosis and management guidelines.

A program-wide independent Data Monitoring Committee (DMC) reviews data from the ipilimumab studies, allowing for an ongoing safety and benefit/risk assessment in subjects receiving ipilimumab. The DMC charter includes explicit stopping rules for some studies, allowing the DMC to recommend discontinuing further treatment across the ipilimumab program, if necessary. In summary, ipilimumab offers clinically meaningful and statistically significant survival benefit to patients with pretreated advanced melanoma (as 3 mg/kg monotherapy compared to the melanoma peptide vaccine gp100) and previously untreated advanced melanoma (at 10 mg/kg in combination with dacarbazine [DTIC] compared to DTIC alone), and evidence of clinical activity in randomized studies in other tumor types. These findings, together with evidence of a safety profile that is manageable with careful monitoring and appropriate intervention for treatment of immune-related toxicities, suggest an acceptable benefit to risk ratio.

2.2.2 Nivolumab

Nivolumab (also referred to as BMS-936558 or MDX1106) is a fully human monoclonal antibody (HuMAb; immunoglobulin G4 [IgG4]-S228P) that targets the programmed death-1 (PD-1) cluster of differentiation 279 (CD279) cell surface membrane receptor. PD-1 is a negative regulatory molecule expressed by activated T and B lymphocytes. Binding of PD-1 to its ligands, programmed death–ligands 1 (PD-L1) and 2 (PD-L2), results in the down-regulation of lymphocyte activation. Inhibition of the interaction between PD-1 and its ligands promotes immune responses and antigen-specific T-cell responses to both foreign antigens as well as self-antigens. Nivolumab is expressed in Chinese hamster ovary (CHO) cells and is produced using standard mammalian cell cultivation and chromatographic purification technologies. The clinical study product is a sterile solution for parenteral administration (Nivolumab Investigator Brochure).

Nonclinical Studies

Nivolumab has been shown to bind specifically to the human PD-1 receptor and not to related members of the CD28 family, such as CD28 inducible co-stimulator (ICOS), cytotoxic T lymphocyte antigen-4 (CTLA-4), and B and T lymphocyte attenuator (BTLA). Nivolumab inhibits the interaction of PD-1 with its ligands, PD-L1 and PD-L2, resulting in enhanced T-cell proliferation and interferon-gamma (IFN-γ) release in vitro. Fluorescent-activated cell sorter (FACS) analysis confirmed that nivolumab binds to transfected CHO and activated human T-cells expressing cell surface PD-1 and to cynomolgus monkey PD-1, but not to rat or rabbit PD-1 molecules. Nivolumab has also been shown to bind to PD-1 on virus-specific CD8+ T-cells from chronically infected hepatitis C virus patients. PD-1 inhibition in a mixed lymphocyte reaction (MLR) resulted in a reproducible concentration-dependent enhancement of IFN-γ release in the MLR up to 50 g/mL. No effect was observed with a human IgG4 isotype control or CD4+ T cells and dendritic cell (DC) controls.

In intravenous (IV) repeat-dose toxicology studies in cynomolgus monkeys, nivolumab was well tolerated at doses up to 50 mg/kg, administered weekly for 5 weeks, and at doses up to 50 mg/kg, administered twice weekly for 27 doses. Nivolumab-related findings were limited to a reversible decrease of 28% in triiodothyronine (T3) among the females administered 27 doses of 50 mg/kg. No corresponding changes in the level of thyroxin (T4), thyroid-stimulating hormone (TSH), or histologic changes in the thyroid were observed. While nivolumab alone was well tolerated in cynomolgus monkeys, combination studies have highlighted the potential for enhanced toxicity when combined with other immunostimulatory agents.

Ipilimumab (BMS-734016), an anti-CTLA-4 monoclonal antibody (mAb) that blocks the down-regulation of T-cell activation, was used in combination with nivolumab to investigate the effects of concurrent inhibition of the PD-1 and CTLA-4 receptors in nonhuman primates. Although gastrointestinal (GI) toxicity has not been observed in cynomolgus monkeys treated with nivolumab alone, dose-dependent GI toxicity was evident in cynomolgus monkeys treated weekly for 4 weeks with a combination of nivolumab + ipilimumab at combinations of 10 and 3 mg/kg and 50 and 10 mg/kg, respectively. GI effects have also been observed at a low incidence after ipilimumab administration.13 Two 4-week toxicity studies in cynomolgus monkeys were conducted with 2 anti-lymphocyte-activation gene 3 (LAG-3) mAbs that inhibit the function of LAG-3 on the surface of activated CD4+ and CD8+ T cells. The combination of nivolumab +

MDX1408 was clinically well tolerated up to 50 mg/kg MDX1408 and 50 mg/kg nivolumab with findings limited to nonadverse and pharmacologically-mediated changes in the peripheral blood and lymphoid tissues, including increases in CD4+ T lymphocytes expressing CD25. However, the combination of 50 mg/kg nivolumab + 100mg/kg BMS-986016, administered once weekly, was associated with moribundity (1 of 10 animals) attributed to central nervous system (CNS) vasculitis. Findings in surviving monkeys administered nivolumab alone or in combination with BMS-986016 were limited to minimal inflammation of the choroid plexus or vasculature of the brain parenchyma (1 male in the combination group), without evidence of other degenerative changes. These findings, which were observed at nivolumab exposures that were approximately 13× greater than those observed in humans at 3 mg/kg, every 2 weeks (Q2W), are consistent with an expected immunostimulatory pharmacologic effect of nivolumab and highlight the potential synergistic roles of PD-1 and LAG-3 in maintaining self-tolerance. In addition, an enhanced pre- and postnatal development (ePPND) study in pregnant cynomolgus monkeys with nivolumab was conducted. Administration of nivolumab at up to 50 mg/kg 2QW was well tolerated by pregnant monkeys; however, nivolumab was determined to be a selective developmental toxicant when administered from the period of organogenesis to parturition at ≥ 10 mg/kg (area under the concentration-time curve [AUC] from time zero to 168 hours [AUC(0-168 h)] 117,000 μg•h/mL). Specifically, increased developmental mortality (including late gestational fetal losses and extreme prematurity with associated neonatal mortality) was noted in the absence of overt maternal toxicity. There were no nivolumab-related changes in surviving infants tested throughout the 6-month postnatal period. Although the cause of these pregnancy failures was undetermined, nivolumab-related effects on pregnancy maintenance are consistent with the established role of PD-L1 in maintaining fetomaternal tolerance in mice.

Effects in Humans

The PK, clinical activity, and safety of nivolumab have been assessed in completed Phase 1 and ongoing Phase 2 and 3 studies sponsored by Bristol-Myers Squibb Company (BMS) in subjects with non-small cell lung cancer (NSCLC), melanoma, and clear-cell renal cell carcinoma (RCC) in addition to other tumor types. The current Phase 3 clinical program focuses on squamous and nonsquamous NSCLC, malignant melanoma, and RCC. Clinical activity and safety information presented in this Investigator Brochure (IB) focuses primarily on that obtained from CA209063 (Phase 2 study in refractory squamous NSCLC), CA209037 (Phase 3 study in melanoma), MDX1106-03 (also known as CA209003; Phase 1 study in metastatic NSCLC, colorectal cancer (CRC), melanoma, RCC, or metastatic castrate-resistant prostate cancer [mCRPC]), CA209010 (Phase 2 study in advanced/metastatic clear-cell RCC). Data are also provided from Phase 1 studies CA209004 (also known as MDX1106-04), CA209012, CA209016, CA209038, and CA209039. Nivolumab is being investigated both as monotherapy and in combination with chemotherapy, targeted therapies, and other immunotherapies.

The single-dose pharmacokinetics (PK) of nivolumab was linear and dose-proportional in the range of 0.3 mg/kg to 10 mg/kg. The multiple-dose PK of nivolumab was linear with dose-proportional increases in maximum serum concentration (Cmax) and area under the concentration-time curve over the dosing interval (AUC[TAU]) in the range of 0.1 mg/kg to 10 mg/kg. Both elimination and distribution of nivolumab in the dose range studied appear to be independent of dose in the dose-ranging studies, while the end of infusion and minimum serum concentration

(Cmin) after the first dose were approximately dose proportional. Based on population PK (PPK) results (preliminary data), clearance of nivolumab is independent of dose in the dose range (0.1 mg/kg to 10 mg/kg) and tumor types studied. Body weight normalized dosing showed approximately constant trough concentrations over a wide range of body weights and, therefore, is appropriate for future clinical trials with nivolumab.

Nivolumab has demonstrated clinical activity as monotherapy and as combination therapy with ipilimumab in several tumor types, including RCC, melanoma, NSCLC, and some lymphomas. The majority of responses were durable and exceeded 6 months.

The overall safety experience with nivolumab, as a monotherapy or in combination with other therapeutics, is based on experience in approximately 4,000 subjects treated to date. For monotherapy, the safety profile is similar across tumor types. The only exception is pulmonary inflammation adverse events (AEs), which may be numerically greater in subjects with NSCLC, because in some cases, it can be difficult to distinguish between nivolumab-related and unrelated causes of pulmonary symptoms and radiographic changes. There is no pattern in the incidence, severity, or causality of AEs to nivolumab dose level. In several ongoing clinical trials, the safety of nivolumab in combination with other therapeutics such as ipilimumab, cytotoxic chemotherapy, anti-angiogenics, and targeted therapies is being explored. Most studies are ongoing and, as such, the safety profile of nivolumab combinations continues to evolve. The most advanced combination under development is nivolumab + ipilimumab in subjects with melanoma. Thus far, the combination of both agents results in a safety profile with similar types of AEs as either agent alone, but in some cases with a greater frequency.

2.2.3 Nivolumab combined with Ipilimumab

Preclinical Data

Preclinical data indicate that the combination of PD-1 and CTLA-4 receptor blockade may improve antitumor activity. In vitro combinations of nivolumab plus ipilimumab increase IFN-γ production 2- to 7-fold over either agent alone in a mixed lymphocyte reaction. Increased antitumor activity of the combination was also observed in 3 of 5 syngeneic murine cancer models. In a murine melanoma vaccine model, blockade with either CTLA-4 or PD-1 antibodies increased the proportion of CTLA-4 and PD-1-expressing CD4/CD8 tumor infiltrating T effector cells and dual blockade increased tumor infiltration of T effector cells and decreased intratumoral T regulatory cells, as compared to either agent alone.

Preclinically, a 4-week toxicity study of nivolumab in combination with ipilimumab conducted in cynomolgus monkeys demonstrated that the combination of nivolumab and ipilimuma resulted in dose-dependent gastrointestinal (GI) toxicity. Histologic findings included inflammatory changes in the large intestine, which increased in incidence and severity in a dosedependent manner. GI toxicity/colitis was not observed in cynomolgus monkeys administered nivolumab alone, but was observed in monkeys receiving ipilimumab. Nivolumab in combination with ipilimumab was also associated with lymphoid hypocellularity of the cortex and/or medulla of the thymus and with acinar cell degranulation in the pancreas. Additional findings included interstitial mononuclear cell infiltrates in the kidneys, portal mononuclear cell infiltrates in the liver and myeloid hypercellularity in the bone marrow. Nivolumab in combination with ipilimumab at the high-dose level (ie, 50 mg/kg and 10 mg/kg, respectively) was associated with the death of 1 animal,

attributed to acute gastric dilatation without histopathological evidence of colitis upon pathology evaluation of the GI tract.

Clinical Data

In the Phase 1 study CA209004, ascending doses of nivolumab have been studied concomitantly with ascending doses of ipilimumab in subjects with unresectable or metastatic melanoma. In each arm in this multi-arm study, ipilimumab was administered once every 3 weeks for 4 doses with nivolumab administered once every 3 weeks for 8 doses. Starting at week 24, ipilimumab and nivolumab were administered once every 12 weeks for 8 doses. The three initial dose escalation cohorts consisted of Cohort 1 (nivolumab 0.3 mg/kg plus ipilimumab 3 mg/kg; n = 14), Cohort 2 (nivolumab 1 mg/kg plus ipilimumab 3 mg/kg; n = 17) and Cohort 3 (nivolumab 3 mg/kg plus ipilimumab 3 mg/kg; n = 6). Later, the study was amended to include Cohort 2a which evaluated nivolumab 3 mg/kg plus ipilimumab 1 mg/kg (n = 16) and Cohort 8 that represents an additional cohort of patients treated with nivolumab 1 mg/kg plus ipilimumab 3 mg/kg The maintenance regimen for Cohorts 1-3 consisted of nivolumab q3 weeks x 4 then nivolumab plus ipilimumab q12 weeks x 8, while the maintenance regimen for Cohort 8 consisted of nivolumab 3 mg/kg q2 weeks until progression. The following dose limiting toxicities (DLTs) were observed in Cohort 1 - Grade 3 elevated AST/ALT (1 subject); in Cohort 2 - Grade 3 uveitis (1 subject) and Grade 3 elevated AST/ALT (1 subject) and in Cohort 3 Grade 4 elevated lipase (2 subjects) and Grade 3 elevated lipase (1 subject). Based on these data, Cohort 2 was identified as the maximum tolerated dose (MTD) and Cohort 3 exceeded the MTD. Among the 53 patients in the concurrent-regimen group, adverse events of any grade, regardless of whether they were attributed to the therapy, were observed in 98% of patients.

Treatment-related adverse events of any grade were reported in 96.2% of patients, in pooled Cohorts 1-3 and 95.1% of Cohort 8. The drug-related skin select AE category was the most frequently reported category in all cohorts; rash and pruritus were the most frequently reported drug-related AEs in all cohorts. The most frequently reported drug-related AEs of any grade were rash (62.3%, Cohorts 1-3; 58.5%, Cohort 8), pruritus (56.6%, Cohorts 1-3; 46.3% Cohort 8), diarrhea (41.5%, Cohorts 1-3; 31.7%, Cohort 8), fatigue (41.5%, Cohorts 1-3; 41.5%, Cohort 8), lipase increased (26.4%, Cohorts 1-3; 14.6%, Cohort 8), AST increased (24.5%, Cohorts 1-3; 9.8%, Cohort 8), ALT increased (22.6%, Cohorts 1-3; 12.2%, Cohort 8), nausea (22.6%, Cohorts 1-3; 24.4%, Cohort 8), pyrexia (22.6%, Cohorts 1-3; 22.0%, Cohort 8), amylase increased (20.8%, Cohorts 1-3; 12.2%, Cohort 8). The only reported drug-related AE belonging to the pulmonary select AE category was pneumonitis; 3 (5.7%) subjects in Cohorts 1 to 3 and 1 (2.4%) subject in Cohort 8. Grade 3-4 events were reported in 1 subject each in Cohort 2a and Cohort 8. Treatment-related SAEs of any grade were reported in 58.5% of patients on pooled Cohorts 1-3 and 41.5 % of Cohort 8. The most frequently reported SAE in ≥ 5% subjects included: ALT increased, AST increased, lipase increased, colitis, diarrhea, amylase increased, pneumonitis, and pyrexia. A total of 13 subjects (24.5%) in Cohorts 1-3 and 9 subjects (22.0%) in Cohort 8 discontinued therapy due to treatment-related adverse events. The majority of the deaths were due to disease progression in CA209004. One subject died due to drug-related enterocolitis. The only CNS treatment related AE reported in >10% of subjects was headache [11.3% Cohorts 1-3 (Grade 1-2); 12.2% Cohort 8 with 1 Grade 3-4 event (2.4%)]. A single case of radiation necrosis was observed in a subject who received SRT for progression in the brain while on nivolumab monotherapy maintenance dosing.

The subject had completed 4 doses of induction with combination ipilimumab and nivolumab, and 3 doses of nivolumab monotherapy maintenance. He received SRT for progression between dose #3 and dose #4 of nivolumab maintenance. He subsequently received a single dose of combination maintenance. Approximately 5-6 weeks after the dose of combination maintenance, radionecrosis was diagnosed. Attribution was assessed as related to SRT and unrelated to study drug. Three subjects experienced drug-related CNS adverse events that led to discontinuation - one subject with blurred vision (Grade 1) and headache (Grade 2), one subject with presyncope (Grade 3) and one subject with ageusia (Grade 1). A single subject experienced CNS adverse events assessed as not related, that led to discontinuation: muscular weakness (Grade 3), dizziness (Grade 2), headache (Grade 2), confusion (Grade 2). None of these subjects had received prior brain radiotherapy. Of the 11 subjects who did receive prior radiotherapy to the brain, only 1 subject experienced a CNS adverse event which was the case of radiation necrosis described above.

Because of the potential for clinically meaningful nivolumab or ipilimumab related AEs requiring early recognition and prompt intervention, management algorithms have been developed for suspected pulmonary toxicity, GI, hepatotoxicity, endocrinopathy, skin toxicity, neurological toxicity and renal toxicity. Prompt interventions are recommended according to the management algorithms. In order to standardize the management, for the overlapping adverse event management algorithms present in both the nivolumab and ipilimumab IB (GI, hepatic, and endocrine algorithms), the recommendations are to follow the BMS-936558 (nivolumab) IB adverse event algorithms and not the ipilimumab IB algorithms. The algorithms recommended for utilization in CA209204 are contained in the nivolumab (BMS-936558) IB. As of August, 2014, 3 subjects out of approximately 4000 patients on nivolumab clinical trials have developed opportunistic infections (2 cases of Aspergillus pneumonia, and 1 case of Pneumocystis jiroveci pneumonia) after receiving prolonged treatment with high dose steroids for nivolumab-related adverse events. Details of these cases are available in the Investigator Brochure.

2.3 Rationale

The treatment of patients with many types of cancers has been revolutionized over the past decade with the development of immune checkpoint inhibitors targeting cytotoxic T-lymphocyte antigen 4 (CTLA4) and the programmed death 1 (PD1) receptor-ligand (PDL1, PDL2) interaction, as well as the development of small molecule inhibitors. These advances have led to the regulatory approval of ipilimumab, nivolumab, and pembrolizumab[5-10]. More recently, the combination of the anti-CTLA4 antibody ipilimumab and the anti-PD1 antibody nivolumab has been associated with deep responses in half of treated patients[11]. In addition to these advances, previously “untreatable” patient cohorts, such as those with brain metastases, have been evaluated in dedicated clinical trials. In fact, both ipilimumab and dabrafenib have demonstrated single-agent central nervous system (CNS) effects, leading to a remarkable loosening of eligibility criteria for melanoma clinical trials that just 5 years ago typically required 3 and up to 12 month stability of CNS metastasis[12,13]. A Phase II study of ipilimumab in patients with asymptomatic melanoma brain metastases showed disease activity in 18% of patients[14]. Although a clinical trial of nivolumab in brain metastases has not yet been published, a retrospective study demonstrated that patients with brain metastases receiving the combination of nivolumab and stereotactic radiation had improved survival compared to surgery and/or radiation alone[15]. An ongoing study by Goldberg and Kluger

et al.[13,14] showed that pembrolizumab is well tolerated and has promising activity in patients with asymptomatic brain metastases from melanoma and non-small cell lung cancer (NSCLC). Of 12 evaluable patients with melanoma brain metastases, partial responses were observed in 3 patients and stable disease in 2 patients[14]. Of 9 evaluable patients with brain metastases from nonsmall cell lung cancer (NSCLC), partial responses were observed in 4 patients[13]. Now, early phase trials often allow patients with asymptomatic and untreated metastases to enroll, and nearly all therapies deemed promising in melanoma are being tested in dedicated phase II trials for patients with untreated brain metastases.

As astounding as these developments are, there remains one group of patients who remain ineligible for virtually every therapeutic trial, those with leptomeningeal disease (LMD). While any malignancy can metastasize to the leptomeninges, this most commonly occurs with lung cancer, breast cancer and melanoma. Approximately 5-8% of patients with cancer develop leptomeningeal carcinomatosis[1]. As visceral metastatic disease is better controlled and patients are surviving longer, LMD is being diagnosed more frequently. The median survival of patients with LMD without treatment is dismal, typically 4-6 weeks. Novel therapies are needed to improve outcomes in patients with LMD. The absence of effective therapies, and more importantly, access to treatment as part of clinical trials is a great unmet need in cancer.

A recent study demonstrated that ipilimumab has activity in patients with brain metastases; however and not surprisingly, patients with LMD were excluded from this study. In our experience at Massachusetts General Hospital (MGH) Cancer Center, we have observed 2 clinical responses in the approximately 10 patients with melanoma and LMD who have received ipilimumab. Moreover there was one published report of describing a patient with leptomeningeal disease from melanoma having a complete response after receiving ipilimimab[16]. Furthermore, in a separate PI-initiated trial (DF/HCC #16-153), an interim analysis of 18 patients with LMD treated with pembrolizumab showed that primary endpoint has been met, and more than 8 patients survived more than 3 months. The role of immunotherapy has not been systematically explored in this patient population, and based on our limited but encouraging experience with our patients, we have proposed a Phase II trial of ipilimumab plus nivolumab in patients with LMD, using a dose schedule that has demonstrated efficacy in other tumors. This trial offers a unique opportunity to systematically study LMD at the genomic and immune microenvironmental levels before and during therapy.

2.4 Correlative Studies Background

Genetic heterogeneity is a major limitation to the identification of predictors of response to nonCNS and CNS metastases, as well as LMD. We propose to evaluate recurrent clinically relevant mutations and genetic heterogeneity in non-CNS metastases and LMD. We will collect primary (when possible) and non-CNS metastatic tumors, and CSF cytology specimens as part of this clinical trial and perform whole exome and transcriptome sequencing of these samples. Of note, patients with LMD have Ommaya/VP shunts, thus allowing for frequent sampling of CSF. We will create phylogenetic trees to understand the evolutionary patterns in the metastatic process to the leptomeninges. In cases where we have primary tumors, we will compare mutational profiles between different sites. To date we have demonstrated our ability to perform these assays through

next generation sequencing of 101 CNS metastases matched with primary tumors and other distal metastatic sites. We will use these same techniques to understand the genomic evolution of LMD, and to identify genomic biomarkers of response to immunotherapy.

The tumor microenvironment is a complex system that includes tumor cells, stromal elements, infiltrating immune cells, and host tissue (e.g. liver in hepatic metastases), all interacting in ways that influence the effectiveness of any therapy, especially immunotherapy. Our groups have performed studies of the immune characteristics of the tumor microenvironment before and after checkpoint blockade. To date, there have been no descriptions of the CSF/LM tumor microenvironment in LMD, either unperturbed or after immunotherapy. We will be collecting CSF from every patient in this study. Lumbar punctures are part of standard of care in patients with leptomeningeal carcinomatosis. Serial CSF samples will give us the opportunity to study tumor evolution over the course of treatment with ipilimumab and nivolumab. Here we will perform unbiased transcriptome sequencing of purified CD4+ and CD8+ T cells and CD11b+ myeloid cells from the CSF to define the effects of immunotherapy on LMD in the patients described above. We will also evaluate expression of cytokines and other proteins in the CSF to further examine how checkpoint blockade alters the CSF/LM microenvironment. In parallel, we will purify the same immune cell types from paired PBMC samples for comparative transcriptomic analyses and collect sera for comparative cytokine/proteomic analyses with CSF. These comparative analyses should identify if there are alterations in the blood that reflect the biology of the CSF/LM microenvironment and can serve as biomarkers.

3. PARTICIPANT SELECTION

3.1 Eligibility Criteria

3.1.1 Participants must have histologically or cytologically confirmed disease from any solid tumor.

3.1.2 Age ≥18 years.

3.1.3 ECOG performance status ≤ 2 (Karnofsky ≥60%, see Appendix A)

3.1.4 Life expectancy of greater than 3 weeks

3.1.5 Participants must have normal organ and marrow function as defined in Table 1, all screening labs should be performed within 14 days of treatment initiation.

Table 1 Adequate Organ Function Laboratory Values

System	Laboratory Value
**Hematological**	
Absolute neutrophil count (ANC)	≥1500 /mcL
Platelets	≥100,000 / mcL

Hemoglobin	≥9 g/dL or ≥5.6 mmol/L without transfusion or EPO dependency (within 7 days of assessment)
**Renal**	
Serum creatinine **OR** Measured or calculated[a] creatinine clearance (GFR can also be used in place of creatinine or CrCl)	≤ Serum creatinine ≤ 1.5 x ULN or creatinine clearance (CrCl) ≥ 40 mL/min (if using the Cockcroft-Gault formula below): $\textit{Female}\ \text{CrCl} = \frac{(140 - \text{age in years}) \times \text{weight in kg} \times 0.85}{72 \times \text{serum creatinine in mg/dL}}$ $\textit{Male}\ \text{CrCl} = \frac{(140 - \text{age in years}) \times \text{weight in kg} \times 1.00}{72 \times \text{serum creatinine in mg/dL}}$
**Hepatic**	
Serum total bilirubin	≤ 1.5 X ULN **OR**
	Direct bilirubin ≤ ULN for subjects with total bilirubin levels > 1.5 ULN
AST (SGOT) and ALT (SGPT)	≤ 3 ULN **OR** ≤ 5 X ULN for subjects with liver metastases
Albumin	≥2.5 mg/dL
**Coagulation**	
International Normalized Ratio (INR) or Prothrombin Time (PT)	≤1.5 X ULN unless subject is receiving anticoagulant therapy as long as PT or PTT is within therapeutic range of intended use of anticoagulants
Activated Partial Thromboplastin Time (aPTT)	≤1.5 X ULN unless subject is receiving anticoagulant therapy as long as PT or PTT is within therapeutic range of intended use of anticoagulants
[a]Creatinine clearance should be calculated per institutional standard.	

3.1.6 Women of childbearing potential (WOCBP) must use appropriate method(s) of contraception. WOCBP should use an adequate method to avoid pregnancy for 23 weeks (30 days plus the time required for nivolumab to undergo five half-lives) after the last dose of investigational drug.

3.1.7. Women of childbearing potential must have a negative serum or urine pregnancy test (minimum sensitivity 25 IU/L or equivalent units of HCG) within 72 hours prior to the start of nivolumab.

3.1.8. Women must not be breastfeeding.

3.1.9. Men who are sexually active with WOCBP must use any contraceptive method with a failure rate of less than 1% per year. Men receiving nivolumab and who are sexually active with WOCBP will be instructed to adhere to contraception for a period of 31 weeks after the last dose of investigational product. Women who are not of childbearing potential, ie, who are postmenopausal or surgically sterile as well as azoospermic men do not require contraception

3.1.7 Ability to understand and the willingness to sign a written informed consent document.

3.1.8 Stable dose of dexamethasone 2 mg or less for 7 days prior to initiation of treatment

3.1.9 Carcinomatous meningitis, as defined by positive cytology or biopsy. If CSF cytology is negative, but imaging is consistent with carcinomatous meningitis, patient is eligible.

3.2 Exclusion Criteria

3.2.1 Participants who have had chemotherapy, targeted small molecule therapy or study therapy within 5 days of protocol treatment, or those who have not recovered (i.e., ≤ Grade 1 or at baseline) from adverse events due to agents administered more than 2 weeks earlier. Subjects with ≤ Grade 2 neuropathy are an exception to this criterion and may qualify for the study. If subject received major surgery, they must have recovered adequately from the toxicity and/or complications from the intervention prior to starting therapy.

3.2.2 Participants who are receiving any other investigational agents.

3.2.3 Patients should be excluded if they have an active, known or suspected autoimmune disease. Subjects are permitted to enroll if they have vitiligo, type I diabetes mellitus, residual hypothyroidism due to autoimmune condition only requiring hormone replacement, psoriasis not requiring systemic treatment, or conditions not expected to recur in the absence of an external trigger

3.2.4 Patients should be excluded if they have a condition requiring systemic treatment with either corticosteroids (> 10 mg daily prednisone equivalents) or other immunosuppressive medications within 14 days of study drug administration. Inhaled or topical steroids and adrenal replacement doses > 10 mg daily prednisone equivalents are permitted in the absence of active autoimmune disease. Subjects are permitted to use topical, ocular, intra-articular, intranasal, and inhalational corticosteroids (with minimal systemic absorption). Physiologic replacement doses of systemic corticosteroids are permitted, even if > 10 mg/day prednisone equivalents. A brief course of corticosteroids for prophylaxis (eg, contrast dye allergy) or for treatment of non-autoimmune conditions (eg, delayed-type hypersensitivity reaction caused by contact allergen) is permitted.

3.2.5 As there is potential for hepatic toxicity with nivolumab or nivolumab/ipilimumab combinations, drugs with a predisposition to hepatoxicity should be used with caution in patients treated with nivolumab-containing regimen.

3.2.6 Patients should be excluded if they have had prior systemic treatment with a combination of anti-CTLA4 antibody and anti-PD1/PDL1 antibody. Prior treatment with single agent anti-CTLA4, anti-PD1 or anti-PDL1 antibody is allowed.

3.2.7 Has a known history of active TB (Bacillus Tuberculosis)

3.2.8 Patients should be excluded if they are positive test for hepatitis B virus surface antigen (HBV sAg) or hepatitis C virus ribonucleic acid (HCV antibody) indicating acute or chronic infection

3.2.9 Patients should be excluded if they have known history of testing positive for human immunodeficiency virus (HIV) or known acquired immunodeficiency syndrome (AIDS). These participants are at increased risk of lethal infections when treated with marrow-suppressive therapy. Appropriate studies will be undertaken in participants receiving combination antiretroviral therapy when indicated.

3.2.10 Uncontrolled intercurrent illness including, but not limited to, ongoing or active infection, symptomatic congestive heart failure, unstable angina pectoris, cardiac arrhythmia, or psychiatric illness/social situations that would limit compliance with study requirements.

3.2.11 Has known psychiatric or substance abuse disorders that would interfere with cooperation with the requirements of the trial.

3.2.12 Is pregnant or breastfeeding, or expecting to conceive or father children within the projected duration of the trial, starting with the pre-screening or screening visit through 120 days after the last dose of trial treatment.

3.2.13 Has a known additional malignancy that is progressing or requires active treatment. Exceptions include basal cell carcinoma of the skin or squamous cell carcinoma of the skin that has undergone potentially curative therapy or in situ cervical cancer.

3.2.14 Has known history of, or any evidence of active, non-infectious pneumonitis.

3.2.15 Has an active infection requiring systemic therapy.

3.2.16 Has received a live vaccine within 30 days of planned start of study therapy. Note: Seasonal influenza vaccines for injection are generally inactivated flu vaccines and are allowed; however intranasal influenza vaccines (e.g., Flu-Mist®) are live attenuated vaccines, and are not allowed.

3.2.17 History of allergy to study drug components

3.2.18 History of severe hypersensitivity reaction to any monoclonal antibody

3.3 Inclusion of Women and Minorities

Both men and women of all races and ethnic groups are eligible for this trial.

4. REGISTRATION PROCEDURES

4.1 General Guidelines for DF/HCC Institutions

Institutions will register eligible participants in the Clinical Trials Management System (CTMS) OnCore. Registrations must occur prior to the initiation of protocol therapy. Any participant not registered to the protocol before protocol therapy begins will be considered ineligible and registration will be denied.

An investigator will confirm eligibility criteria and a member of the study team will complete the protocol-specific eligibility checklist.

Following registration, participants may begin protocol therapy. Issues that would cause treatment delays should be discussed with the Overall Principal Investigator (PI). If a participant does not receive protocol therapy following registration, the participant's registration on the study must be canceled. Registration cancellations must be made in OnCore as soon as possible.

4.2 Registration Process for DF/HCC Institutions

DF/HCC Standard Operating Procedure for Human Subject Research Titled *Subject Protocol Registration* (SOP #: REGIST-101) must be followed.

4.3 General Guidelines for Other Investigative Sites

N/A

4.4 Registration Process for Other Investigative Sites

N/A

5. TREATMENT PLAN

5.1 Treatment Regimen by histology:

For melanoma: patients will be treated with the combination regimen of nivolumab 1 mg/kg combined with ipilimumab 3mg/kg Q3W (4 doses), followed by nivolumab monotherapy (480 mg Q4W) until progression or unacceptable toxicity.

For nonsmall cell lung cancer and head and neck cancer: patients will be treated with nivolumab 3mg/kg Q2W + ipilimumab 1mg/kg Q6W

For small cell lung cancer, breast cancer, and bladder cancer: patients will be treated with nivolumab 1mg/kg + ipilimumab 3mg/kg Q3W x 4 doses, followed by nivolumab 240 mg Q2W

For renal cell carcinoma and other solid tumors (not listed above): patients will be treated with nivolumab 3mg/kg + ipilimumab 1mg/kg Q3W x 4 doses, followed by nivolumab 480 mg Q4W

Each treatment cycle will be 6 weeks, with the exception of the nivolumab maintenance monotherapy phase in melanoma, renal cell carcinoma and other solid tumors, when each cycle

will be 8 weeks. Treatment will be administered on an outpatient basis. Reported adverse events and potential risks are described in Section 7. Appropriate dose modifications are described in Section 6. No investigational or commercial agents or therapies other than those described below may be administered with the intent to treat the participant's malignancy.

5.2 Pre-Treatment Criteria

5.2.1 Cycle 1, Day 1

A serum or urine pregnancy testing is required within 72 hrs of study enrollment. Cycle 1, Day 1 labs will be used to confirm eligilibity.

System	Laboratory Value
**Hematological**	
Absolute neutrophil count (ANC)	≥1500 /mcL
Platelets	≥100,000 / mcL
Hemoglobin	≥9 g/dL or ≥5.6 mmol/L without transfusion or EPO dependency (within 7 days of assessment)
**Renal**	
Serum creatinine **<u>OR</u>**<br>Measured or calculated[a] creatinine clearance<br>(GFR can also be used in place of creatinine or CrCl)	≤ Serum creatinine ≤ 1.5 x ULN or creatinine clearance (CrCl) ≥ 40 mL/min (if using the Cockcroft-Gault formula below):<br>*Female* CrCl = $\frac{\text{(140 - age in years) x weight in kg x 0.85}}{\text{72 x serum creatinine in mg/dL}}$<br>*Male* CrCl = $\frac{\text{(140 - age in years) x weight in kg x 1.00}}{\text{72 x serum creatinine in mg/dL}}$
**Hepatic**	
Serum total bilirubin	≤ 1.5 X ULN **<u>OR</u>**
	Direct bilirubin ≤ ULN for subjects with total bilirubin levels > 1.5 ULN
AST (SGOT) and ALT (SGPT)	≤ 3 ULN **<u>OR</u>**<br>≤ 5 X ULN for subjects with liver metastases
Albumin	≥2.5 mg/dL
**Coagulation**	
International Normalized Ratio (INR) or Prothrombin Time (PT)<br><br>Activated Partial Thromboplastin Time (aPTT)	≤1.5 X ULN unless subject is receiving anticoagulant therapy as long as PT or PTT is within therapeutic range of intended use of anticoagulants<br>≤1.5 X ULN unless subject is receiving anticoagulant therapy as long as PT or PTT is within therapeutic range of intended use of anticoagulants

[a]Creatinine clearance should be calculated per institutional standard.

5.2.2 Subsequent Cycles

Pregnancy Test: Every 6 weeks. After discontinuation from nivolumab these should be repeated at approximately 30 days and approximately 70 days.

Laboratory testing prior to each dose: Within 72 hrs prior to re-dosing to include CBC w/ differential, LFTs, BUN or serum urea level, creatinine, Ca, Mg, Na, K, Cl, LDH, Glucose, amylase, lipase, TSH (with reflexive Free T4 and Free T3)

System	Laboratory Value
**Hematological**	
Absolute neutrophil count (ANC)	≥1500 /mcL
Platelets	≥100,000 / mcL
Hemoglobin	≥9 g/dL or ≥5.6 mmol/L without transfusion or EPO dependency (within 7 days of assessment)
**Renal**	
Serum creatinine **OR** Measured or calculated[a] creatinine clearance (GFR can also be used in place of creatinine or CrCl)	≤ Serum creatinine ≤ 1.5 x ULN or creatinine clearance (CrCl) ≥ 40 mL/min (if using the Cockcroft-Gault formula below): *Female* CrCl = $\frac{(140 - \text{age in years}) \times \text{weight in kg} \times 0.85}{72 \times \text{serum creatinine in mg/dL}}$ *Male* CrCl = $\frac{(140 - \text{age in years}) \times \text{weight in kg} \times 1.00}{72 \times \text{serum creatinine in mg/dL}}$

5.3 Agent Administration

For treatment schedule, see section 5.1

When study drugs (ipilimumab or nivolumab) are to be administered on the same day, separate infusion bags and filters must be used for each infusion. It is recommended that nivolumab be administered first. The second infusion will always be ipilimumab, and will start approximately 30 minutes after completion of the nivolumab infusion. The ipilimumab infusion will start no SOONER than 30 minutes after completion of nivolumab infusion. An infusion window of +/- 10 minutes is allowed for both study drugs Nivolumab & Ipilimumab.

BMS-936558 (nivolumab) is to be administered as a 30 minute IV infusion. Ipilimumab should be administered as a 30 minute infusion following.

Ipilimumab and nivolumab may be diluted in 0.9% Sodium Chloride Solution or 5% Dextrose solution.

The dosing calculations should be based on the body weight. If the subject's weight on the day of dosing differs by > 5% from the weight used to calculate the dose, the dose must be recalculated. All doses should be rounded up or to the nearest milligram per institutional standard.

For the q3 week schedule, patients may be dosed no less than 18 days from the previous dose of drug; and dosed up to 3 days after the scheduled date if necessary.

For the q4 week schedule, patients may be dosed no less than 24 days from the previous dose of drug; and dosed up to 3 days after the scheduled date if necessary.

There will be no dose modifications permitted. Dose reductions or dose escalations are not permitted.

5.4 General Concomitant Medication and Supportive Care Guidelines

Antiemetic premedications should not be routinely administered prior to dosing of drugs. See Section 6 for premedication recommendations following a nivolumab or ipilimumab related infusion reaction.

Note: Radiation therapy to a symptomatic single extracranial site and/or one intracranial site may be allowed at the investigator's discretion, as long as ipilimumab is held during radiation and at least 3 weeks after completion of radiation.

5.5 Criteria for Taking a Participant Off Protocol Therapy

Duration of therapy will depend on individual response, evidence of disease progression and tolerance. In the absence of treatment delays due to adverse event(s), treatment may continue for until one of the following criteria applies:

- Disease progression
- Intercurrent illness that prevents further administration of treatment
- Unacceptable adverse event(s)
- Participant demonstrates an inability or unwillingness to comply with the oral medication regimen and/or documentation requirements
- Participant decides to withdraw from the protocol therapy
- General or specific changes in the participant's condition render the participant unacceptable for further treatment in the judgment of the treating investigator

Participants will be removed from the protocol therapy when any of these criteria apply. The reason for removal from protocol therapy, and the date the participant was removed, must be documented in the case report form (CRF). Alternative care options will be discussed with the participant.

A Office of Data Quality (ODQ) Treatment Ended/Off Study Form will be filled out when a participant is removed from protocol therapy. This form can be found on the ODQ website or obtained from the ODQ registration staff.

In the event of unusual or life-threatening complications, treating investigators must immediately notify the Overall PI, Priscilla Brastianos at 617-724-8563.

5.6 Duration of Follow Up

Patients will have a followup visit 30 days from the last dose +/- 7 days or coincide with the date of discontinuation (+/- 7 days) if date of discontinuation is greater than 37 days after last dose. Follow-up visit 2 will be 8-10 weeks (+/- 7 days) from follow-up visit 1. After 2 followup visits, patients will be followed with a phone call every 10-12 weeks for survival information for 2 years after removal from protocol therapy or until death, whichever occurs first. Participants removed from protocol therapy for unacceptable adverse event(s) will be followed until resolution or stabilization of the adverse event.

5.7 Criteria for Taking a Participant Off Study

Participants will be removed from study when any of the following criteria apply:

- Lost to follow-up
- Withdrawal of consent for data submission
- Death

The reason for taking a participant off study, and the date the participant was removed, must be documented in the case report form (CRF).

An ODQ Treatment Ended/Off Study Form will be filled out when a participant comes off study. This form can be found on the ODQ website or obtained from the ODQ registration staff.

6. DOSING DELAYS/DOSE MODIFICATIONS

The descriptions and grading scales found in the revised NCI Common Terminology Criteria for Adverse Events (CTCAE) version 4.0 will be utilized for dose delays and dose modifications. A copy of the CTCAE version 4.0 can be downloaded from the CTEP website http://ctep.cancer.gov/protocolDevelopment/electronic_applications/ctc.htm.

There will be no dose modifications permitted. Dose reductions or dose escalations are not permitted.

Management Algorithms for Immuno-Oncology Agents

Immuno-oncology (I-O) agents are associated with adverse events that can differ in severity and duration than adverse events caused by other therapeutic classes. Nivolumab and ipilimumab are considered immuno-oncology agents in this protocol. Management algorithms have been developed to assist investigators in assessing and managing the following groups of adverse events: Gastrointestinal, Renal, Pulmonary, Hepatic, Endocrinopathies, Skin, and Neurological.

Early recognition and intervention are recommended according to the management algorithms; and in addition include ophthalmologic evaluations for any visual symptoms in order to evaluate for nivolumab or ipilimumab related uveitis.

The recommendations are to follow the algorithms in the nivolumab investigator brochure for immune related events; while the ipilimumab investigator brochure contains similar algorithms, the algorithms in the nivolumab brochure have been aligned to accommodate combinations as well as nivolumab monotherapy.

For subjects expected who require more than 4 weeks of corticosteroids or other immunosuppressants to manage an adverse event, consider the following recommendations

- Antimicrobial/antifungal prophylaxis per institutional guidelines to prevent opportunistic infections such as Pneumocystis jiroveci and fungal infections.
- Early consultation with an infectious disease specialist should be considered. Depending on the presentation, consultation with a pulmonologist for bronchoscopy or a gastroenterologist for endoscopy may also be appropriate.
- In patients who develop recurrent adverse events in the setting of ongoing or prior immunosuppressant use, an opportunistic infection should be considered in the differential diagnosis.

Additional details on the safety of nivolumab and ipilimumab , including results from clinical studies, are available in the IB.

Dose Delay Criteria

Because of the potential for clinically meaningful nivolumab-related AEs requiring early recognition and prompt intervention, management algorithms have been developed for suspected AEs of selected categories. [see current Investigator Brochure for citation examples]

Dose delay criteria apply for all drug-related adverse events (regardless of whether or not the event is attributed to nivolumab, ipilimumab or both). All study drugs must be delayed until treatment can resume.

Nivolumab and ipilimumab administration should be delayed for the following:

- Any Grade ≥ 2 non-skin, drug-related adverse event, with the following exceptions:
    - Grade 2 drug-related fatigue or laboratory abnormalities do not require a treatment delay
- Any Grade 3 skin, drug-related adverse event
- Any Grade 3 drug-related laboratory abnormality, with the following exceptions for asymptomatic amylase or lipase, AST, ALT, or total bilirubin:
    - Grade 3 amylase or lipase abnormalities that are not associated with symptoms or clinical manifestations of pancreatitis do not require a dose delay. It is recommended to consult with the principle investigator for Grade 3 amylase or lipase abnormalities.
    - If a subject has a baseline AST, ALT, or total bilirubin that is within normal limits, delay dosing for drug-related Grade ≥ 2 toxicity
    - If a subject has baseline AST, ALT, or total bilirubin within the Grade 1 toxicity range, delay dosing for drug-related Grade ≥ 3 toxicity
- Any adverse event, laboratory abnormality, or intercurrent illness which, in the judgment of the investigator, warrants delaying the dose of study medication.

Criteria to Resume Treatment

Subjects may resume treatment with study drug when the drug-related AE(s) resolve to Grade ≤1 or baseline value, with the following exceptions:

- Subjects may resume treatment in the presence of Grade 2 fatigue
- Subjects who have not experienced a Grade 3 drug-related skin AE may resume treatment in the presence of Grade 2 skin toxicity
- Subjects with baseline Grade 1 AST/ALT or total bilirubin who require dose delays for reasons other than a 2-grade shift in AST/ALT or total bilirubin may resume treatment in the presence of Grade 2 AST/ALT OR total bilirubin
- Subjects with combined Grade 2 AST/ALT AND total bilirubin values meeting discontinuation parameters should have treatment permanently discontinued
- Drug-related pulmonary toxicity, diarrhea, or colitis, must have resolved to baseline before treatment is resumed

- Drug-related endocrinopathies adequately controlled with only physiologic hormone replacement may resume treatment

If the criteria to resume treatment are met, the subject should restart treatment at the next scheduled timepoint per protocol. However, if the treatment is delayed past the next scheduled timepoint per protocol, the next scheduled timepoint will be delayed until dosing resumes. If treatment is delayed > 6 weeks, the subject must be permanently discontinued from study therapy, except as specified in discontinuation section.

<u>Discontinuation Criteria</u>

Treatment should be permanently discontinued for the following:

- Any Grade 2 drug-related uveitis or eye pain or blurred vision that does not respond to topical therapy and does not improve to Grade 1 severity within the re-treatment period OR requires systemic treatment
- Any Grade 3 non-skin, drug-related adverse event lasting > 7 days, with the following exceptions for drug-related laboratory abnormalities, uveitis, pneumonitis, bronchospasm, diarrhea, colitis, neurologic adverse event, hypersensitivity reactions, and infusion reactions
    - Grade 3 drug-related uveitis, pneumonitis, bronchospasm, colitis, neurologic adverse event, hypersensitivity reaction, or infusion reaction of any duration requires discontinuation
    - Grade 3 drug-related laboratory abnormalities do not require treatment discontinuation except those noted below
        - Grade 3 drug-related thrombocytopenia > 7 days or associated with bleeding requires discontinuation
        - Any drug-related liver function test (LFT) abnormality that meets the following criteria require discontinuation:
            - AST or ALT > 8 x ULN
            - Total bilirubin > 5 x ULN
            - Concurrent AST or ALT > 3 x ULN and total bilirubin > 2 x ULN
- Any Grade 4 drug-related adverse event or laboratory abnormality, except for the following events which do not require discontinuation:
    - Isolated Grade 4 amylase or lipase abnormalities that are not associated with symptoms or clinical manifestations of pancreatitis and decrease to < Grade 4 within 1 week of onset.

- Isolated Grade 4 electrolyte imbalances/abnormalities that are not associated with clinical sequelae and are corrected with supplementation/appropriate management within 72 hours of their onset

- Any dosing interruption lasting > 6 weeks with the following exceptions:
  - Dosing interruptions to allow for prolonged steroid tapers to manage drug-related adverse events are allowed. Prior to re-initiating treatment in a subject with a dosing interruption lasting > 6 weeks, the Investigator must be consulted. Tumor assessments should continue as per protocol even if dosing is interrupted
  - Dosing interruptions > 6 weeks that occur for non-drug-related reasons may be allowed if approved by the Investigator. Prior to re-initiating treatment in a subject with a dosing interruption lasting > 6 weeks, the Investigator must be consulted. Tumor assessments should continue as per protocol even if dosing is interrupted
- Any adverse event, laboratory abnormality, or intercurrent illness which, in the judgment of the Investigator, presents a substantial clinical risk to the subject with continued nivolumab or ipilimumab dosing

Treatment of Nivolumab or Ipilimumab Related Infusion Reactions

Since nivolumab and ipilimumab contain only human immunoglobulin protein sequences, it is unlikely to be immunogenic and induce infusion or hypersensitivity reactions. However, if such a reaction were to occur, it might manifest with fever, chills, rigors, headache, rash, pruritis, arthralgias, hypo- or hypertension, bronchospasm, or other symptoms.

All Grade 3 or 4 infusion reactions should be reported as an SAE if criteria are met. Infusion reactions should be graded according to NCI CTCAE 4.0 guidelines.

Treatment recommendations are provided below and may be modified based on local treatment standards and guidelines as appropriate:

For Grade 1 symptoms: (Mild reaction; infusion interruption not indicated; intervention not indicated)

Remain at bedside and monitor subject until recovery from symptoms. The following prophylactic premedications are recommended for future infusions: diphenhydramine 50 mg (or equivalent) and/or paracetamol 325 to 1000 mg (acetaminophen) at least 30 minutes before additional nivolumab administrations.

For Grade 2 symptoms: (Moderate reaction requires therapy or infusion interruption but responds promptly to symptomatic treatment [eg, antihistamines, non-steroidal anti inflammatory drugs, narcotics, corticosteroids, bronchodilators, IV fluids]; prophylactic medications indicated for 24 hours).

Stop the nivolumab or ipilimumab infusion, begin an IV infusion of normal saline, and treat the subject with diphenhydramine 50 mg IV (or equivalent) and/or paracetamol 325 to 1000 mg (acetaminophen); remain at bedside and monitor subject until resolution of symptoms.

Corticosteroid or bronchodilator therapy may also be administered as appropriate. If the infusion is interrupted, then restart the infusion at 50% of the original infusion rate when symptoms resolve; if no further complications ensue after 30 minutes, the rate may be increased to 100% of the original infusion rate. Monitor subject closely. If symptoms recur then no further nivolumab or ipilimumab will be administered at that visit. Administer diphenhydramine 50 mg IV, and remain at bedside and monitor the subject until resolution of symptoms. The amount of study drug infused must be recorded on the electronic case report form (eCRF). The following prophylactic premedications are recommended for future infusions: diphenhydramine 50 mg (or equivalent) and/or paracetamol 325 to 1000 mg (acetaminophen) should be administered at least 30 minutes before additional nivolumab or ipilimumab administrations. If necessary, corticosteroids (recommended dose: up to 25 mg of IV hydrocortisone or equivalent) may be used.

For Grade 3 or Grade 4 symptoms: (Severe reaction, Grade 3: prolonged [ie, not rapidly responsive to symptomatic medication and/or brief interruption of infusion]; recurrence of symptoms following initial improvement; hospitalization indicated for other clinical sequelae [eg, renal impairment, pulmonary infiltrates]). Grade 4: (life threatening; pressor or ventilatory support indicated).

Immediately discontinue infusion of nivolumab or ipilimumab. Begin an IV infusion of normal saline, and treat the subject as follows. Recommend bronchodilators, epinephrine 0.2 to 1 mg of a 1:1,000 solution for subcutaneous administration or 0.1 to 0.25 mg of a 1:10,000 solution injected slowly for IV administration, and/or diphenhydramine 50 mg IV with methylprednisolone 100 mg IV (or equivalent), as needed. Subject should be monitored until the investigator is comfortable that the symptoms will not recur. Nivolumab or ipilimumab will be permanently discontinued. Investigators should follow their institutional guidelines for the treatment of anaphylaxis. Remain at bedside and monitor subject until recovery from symptoms. In the case of late-occurring hypersensitivity symptoms (eg, appearance of a localized or generalized pruritis within 1 week after treatment), symptomatic treatment may be given (eg, oral antihistamine, or corticosteroids).

7. ADVERSE EVENTS: LIST AND REPORTING REQUIREMENTS

Adverse event (AE) monitoring and reporting is a routine part of every clinical trial. The following list of reported and/or potential AEs (Section 7.1) and the characteristics of an observed AE (Section 7.2) will determine whether the event requires expedited reporting **in addition** to routine reporting.

7.1 Adverse Event Characteristics

- **CTCAE term (AE description) and grade:** The descriptions and grading scales found in the revised NCI Common Terminology Criteria for Adverse Events (CTCAE) version 4.0 will be utilized for AE reporting. All appropriate treatment areas should have access to a copy of the CTCAE version 4.0. A copy of the CTCAE version 4.0 can be downloaded from the CTEP web site http://ctep.cancer.gov/protocolDevelopment/electronic_applications/ctc.htm.

- **For expedited reporting purposes only:**
  - AEs for the agent(s) that are listed above should be reported only if the adverse event varies in nature, intensity or frequency from the expected toxicity information which is provided.
  - Other AEs for the protocol that do not require expedited reporting are outlined in the next section (Expedited Adverse Event Reporting) under the sub-heading of Protocol-Specific Expedited Adverse Event Reporting Exclusions.

- **Attribution** of the AE:
  - Definite – The AE *is clearly related* to the study treatment.
  - Probable – The AE *is likely related* to the study treatment.
  - Possible – The AE *may be related* to the study treatment.
  - Unlikely – The AE *is doubtfully related* to the study treatment.
  - Unrelated – The AE *is clearly NOT related* to the study treatment.

7.2 Expedited Adverse Event Reporting

7.2.1 Investigators **must** report to the Overall PI any serious adverse event (SAE) that occurs after the initial dose of study treatment, during treatment, or within 30 days of the last dose of treatment on the local institutional SAE form.

7.2.2 DF/HCC Expedited Reporting Guidelines

Investigative sites within DF/HCC will report AEs directly to the DFCI Office for Human Research Studies (OHRS) per the DFCI IRB reporting policy.

7.3 Expedited Reporting to Hospital Risk Management

Participating investigators will report to their local Risk Management office any participant safety reports or sentinel events that require reporting according to institutional policy.

7.4 Routine Adverse Event Reporting

All Adverse Events **must** be reported in routine study data submissions to the Overall PI on the toxicity case report forms. **AEs reported through expedited processes (e.g., reported to the IRB, FDA, etc.) must also be reported in routine study data submissions.**

7.5 Definitions

7.5.1 An **Adverse Event (AE)** is defined as any new untoward medical occurrence or worsening of a preexisting medical condition in a clinical investigation subject administered an investigational (medicinal) product and that does not necessarily have a causal relationship with this treatment. An AE can therefore be any unfavorable and unintended sign (such as an abnormal laboratory finding), symptom, or disease temporally associated with the use of investigational product, whether or not considered related to the investigational product. The causal relationship to study drug is determined by a physician and should be used to assess all adverse events (AE).

7.5.2 A **S*erious Adverse Event (SAE)*** is any untoward medical occurrence that at any dose:

- results in death
- is life-threatening (defined as an event in which the subject was at risk of death at the time of the event; it does not refer to an event which hypothetically might have caused death if it were more severe)
- requires inpatient hospitalization or causes prolongation of existing hospitalization (see **NOTE** below)
- results in persistent or significant disability/incapacity
- is a congenital anomaly/birth defect
- is an important medical event (defined as a medical event(s) that may not be immediately life-threatening or result in death or hospitalization but, based upon appropriate medical and scientific judgment, may jeopardize the subject or may require intervention [eg, medical, surgical] to prevent one of the other serious outcomes listed in the definition above.) Examples of such events include, but are not limited to, intensive treatment in an emergency room or at home for allergic bronchospasm; blood dyscrasias or convulsions that do not result in hospitalization.)
- Potential drug induced liver injury (DILI) is also considered an important medical event.
- Suspected transmission of an infectious agent (eg, pathogenic or nonpathogenic) via the study drug is an SAE.
- Although pregnancy, overdose, and cancer are not always serious by regulatory definition, these events must be handled as SAEs.

The following hospitalizations are not considered SAEs:

- a visit to the emergency room or other hospital department < 24 hours, that does not result in admission (unless considered an important medical or life-threatening event)
- elective surgery, planned prior to signing consent
- admissions as per protocol for a planned medical/surgical procedure
- routine health assessment requiring admission for baseline/trending of health status (eg, routine colonoscopy)

- Medical/surgical admission other than to remedy ill health and planned prior to entry into the study. Appropriate documentation is required in these cases
- Admission encountered for another life circumstance that carries no bearing on health status and requires no medical/surgical intervention (eg, lack of housing, economic inadequacy, caregiver respite, family circumstances, administrative reason).

7.5.3 Nonserious Adverse Events

- A ***nonserious adverse event*** is an AE not classified as serious.
- Nonserious Adverse Events are to be provided to BMS in aggregate via interim or final study reports as specified in the agreement or, if a regulatory requirement [e.g. IND US trial] as part of an annual reporting requirement.
- Nonserious AE information should also be collected from the start of a placebo lead-in period or other observational period intended to establish a baseline status for the subjects.

7.6 Potential Drug Induced Liver Injury (DILI)

Wherever possible, timely confirmation of initial liver-related laboratory abnormalities should occur prior to the reporting of a potential DILI event. All occurrences of potential DILIs, meeting the defined criteria, must be reported as SAEs. Potential drug induced liver injury is defined as:

1) ALT or AST elevation > 3 times upper limit of normal (ULN)
   AND
2) Total bilirubin > 2 times ULN, without initial findings of cholestasis (elevated serum alkaline phosphatase)
   AND
3) No other immediately apparent possible causes of AST/ALT elevation and hyperbilirubinemia, including, but not limited to, viral hepatitis, pre-existing chronic or acute liver disease, or the administration of other drug(s) known to be hepatotoxic.

7.7 Pregnancy

If, following initiation of the investigational product, it is subsequently discovered that a study subject is pregnant or may have been pregnant at the time of investigational product exposure, including during at least 6 half lives after product administration, the investigational product will be permanently discontinued in an appropriate manner (eg, dose tapering if necessary for subject safety).

The investigator must immediately notify Worldwide Safety @BMS of this event via the Pregnancy Surveillance Form in accordance with SAE reporting procedures.

Follow-up information regarding the course of the pregnancy, including perinatal and neonatal outcome and, where applicable, offspring information must be reported on the Pregnancy Surveillance Form [provided upon request from BMS]

Any pregnancy that occurs in a female partner of a male study participant should be reported to BMS. Information on this pregnancy will be collected on the Pregnancy Surveillance Form.

7.8 Overdose

An overdose is defined as the accidental or intentional administration of any dose of a product that is considered both excessive and medically important. All occurrences of overdose must be reported as an SAE.

7.9 Other Safety Considerations

Any significant worsening noted during interim or final physical examinations, electrocardiograms, x-rays, and any other potential safety assessments, whether or not these procedures are required by the protocol, should also be recorded as a nonserious or serious AE, as appropriate, and reported accordingly.

7.10 Adverse event reporting to BMS

- All Serious Adverse Events (SAEs) that occur following the subject's written consent to participate in the study through 100 days of discontinuation of dosing must be reported to BMS Worldwide Safety.
- The BMS SAE form should be used to report SAEs. If the BMS form cannot be used, another acceptable form (i.e CIOMS or Medwatch) must be reviewed and approved by BMS. The BMS protocol ID number must be included on whatever form is submitted by the Sponsor/Investigator.
- Following the subject's written consent to participate in the study, all SAEs, whether related or not related to study drug, are collected, including those thought to be associated with protocol-specified procedures. The investigator should report any SAE occurring after these time periods that is believed to be related to study drug or protocol-specified procedure.
- In accordance with local regulations, BMS will notify investigators of all reported SAEs that are suspected (related to the investigational product) and unexpected (i.e., not previously described in the IB). In the European Union (EU), an event meeting these criteria is termed a Suspected, Unexpected Serious Adverse Reaction (SUSAR). Investigator notification of these events will be in the form of an expedited safety report (ESR).
    - Other important findings which may be reported by the as an ESR include: increased frequency of a clinically significant expected SAE, an SAE considered associated with study procedures that could modify the conduct of the study, lack of efficacy that poses significant hazard to study subjects, clinically significant safety finding from a nonclinical (eg, animal) study, important safety recommendations from a study data monitoring committee, or sponsor decision to end or temporarily halt a clinical study for safety reasons.

  - Upon receiving an ESR from BMS, the investigator must review and retain the ESR with the IB. Where required by local regulations or when there is a central IRB/IEC for the study, the sponsor will submit the ESR to the appropriate IRB/IEC. The investigator and IRB/IEC will determine if the informed consent requires revision. The investigator should also comply with the IRB/IEC procedures for reporting any other safety information.
  - In addition, suspected serious adverse reactions (whether expected or unexpected) shall be reported by BMS to the relevant competent health authorities in all concerned countries according to local regulations (either as expedited and/or in aggregate reports).

- Following the subject's written consent to participate in the study, all SAEs, whether related or not related to study drug, must be collected, including those thought to be associated with protocol-specified procedures. All SAEs must be collected that occur within 100 days of discontinuation of dosing.
- All SAEs must be collected that occur during the screening period. If applicable, SAEs must be collected that relate to any protocol-specified procedure (eg, a follow-up skin biopsy). The investigator should report any SAE that occurs after these time periods that is believed to be related to study drug or protocol-specified procedure.
- SAEs, whether related or not related to study drug, and pregnancies must be reported to BMS within 24 hours. SAEs must be recorded on BMS or an approved form; pregnancies on a Pregnancy Surveillance Form.
- **SAE Email Address:** Worldwide.Safety@BMS.com
- **SAE Facsimile Number:** 609-818-3804
- If only limited information is initially available, follow-up reports are required. (Note: Follow-up SAE reports should include the same investigator term(s) initially reported.)
- If an ongoing SAE changes in its intensity or relationship to study drug or if new information becomes available, a follow-up SAE report should be sent within 24 hours to the BMS (or designee) using the same procedure used for transmitting the initial SAE report.
- All SAEs should be followed to resolution or stabilization.
- The Sponsor/Investigator will ensure that all SAEs in the clinical database are reported to BMS and any applicable health authority during the conduct of the study. This reconciliation will occur at least quarterly and be initiated by the sponsor/investigator. Sponsor/investigator will request a reconciliation report from: aepbusinessprocess@bms.com. During reconciliation, any events found to not be reported previously to BMS must be sent to Worldwide.Safety@BMS.com.
- An SAE report should be completed for any event where doubt exists regarding its seriousness.

- If the investigator believes that an SAE is not related to study drug, but is potentially related to the conditions of the study (such as withdrawal of previous therapy or a complication of a study procedure), the relationship should be specified in the narrative section of the SAE Report Form.
- If only limited information is initially available, follow-up reports are required. (Note: Follow-up SAE reports should include the same investigator term(s) initially reported.)
- If an ongoing SAE changes in its intensity or relationship to study drug or if new information becomes available, a follow-up SAE report should be sent within 24 hours to BMS using the same procedure used for transmitting the initial SAE report. All SAEs should be followed to resolution or stabilization. All SAEs should be followed to resolution or stabilization.

8. PHARMACEUTICAL INFORMATION

8.1 Product Description of Ipilimumab and Nivolumab

**Table**	**Product Description**				
**Product Description and Dosage Form**	**Potency**	**Primary Packagin g (Volume)/ Label Type**	**Secondary Packaging (Qty) /Label Type**	**Appearance**	**Storage Conditions (per label)**
Nivolumab BMS-936558-01 Solution for Injection[a]	100 mg (10 mg/mL)	10 mL viall	5 vials per carton/ Open-label	Clear to opalescent colorless to pale yellow liquid. May contain particles	2 to 8°C. Protect from light and freezing.
Ipilimumab Solution for Injection	200 mg (5 mg/mL)	40 mL vial	4 vials per carton/Open-label	Clear, colorless to pale yellow liquid. May contain particles	2 to 8°C. Protect from light and freezing.

8.1.1 Storage and Stability

If stored in a glass front refrigerator, vials should be stored in the carton. Recommended safety measures for preparation and handling of Nivolumab and Ipilimumab include laboratory coats and gloves.

For additional details on prepared drug storage and use time of Nivolumab or Ipilimumab under room temperature/light and refrigeration, please refer to the BMS-936558 (Nivolumab) and Ipilimumab Investigator Brochure section for “Recommended Storage and Use Conditions”

8.1.2 **Handling**

The investigator should ensure that the study drug is stored in accordance with the environmental conditions (temperature, light, and humidity) as per product information and the Investigator Brochure and per local regulations. It is the responsibility of the investigator to ensure that investigational product is only dispensed to study subjects. The investigational product must be dispensed only from official study sites by authorized personnel according to local regulations. If concerns regarding the quality or appearance of the study drug arise, the study drug should not be dispensed and contact BMS immediately.

Please refer to the current version of the Investigator Brochure and/or shipment reference sheets for additional information on storage, handling, dispensing, and infusion information for Nivolumab.

8.1.3 **Preparation**

See Section 8.2 for Nivolumab instructions and 8.3 for Ipilimumab instructions.

8.1.4 **Administration**

See Section 8.2 for Nivolumab instructions and 8.3 for Ipilimumab instructions.

8.1.5 **Accountability**

The investigator is responsible for ensuring that the investigational product is stored under the appropriate environmental conditions (temperature, light, and humidity)

- If concerns regarding the quality or appearance of the investigational product arise, do not dispense the investigational product, and contact BMS immediately. If commercial investigational product is used, it should be stored in accordance with the appropriate local labeling
- If the study drug(s) are to be destroyed on site, it is the investigator's responsibility to ensure that arrangements have been made for disposal, and that procedures for proper disposal have been established according to applicable regulations, guidelines, and institutional procedures

8.1.6 **Destruction and Return**

Sponsor/Investigator drug destruction is allowed provided the following minimal standards are met:

- On-site disposal practices must not expose humans to risks from the drug.
- On-site disposal practices and procedures are in agreement with applicable laws and regulations, including any special requirements for controlled or hazardous substances.
- Written procedures for on-site disposal are available and followed. The procedures must be filed with the Sponsor SOPs and a copy provided to BMS upon request.
- Note vials and remaining IP are sent to destruction after dose preparation in the IV room as per site SOP. Whole vials are signed out on the accountability logs by the research pharmacy staff.
- Accountability and disposal records are complete, up-to-date, and available for BMS to review throughout the clinical trial period as per the study agreement.

If conditions for destruction cannot be met, please contact BMS.

It is the Sponsor Investigator's responsibility to arrange for disposal of all empty containers, provided that procedures for proper disposal have been established according to applicable federal, state, local, and institutional guidelines and procedures, and provided that appropriate records of disposal are kept.

8.1.7 **Ordering**

Initial Orders

- Following submission and approval of the required regulatory documents, a supply of Nivolumab and Ipilimumab may be ordered from by completing a Drug Request Form provided by BMS for this specific trial.
- The initial order should be limited to the amount needed for two doses. Allow 5 business days for shipment of drug from BMS receipt of the Drug Request Form. Drug is protocol specific, but not patient specific. All drug products will be shipped by courier in a temperature-controlled container. It is possible that sites may have more than one Nivolumab clinical study ongoing at the same time. <u>It is imperative that only drug product designated for this protocol number be used for this study.</u>
- <u>Pharmacy supplies not provided by BMS:</u> Empty IV bags/containers, approved diluents, In-line filters and infusion tubing

Re-Supply

- Drug re-supply request form should be submitted electronically at least 7 business days before the expected delivery date. Deliveries will be made Tuesday through Friday.
- When assessing need for resupply, institutions should keep in mind the number of vials used per treatment dose, and that shipments may take 14 business days from receipt of request. Drug is not patient-specific. Be sure to check with your pharmacy regarding existing investigational stock to assure optimal use of drug on hand.

Drug Excursions

- Drug excursions should be reported immediately to BMS on the form provided with the study-specific drug order form

8.2 Nivolumab

As this is provided for guidance only, please see investigator brochure for additional information regarding preparation and administration

Nivolumab has a concentration of 10mg/mL and is provided in a 10mL vial. Ten or five vials are provided in a carton.

Storage Conditions & Handling:
- Store at 2-8°C (36-46°F), protect from light, freezing, and shaking.
- If any temperature excursions are encountered during storage, please report these to BMS for assessment via the Temperature Excursion Response Form.
- As with all injectable drugs, care should be taken when handling and preparing Nivolumab. Whenever possible, Nivolumab should be prepared in a laminar flow hood or safety cabinet using standard precautions for the safe handling of intravenous agents applying aseptic technique.
- Partially used vials should be disposed at the site following procedures for the disposal of anticancer drugs.

After final drug reconciliation, unused Nivolumab vials should be disposed at the site following procedures for the disposal of anticancer drugs. For further information, please either discuss with your BMS CSR&O protocol manager or refer to your site IP Destruction policies and procedures

Use Time/Stability: Please refer to the appropriate section of the current Investigator Brochure. Due to parameters surrounding the use time of Nivolumab and Ipilimumab, the time of preparation should be noted in the Pharmacy Source documents [accountability logs] or in study files as required for investigator sponsored research [FDA and GCP]

The administration of BMS-936558-01 injection prepared for dosing Nivolumab infusion must be completed within 24 hours of preparation. If not used immediately, the infusion solution may be stored up to 20 hours in a refrigerator at under refrigeration conditions (2°-°-8°C (, 36°-46°F) and used within 24 hours, and a maximum of 4 hours of the total 24 hours can be at room temperature (20°-25°C, 68°-77°F) and under room light. The maximum 4-hour period under room temperature and room light conditions for undiluted and diluted solutions of BMS-936558-01 injection in the IV bag includes the product administration period.
Nivolumab infusions are to be administered as an IV infusion through a 0.2-micron to 1.2-micron pore size, low-protein binding polyethersulfone membrane in-line filter at the protocol-specified doses. It is not to be administered as an IV push or bolus injection.

Preparation and Administration:

1. Visually inspect the drug product solution for particulate matter and discoloration prior to administration. Discard if solution is cloudy, if there is pronounced discoloration (solution may have a pale-yellow color), or if there is foreign particulate matter other than a few translucent-to-white, amorphous particles. *Note: Mix by gently inverting several times. Do not shake.*
2. Aseptically withdraw the required volume of Nivolumab solution into a syringe, and dispense into an IV. bag. If multiple vials are needed for a subject, it is important to use a separate sterile syringe and needle for each vial to prevent problems such as dulling of needle tip, stopper coring, repeated friction of plunger against syringe barrel wall. **Do not** enter into each vial more than once. **Do not** administer study drug as an IV push or bolus injection
3. Add the appropriate volume of 0.9% Sodium Chloride Injection solution or 5% Dextrose Injection solution. *It is acceptable to add Nivolumab solution from the vials into an appropriate pre-filled bag of diluent.*
4. ***Note: Nivolumab infusion concentration must be at or above the minimum allowable concentration of 0.35 mg/mL [IBV13 Addendum Section 3.2.2]***
5. *Note: It is not recommended that so-called "channel" or tube systems are used to transport prepared infusions of Nivolumab.*
6. Attach the IV bag containing the Nivolumab solution to the infusion set and filter.
7. At the end of the infusion period, flush the line with a sufficient quantity of approved diluents.

8.3 Ipilimumab

Ipilimumab vials (40 mL) are shipped in quantities of four. Ipilimumab (BMS-734016) Injection (5 mg/ml) must be stored refrigerated (2-8°C, 36-46°F) with protection from light and from freezing. Ipilimumab may be stored in IV infusion bags (PVC, non-PVC/non-DEHP) or glass infusion containers for up to 24 hours at room temperature (20-25°C, 68-77°F) or refrigerated (2-8°C, 36-46°F). This would include any time in transit and the total time for infusion. Drug must be completely delivered within 24 hours of preparation.

Storage Conditions & Handling:
Ipilimumab injection may be stored undiluted, 200 mg/vial (5 mg/mL), or following dilution to concentrations between 1 mg/mL and 4 mg/mL in 0.9% Sodium Chloride Injection (USP), or 5% Dextrose Injection (USP) in PVC, non-PVC/ or glass containers for up to 24 hours in the refrigerator (2°C to 8°C) or at room temperature/room light. For longer storage, Ipilimumab should be kept refrigerated (2°C to 8°C) with protection from light.
Ipilimumab injection must not be frozen.
Partially used vials or empty vials of Ipilimumab Injection should be discarded at the site according to appropriate drug disposal procedures.

Preparation and Administration

As this is provided for guidance only, please see investigator brochure for additional information regarding preparation and administration.

1. As Ipilimumab is stored long term at refrigerated temperatures (2-8ºC) and protected from light and freezing, allow the appropriate number of vials of Ipilimumab to stand at room temperature for approximately five minutes.
2. Ensure that the Ipilimumab solution is clear colorless, essentially free from particulate matter on visual inspection. If multiple vials are needed for a subject, it is important to use a separate sterile syringe and needle for each vial to prevent problems such as dulling of needle tip, stopper coring, repeated friction of plunger against syringe barrel wall, etc.
3. Aseptically transfer the required volume of Ipilimumab solution into a syringe. [Note: A sufficient excess of Ipilimumab is incorporated into each vial to account for withdrawal losses].
4. Do not draw into each vial more than once. Discard partially used vials or empty vials.
5. Ipilimumab solution should be added to an appropriate size infusion container to accommodate the calculated final volume.
   Total dose should be calculated using the most recent subject weight; if weight on dosing day differs by 5% from prior weight used to calculate dosing, the dose should be recalculated and study drug adjusted accordingly.
   Mix by GENTLY inverting several times. DO NOT shake.
   Ipilimumab injection may be diluted in 0.9% Sodium Chloride Injection, USP or 5% Dextrose Injection, USP.
6. Visually inspect the final solution. If the initial diluted solution or final solution for infusion is not clear or contents appear to contain precipitate, the solution should be discarded.
7. Immediately after the infusion is complete, flush with an adequate amount of 0.9% Sodium Chloride injection (USP) or 5% Dextrose injection (USP) to completely flush the residual fluid (dead space) in your administration set (approximately 30-50mL); this will ensure that all active drug is delivered to the study participant
8. Safely discard any unused portion of the infusion solution. Do not store for reuse.

Ipilimumab should be administered under the supervision of a physician experienced in the use of intravenous (IV) agents. Ipilimumab is administered as an IV infusion only

Ipilimumab infusions are to be administered as an IV infusion through a 0.2-micron to 1.2-micron pore size, low-protein binding polyethersulfone membrane in-line filter at the protocol-specified doses. It is not to be administered as an IV push or bolus injection.

It is possible that sites may have more than one Ipilimumab clinical study ongoing at the same time. <u>It is imperative that only product designated for this protocol be used for this study.</u>

9. BIOMARKER, CORRELATIVE, AND SPECIAL STUDIES

9.1 Background of exploratory biomarker studies:

The objectives of our exploratory biomarkers are to define the genomic characteristics and the immune microenvironment of leptomeningeal disease in the setting of immune checkpoint inhibitors. Whole-exome sequencing and transcriptome data from non-CNS and CNS metastases and LMD will enable the identification of mutations, insertion/deletion events, and copy-number changes. We also expect to identify genetic signatures that predict response to immunotherapy. Based on our preliminary data across 104 brain metastases, we anticipate that we will identify novel drivers in LMD. We expect to observe changes in RNA/protein expression profiles and identify gene pathways that are consistently and differentially enriched in CSF T cells and myeloid cells by checkpoint blockade. Correlation of clinical outcome with these transcriptomic analyses may lead to predictive signatures of response to immunotherapy. Moreover, we may find evidence of immunosuppression by other pathways, which may suggest new therapeutic strategies for LMD for future investigations. In addition, we may find gene pathways that are enriched in T cells and/or myeloid cells in both the peripheral blood and CSF, which may provide a strategy for immune monitoring of patients on immunotherapy in a more accessible manner. Likewise, we may find changes in cytokines and the proteome in the CSF in response to immunotherapy. If similar cytokines/proteins are altered in the CSF and blood, then such proteins have the potential to serve as biomarkers for responsiveness to immunotherapy.

9.2 Methodology

Whole-exome sequencing: Whole-exome sequencing from tumor and cell-free DNA from CSF and blood will be performed using pipelines that have been tested on several thousand samples at the Broad Institute and described in our recent publications. DNA will be subjected to solution-phase hybrid capture followed by Illumina sequencing to >150x mean coverage. Mutations and allele-specific copy-number changes will be determined within the Firehose pipeline environment at the Broad Institute using a suite of existing tools. The significance of copy-number changes and mutations will be determined using GISTIC and MutSig, respectively, and compared to rates of alterations in the same genes in published large sequencing efforts of unmatched samples. To understand inter- and intratumoral heterogeneity, we will perform an integrative analysis of somatic single nucleotide variants and somatic copy-number alterations. This analysis will allow us to estimate the clonal architecture of the primary and metastatic samples from each patient, and to reconstruct phylogenetic trees relating all of the subclones. For each recurrent mutation, the frequency with which these mutations are present in all or only fractions of cancer cells will be determined.

Transcriptome sequencing and analysis: RNA will be extracted with QIAGEN kits. Non-strand specific RNA sequencing performed at the Broad Institute uses a large-scale, automated variant of the Illumina Tru Seq™ RNA Sample Preparation. This method uses oligo dT beads to select mRNA from the total RNA sample. The selected RNA is heat fragmented and randomly primed before cDNA synthesis from the RNA template. The resultant cDNA then goes through Illumina library prep (end repair, base 'A' addition, adapter ligation, and enrichment) using Broad designed indexed adapters for multiplexing. After enrichment, the samples will be qPCR quantified and equimolar pooled before proceeding to Illumina sequencing, which is done on the Illumina HiSeq 2000. RNA-seq analysis will be carried out using PRADA (Pipeline for RNA-Sequencing Data Analysis). Somatic variant, small deletions and insertion calling will be performed within the

Firehose environment at the Broad Institute with the published caller algorithms.

Cell Purification by Flow Cytometry: CSF will be sorted on CD45 to separate tumor (CD45-) and immune (CD45+) cells for genomic and transcriptomic analyses. $CD4^+$ and $CD8^+$ T cells and myeloid cells ($CD11b^+$) will be purified from the CSF and PBMC by flow cytometry and processed for transcriptome sequencing. We will systematically assess differentially expressed transcripts in pre- and on- treatment samples to determine if there is enrichment within particular gene pathways (such as exhaustion, anergy, senescence) through GeneSet Enrichment Analysis (GSEA) and gene co-expression network analysis.

Cytokine and Proteomic Analyses: We will analyze cytokines in CSF and serum using cytokine bead arrays. High-resolution mass spectrometers and isobaric tandem mass tags will be used to quantify proteins from CSF and serum samples. The tandem mass tag technology allows for the analysis of 6-10 samples within a single experiment. For example, protein expression can be profiled after nine treatment conditions and compared to an untreated sample across >6,000 proteins. All mass spectrometry data will be analyzed in partnership with the Thermo Fisher Scientific Center for Multiplexed Proteomics at Harvard Medical School.

9.3 Collection and Shipping of Tissue Specimen(s)

Collection, Processing and Handling of CSF: One 3 to 10 mL sample of CSF in a clear CSF tube should be collected as per section 10 every three weeks and couriered to the Brastianos laboratory at the address below within 1 hour of obtaining the sample. Label each tube with (1) sample collection date and time, (2) patient study ID and medical record number, (3) protocol study number, and (4) sample type (e.g., CSF). When a sample is shipped, please page the Brastianos lab by calling 617-726-2000 and ask to page Nathaniel Goss at 2-3389 and Brian Shaw at 2-1928.. For any questions or concerns regarding research sample collection or shipment, please contact Nathaniel Goss or Brian Shaw.

Brastianos Lab
185 Cambridge Street
CPZN 3700
Boston, MA 02114

Collection, Processing and Handling of Archived Specimens: If available, for each resection or prior biopsy 1 FFPE block should be clearly labeled and forwarded to Dr. Brastianos' laboratory (at the address below) at Massachusetts General Hospital for molecular testing within 30 days of registration, or surgery happens during trial, within 30 days of surgery or biopsy. If an institution is unable to provide a tissue block, cut 15 unstained slides five-micron sections and mount on charged glass slides. Slides need to be cut with a new blade and using a fresh water bath to avoid contamination. Label the slides with patient ID number, accession number, and order of sections *(include thickness of section if applicable).* For samples containing less than 1 square cm of tumor tissue, multiple sections should be mounted onto each slide to ensure that the appropriate amount of tumor tissue is available. Do not bake or place covers slips on the slide.

Shipping of Tissue Specimen(s): Shipment on Monday through Thursday by overnight service to assure receipt is encouraged. Do not ship specimens on Fridays or Saturdays.

Brittany Bent
Massachusetts General Hospital Cancer Center
55 Fruit Street, Yawkey 9E
Boston, MA 02114

Shipping of Blood Specimen(s): Collect 20 mL of venous blood at pre-registration, 20 mL every 3-4 weeks as per the calendar in section 10. All blood samples should be collected in EDTA anticoagulant vacutainer tubes. The tubes should be inverted approximately 8-10 times to mix the EDTA. Refrigerate (please do not freeze) sample until shipping. The sample should be placed in a biohazard bag and couriered to the Brastianos laboratory at the address below within 1 hour of obtaining the sample. Label each tube with (1) sample collection date and time, (2) patient study ID and medical record number, (3) protocol study number, and (4) sample type (e.g., blood). When a sample is shipped, please page the receiving lab by calling 617-726-2000 and ask to page Nathaniel Goss at 2-3389 and Brian Shaw at 2-1928.For any questions or concerns regarding research sample collection or shipment, please contact Nathaniel Goss or Brian Shaw.

Brastianos Lab
185 Cambridge Street
CPZN 3700
Boston, MA 02114

10. STUDY CALENDAR

Baseline evaluations are to be conducted within 14 days prior to start of protocol therapy, with the exception of CSF with positive cytology, which must be done ≤ 6 weeks prior to initiation of study treatment. Also, if patients received other chemotherapy or targeted therapy within 14 days prior to start of protocol therapy, CBC and serum chemistry (including LFTs and creatinine) should be done within 5 days prior to start of protocol therapy. Scans and x-rays must be done ≤ 4 weeks prior to the start of therapy. In the event that the participant's condition is deteriorating, laboratory evaluations should be repeated within 48 hours prior to initiation of the next cycle of therapy.

Assessments must be performed prior to administration of any study agent. Study assessments and

agents should be administered within ± 3 days of the protocol-specified date, unless otherwise noted.

Calendar During Induction Phase for Melanoma, Small Cell Lung Cancer, Breast Cancer, Bladder Cancer, Renal Cancer and Other Solid Tumors

	Pre-Study	Wk 1	Wk 2	Wk 3	Wk 4	Wk 5	Wk 6	Wk 7	Wk 8	Wk 9	Wk 10	Wk 11	Wk 12	Off Study[h]	Follow-up visits[i]
Ipilimumab		A			A			A			A				
Nivolumab		B			B			B			B				
Informed consent[a]	X														
Demographics[a]	X														
Medical history[a]	X														
Concurrent meds	X	X--X													
Physical exam[b]	X	X	X	X	X	X	X	X	X	X	X	X	X	X	X
Vital signs	X	X	X	X	X	X	X	X	X	X	X	X	X	X	X
Height	X														
Weight	X	X			X			X			X			X	X
Performance status	X	X			X			X			X			X	X
CBC w/diff, plts	X	X			X			X			X			X	X
Serum chemistry[c]	X	X			X			X			X			X	X
EKG (as indicated)[d]	X														
Adverse event evaluation		X--X												X	X
Tumor measurements	X	Tumor measurements are repeated every 6 weeks. Documentation (radiologic) must be provided for participants removed from study for progressive disease.												X	
Radiologic evaluation	X	Radiologic measurements should be performed every 6 weeks (Brain MRI and CT Chest/Abd/Pelvis)												X	X
B-HCG	X[e]							X							X
Cerebrospinal fluid	X[f]	X			X			X			X				
Blood[j]	X	X			X			X			X			X	X
Archival tissue[g]	X													X	X

A: For melanoma, small cell lung cancer, breast cancer, bladder cancer and other solid tumors (except NSCLC and head and neck cancers): Ipilimumab 3mg/kg every 3 weeks x 4 doses during induction.. For renal cell cancer: Ipilimumab 1mg/kg every 3 weeks x 4 doses during induction.

B: For melanoma, small cell lung cancer, breast cancer, bladder cancer and other solid tumors (except NSCLC and head and neck cancers): Nivolumab 1mg/kg every 3 weeks for 4 doses during induction. For renal cell cancer: nivolumab 3mg/kg every 3 weeks x 4 doses during induction.

[a]All screening procedures to be performed within 14 days of start of study treatment, with the exception of CSF with positive cytology, which can be 6 weeks prior to initiation of study treatment. C1D1 and Day 1 of subsequent cycles: For C1D1 only, screening assessments may serve as day 1 assessments, except serum pregnancy (within 72 hours of drug) and except in the event that there are any indications that the participant's condition is deteriorating for which laboratory evaluations should be repeated within 48 hours prior to initiation. For all subsequent cycles, required assessments should be performed within 3 days of scheduled cycle day 1.

[b]Physical examination must include neurologic assessment.

[c]Serum chemistries: Serum chemistry tests include albumin, amylase, lipase, BUN, creatinine, ALT, AST, LDH, serum alkaline phosphatase, direct and total bilirubin, glucose, total protein, sodium, potassium, chloride, HCO3, calcium, uric acid and TSH. A reflexive free T4 and free T3 will be performed reflexively for out of range TSH values. Patients should also be tested for Hepatitis B virus surface antigens (HBV sAg) and hepatitis C virus ribonucleic acid (HCV antibodies) at screening only.

[d]EKG should be obtained at baseline for screening and then as clinically indicated

[e]Serum pregnancy test (women of childbearing potential) is required within 72 hours of C1D1 dose. Pregnancy will be performed every 6 weeks while on treatment, and at 30 days and 70 days post last dose of Niolumab

[f]Cerebrospinal fluid will be collected every 3 weeks during the induction phase and every 4weeks during the maintenance phase to assess disease status (will be sent for cell count, protein, glucose, cytology) and 10cc will be saved for correlative studies as described in Section 9.0

[g]Tissue collected as part of clinical care (e.g. from prior biopsy, craniotomy) will be collected. See Section 9 for specimen collection

[h]: Off-study evaluation: End of treatment assessments to be performed within 7 days after last day drug administration or within 7 days after decision to end treatment. Assessments may continue for ongoing reportable adverse events. In participants who discontinue study treatment without documented disease progression, every effort should be made to continue monitoring their disease status by radiologic imaging every 6 weeks (+/- 7 days) until (1) the start of new

anti-cancer treatment, (2) documented disease progression, (3) death or (4) the end of the study, whichever occurs first
[i]: Follow-up visit 1 = 30 days from the last dose +/- 7 days or coincide with the date of discontinuation (+/- 7 days) if date of discontinuation is greater than 37 days after last dose, Follow-up visit 2 (FU2) = 8-10 weeks (+/- 7 days) from follow-up visit 1. After 2 followup visits, patients will be followed with a phone call every 10-12 weeks for survival information.
[j]Blood will be collected at screening, Day 1, then every 3 weeks during the induction phase and every 4 weeks during the maintenance phase (two 10 mL EDTA/lavender top tube) and at the end of treatment

Calendar During Maintenance Phase for Melanoma, Small Cell Lung Cancer, Breast Cancer, Bladder Cancer, Renal Cancer and other Solid Tumors

	Wk 13	Wk 14	Wk 15	Wk 16	Wk 17	Wk 18	Wk 19	Wk 20	Wk 21	Wk 22	Wk 23	Wk 24	Off Treatment [h]	Follow-up visits[i]
									To be repeated beyond 24 weeks until patient goes off study					
Nivolumab (for melanoma and renal cell carcinoma)	B				B				B					
Nivolumab (for small cell lung cancer, breast cancer and bladder cancer)	C		C		C		C		C		C			
Concurrent meds	X---X													
Physical exam[b]	X		X		X		X		X		X		X	X
Vital signs	X		X		X		X		X		X		X	X
Height														
Weight	X		X		X		X		X		X		X	X
Performance status	X		X		X		X		X		X		X	X
CBC w/diff, plts	X		X		X		X		X		X		X	X
Serum chemistry[c]	X		X		X		X		X		X		X	X
EKG (as indicated)[d]														
Adverse event evaluation	X---X												X	X
Tumor measurements	Tumor measurements are repeated every 8 weeks. Documentation (radiologic) must be provided for participants removed from study for progressive disease.												X	
Radiologic evaluation	Radiologic measurements should be performed every 8 weeks (Brain MRI and CT Chest/Abd/Pelvis)												X	X
B-HCG	X						X							X
Cerebrospinal fluid	X				X				X					
Blood[j]	X				X				X				X	X
Archival tissue[g]													X	X

B: For melanoma, renal cell carcinoma and other solid tumors: Nivolumab 480 mg Q4 weeks during maintenance phase
C: For small cell lung cancer, breast cancer and bladder cancer: nivolumab 240mg every 2 weeks during maintenance phase
[a]All screening procedures to be performed within 14 days of start of study treatment, with the exception of CSF with positive cytology, which can be 6 weeks prior to initiation of study treatment. C1D1 and Day 1 of subsequent cycles: For C1D1 only, screening assessments may serve as day 1 assessments, except serum pregnancy (within 72 hours of drug) and except in the event that there are any indications that the participant's condition is deteriorating for which laboratory evaluations should be repeated within 48 hours prior to initiation. For all subsequent cycles, required assessments should be performed within 3 days of scheduled cycle day 1.
[b]Physical examination must include neurologic assessment.
[c]Serum chemistries: Serum chemistry tests include albumin, amylase, lipase, BUN, creatinine, ALT, AST, LDH, serum alkaline phosphatase, direct and total bilirubin, glucose, total protein, sodium, potassium, chloride, HCO3, calcium, uric acid and TSH. A reflexive free T4 and free T3 will be performed reflexively for out of range TSH values. Patients should also be tested for Hepatitis B virus surface antigens (HBV sAg) and hepatitis C virus ribonucleic acid (HCV antibodies) at screening only.

[d]EKG should be obtained at baseline for screening and then as clinically indicated
[e]Serum pregnancy test (women of childbearing potential) is required within 72 hours of C1D1 dose. Pregnancy will be performed every 6 weeks while on treatment, and at 30 days and 70 days post last dose of Nivolumab.
[f]Cerebrospinal fluid will be collected every 4 weeks during the maintenance phase to assess disease status (will be sent for cell count, protein, glucose, cytology) and 10 cc will be saved for correlative studies as described in Section 9.0
[g]Tissue collected as part of clinical care (e.g. from prior biopsy, craniotomy) will be collected. See Section 9 for specimen collection requirements
h: Off-treatment evaluation: End of treatment assessments to be performed within 7 days after last day drug administration or within 7 days after decision to end treatment. Assessments may continue for ongoing reportable adverse events. In participants who discontinue study treatment without documented disease progression, every effort should be made to continue monitoring their disease status by radiologic imaging every 6 weeks (+/- 7 days) until (1) the start of new anti-cancer treatment, (2) documented disease progression, (3) death or (4) the end of the study, whichever occurs first
[i]: Follow-up visit 1 = 30 days from the last dose +/- 7 days or coincide with the date of discontinuation (+/- 7 days) if date of discontinuation is greater than 37 days after last dose, Follow-up visit 2 (FU2) = 8-10 weeks (+/- 7 days) from follow-up visit 1. After 2 followup visits, patients will be followed with a phone call every 10-12 weeks for survival information.
[j]Blood will be collected at screening, Day 1, then every 3 weeks during the induction phase and every 4 weeks during the maintenance phase (two 10 mL EDTA/lavender top tube) and at the end of treatment

Calendar for Nonsmall Cell Lung Cancer Histology and Head and Neck Cancer

	Pre-Study	Wk 1	Wk 2	Wk 3	Wk 4	Wk 5	Wk 6	Wk 7	Wk 8	Wk 9	Wk 10	Wk 11	Wk 12	Off Study[h]	Follow-up visits[i]
								To be repeated beyond 12 weeks until patient goes off study							
Ipilimumab		A						A							
Nivolumab		B		B		B		B		B		B			
Informed consent[a]	X														
Demographics[a]	X														
Medical history[a]	X														
Concurrent meds	X	X--X													
Physical exam[b]	X	X	X	X	X	X	X	X	X	X	X	X	X	X	X
Vital signs	X	X	X	X	X	X	X	X	X	X	X	X	X	X	X
Height	X														
Weight	X	X		X		X		X		X		X		X	X
Performance status	X	X		X		X		X		X		X		X	X
CBC w/diff, plts	X	X		X		X		X		X		X		X	X
Serum chemistry[c]	X	X		X		X		X		X		X		X	X
EKG (as indicated)[d]	X														
Adverse event evaluation		X--X												X	X
Tumor measurements	X	Tumor measurements are repeated every 6 weeks. Documentation (radiologic) must be provided for participants removed from study for progressive disease.												X	
Radiologic evaluation	X	Radiologic measurements should be performed every 6 weeks (Brain MRI and CT Chest/Abd/Pelvis)												X	X
B-HCG	X[e]							X							X
Cerebrospinal fluid	X[f]	X				X				X					
Blood[j]	X	X				X				X				X	X
Archival tissue[g]	X													X	X
A: Ipilimumab 1mg/kg Q6W															

B: Nivolumab 3mg/kg Q2W

[a]All screening procedures to be performed within 14 days of start of study treatment, with the exception of CSF with positive cytology, which can be 6 weeks prior to initiation of study treatment. C1D1 and Day 1 of subsequent cycles: For C1D1 only, screening assessments may serve as day 1 assessments, except serum pregnancy (within 72 hours of drug) and except in the event that there are any indications that the participant's condition is deteriorating for which laboratory evaluations should be repeated within 48 hours prior to initiation. For all subsequent cycles, required assessments should be performed within 3 days of scheduled cycle day 1.
[b]Physical examination must include neurologic assessment.
[c]Serum chemistries: Serum chemistry tests include albumin, amylase, lipase, BUN, creatinine, ALT, AST, LDH, serum alkaline phosphatase, direct and total bilirubin, glucose, total protein, sodium, potassium, chloride, HCO3, calcium, uric acid and TSH. A reflexive free T4 and free T3 will be performed reflexively for out of range TSH values. Patients should also be tested for Hepatitis B virus surface antigens (HBV sAg) and hepatitis C virus ribonucleic acid (HCV antibodies) at screening only.
[d]EKG should be obtained at baseline for screening and then as clinically indicated
[e]Serum pregnancy test (women of childbearing potential) is required within 72 hours of C1D1 dose. Pregnancy will be performed every 6 weeks while on treatment, and at 30 days and 70 days post last dose of Nivolumab.
[f]Cerebrospinal fluid will be collected every 4 weeks will be sent for cell count, protein, glucose, cytology) and 10cc will be saved for correlative studies as described in Section 9.0
[g]Tissue collected as part of clinical care (e.g. from prior biopsy, craniotomy) will be collected. See Section 9 for specimen collection
[h]: Off-study evaluation: End of treatment assessments to be performed within 7 days after last day drug administration or within 7 days after decision to end treatment. Assessments may continue for ongoing reportable adverse events. In participants who discontinue study treatment without documented disease progression, every effort should be made to continue monitoring their disease status by radiologic imaging every 6 weeks (+/- 7 days) until (1) the start of new anti-cancer treatment, (2) documented disease progression, (3) death or (4) the end of the study, whichever occurs first
[i]: Follow-up visit 1 = 30 days from the last dose +/- 7 days or coincide with the date of discontinuation (+/- 7 days) if date of discontinuation is greater than 37 days after last dose, Follow-up visit 2 (FU2) = 8-10 weeks (+/- 7 days) from follow-up visit 1. After 2 followup visits, patients will be followed with a phone call every 10-12 weeks for survival information.
[j]Blood will be collected at screening, Day 1, then every 4 weeks during the maintenance phase (two 10 mL EDTA/lavender top tube) and at the end of treatment

11. MEASUREMENT OF EFFECT

Response in the CNS and response in non-CNS sites will be evaluated and recorded separately in this trial.

11.1 Antitumor Effect – Extracranial lesion assessments

For the purposes of this study, participants should be re-evaluated for response every 6 weeks. In addition to a baseline scan, confirmatory scans should also be obtained 3 weeks following initial documentation of objective response.

Response and progression will be evaluated in this study using the new international criteria proposed by the Response Evaluation Criteria in Solid Tumors (RECIST) guideline (version 1.1) [*Eur J Ca* 45:228-247, 2009]. Changes in the largest diameter (unidimensional measurement) of the tumor lesions and the shortest diameter in the case of malignant lymph nodes are used in the RECIST criteria.

11.1.1 Definitions

Evaluable for Target Disease response. Only those participants who have leptomeningeal disease present at baseline (as defined by positive cytology), have received at least one cycle of therapy, and have had their disease re-evaluated with restaging scans and cytology will be considered evaluable for target disease response. These participants will have their response classified according to the definitions stated below.

Evaluable Non-Target Disease Response. Participants who have lesions present at baseline that are evaluable but do not meet the definitions of measurable disease, have received at least one cycle of therapy, and have had their disease re-evaluated will be considered evaluable for non-target disease. The response assessment is based on the presence, absence, or unequivocal progression of the lesions.

11.1.2 Disease Parameters

Measurable disease. Measurable lesions are defined as those that can be accurately measured in at least one dimension (longest diameter to be recorded) as ≥ 20 mm by chest x-ray or ≥10 mm with CT scan, MRI, or calipers by clinical exam. All tumor measurements must be recorded in millimeters (or decimal fractions of centimeters).

Note: Tumor lesions that are situated in a previously irradiated area might or might not be considered measurable.

Malignant lymph nodes. To be considered pathologically enlarged and measurable, a lymph node must be ≥15 mm in short axis when assessed by CT scan (CT scan slice thickness recommended to be no greater than 5 mm). At baseline and in follow-up, only the short axis will be measured and followed.

Non-measurable disease. All other lesions (or sites of disease), including small lesions (longest diameter <10 mm or pathological lymph nodes with ≥10 to <15 mm short axis), are considered non-measurable disease. Bone lesions, leptomeningeal disease, ascites, pleural/pericardial effusions, lymphangitis cutis/pulmonitis, inflammatory breast disease, abdominal masses (not followed by CT or MRI), and cystic lesions are all considered non-measurable.

Note: Cystic lesions that meet the criteria for radiographically defined simple cysts should not be considered as malignant lesions (neither measurable nor non-measurable) since they are, by definition, simple cysts.

'Cystic lesions' thought to represent cystic metastases can be considered as measurable lesions, if they meet the definition of measurability described above. However, if non-cystic lesions are present in the same participant, these are preferred for selection as target lesions.

Target lesions. All measurable lesions up to a maximum of 2 lesions per organ and 5 lesions in total, representative of all involved organs, should be identified as **target lesions** and recorded and measured at baseline. Target lesions should be selected on the basis of their size (lesions with the longest diameter), be representative of all involved organs, but in addition should be those that lend themselves to reproducible repeated measurements. It may be the case that, on occasion, the largest lesion does not lend itself to reproducible measurement in which circumstance the next largest lesion which can be measured reproducibly should be selected. A sum of the diameters (longest for non-nodal lesions, short axis for nodal lesions) for all target lesions will be calculated and reported as the baseline sum diameters. If lymph nodes are to be included in the sum, then only the short axis is added into the sum. The baseline sum diameters will be used as reference to further characterize any objective tumor regression in the measurable dimension of the disease.

Non-target lesions. All other lesions (or sites of disease) including any measurable lesions over and above the 5 target lesions should be identified as **non-target lesions** and should also be recorded at baseline. Measurements of these lesions are not required, but the presence, absence, or in rare cases unequivocal progression of each should be noted throughout follow up.

11.1.3 Methods for Evaluation of Disease

All measurements should be taken and recorded in metric notation using a ruler, calipers, or a digital measurement tool. All baseline evaluations should be performed as closely as possible to the beginning of treatment and never more than 4 weeks before the beginning of the treatment.

The same method of assessment and the same technique should be used to characterize each identified and reported lesion at baseline and during follow-up. Imaging-based evaluation is preferred to evaluation by clinical examination unless the lesion(s) being followed cannot be imaged but are assessable by clinical exam.

Clinical lesions. Clinical lesions will only be considered measurable when they are superficial (*e.g.*, skin nodules and palpable lymph nodes) and ≥10 mm in diameter as assessed using calipers (*e.g.*, skin nodules). In the case of skin lesions, documentation by color photography, including a ruler to estimate the size of the lesion, is recommended.

Chest x-ray. Lesions on chest x-ray are acceptable as measurable lesions when they are clearly defined and surrounded by aerated lung; however, CT is preferable.

Conventional CT and MRI. This guideline has defined measurability of lesions on CT scan based on the assumption that CT thickness is 5mm or less. If CT scans have slice thickness greater than 5 mm, the minimum size of a measurable lesion should be twice the slice thickness. MRI is also acceptable in certain situations (*e.g.* for body scans).

Use of MRI remains a complex issue. MRI has excellent contrast, spatial, and temporal resolution; however, there are many image acquisition variables involved in MRI, which greatly impact image quality, lesion conspicuity, and measurement. Furthermore, the availability of MRI is variable globally. As with CT, if an MRI is performed, the technical specifications of the scanning sequences used should be optimized for the evaluation of the type and site of disease. Furthermore, as with CT, the modality used at follow-up should be the same as was used at baseline and the lesions should be measured/assessed on the same pulse sequence. It is beyond the scope of the RECIST guidelines to prescribe specific MRI pulse sequence parameters for all scanners, body parts, and diseases. Ideally, the same type of scanner should be used and the image acquisition protocol should be followed as closely as possible to prior scans. Body scans should be performed with breath-hold scanning techniques, if possible.

FDG-PET. While FDG-PET response assessments need additional study, it is sometimes reasonable to incorporate the use of FDG-PET scanning to complement CT scanning in assessment of progression (particularly possible 'new' disease). New lesions on the basis of FDG-PET imaging can be identified according to the following algorithm:

(a) Negative FDG-PET at baseline, with a positive FDG-PET at follow-up is a sign of PD based on a new lesion.

(b) No FDG-PET at baseline and a positive FDG-PET at follow-up: If the positive FDG-PET at follow-up corresponds to a new site of disease confirmed by CT, this is PD. If the positive FDG-PET at follow-up is not confirmed as a new site of disease on CT, additional follow-up CT scans are needed to determine if there is truly progression occurring at that site (if so, the date of PD will be the date of the initial abnormal FDG-PET scan). If the positive FDG-PET at follow-up corresponds to a pre-existing site of disease on CT that is not progressing on the basis of the anatomic images, this is not PD.

(c) FDG-PET may be used to upgrade a response to a CR in a manner similar to a biopsy in cases where a residual radiographic abnormality is thought to represent fibrosis or scarring. The use of FDG-PET in this circumstance should be prospectively described in the protocol and supported by disease-specific medical literature for the indication. However, it must be acknowledged that both approaches may lead to false

positive CR due to limitations of FDG-PET and biopsy resolution/sensitivity.

Note: A ‘positive’ FDG-PET scan lesion means one which is FDG avid with an uptake greater than twice that of the surrounding tissue on the attenuation corrected image.

PET-CT. At present, the low dose or attenuation correction CT portion of a combined PET-CT is not always of optimal diagnostic CT quality for use with RECIST measurements. However, if the site can document that the CT performed as part of a PET-CT is of identical diagnostic quality to a diagnostic CT (with IV and oral contrast), then the CT portion of the PET-CT can be used for RECIST measurements and can be used interchangeably with conventional CT in accurately measuring cancer lesions over time. Note, however, that the PET portion of the CT introduces additional data which may bias an investigator if it is not routinely or serially performed.

Cytology, Histology. These techniques can be used to differentiate between partial responses (PR) and complete responses (CR) in rare cases (*e.g.*, residual lesions in tumor types, such as germ cell tumors, where known residual benign tumors can remain).

The cytological confirmation of the neoplastic origin of any effusion that appears or worsens during treatment when the measurable tumor has met criteria for response or stable disease is mandatory to differentiate between response or stable disease (an effusion may be a side effect of the treatment) and progressive disease.

11.1.4 Response Criteria

11.1.4.1 Evaluation of Target Lesions

Complete Response (CR): Disappearance of all target lesions. Any pathological lymph nodes (whether target or non-target) must have reduction in short axis to <10 mm.

Partial Response (PR): At least a 30% decrease in the sum of the diameters of target lesions, taking as reference the baseline sum diameters.

Progressive Disease (PD): At least a 20% increase in the sum of the diameters of target lesions, taking as reference the smallest sum on study (this includes the baseline sum if that is the smallest on study). In addition to the relative increase of 20%, the sum must also demonstrate an absolute increase of at least 5 mm. (Note: the appearance of one or more new lesions is also considered progressions).

Stable Disease (SD): Neither sufficient shrinkage to qualify for PR nor sufficient increase to qualify for PD, taking as reference the smallest sum diameters while on study.

11.1.4.2 Evaluation of Non-Target Lesions

Complete Response (CR): Disappearance of all non-target lesions and normalization

of tumor marker level. All lymph nodes must be non-pathological in size (<10 mm short axis).

Note: If tumor markers are initially above the upper normal limit, they must normalize for a patient to be considered in complete clinical response.

Non-CR/Non-PD: Persistence of one or more non-target lesion(s) and/or maintenance of tumor marker level above the normal limits.

Progressive Disease (PD): Appearance of one or more new lesions and/or *unequivocal progression* of existing non-target lesions. *Unequivocal progression* should not normally trump target lesion status. It must be representative of overall disease status change, not a single lesion increase.

Although a clear progression of "non-target" lesions only is exceptional, the opinion of the treating physician should prevail in such circumstances, and the progression status should be confirmed at a later time by the review panel (or Principal Investigator).

11.1.4.3 Evaluation of New Lesions

The finding of a new lesion should be unequivocal (i.e. not due to difference in scanning technique, imaging modality, or findings thought to represent something other than tumor (for example, some 'new' bone lesions may be simply healing or flare of pre-existing lesions). However, a lesion identified on a follow-up scan in an anatomical location that was not scanned at baseline is considered new and will indicate PD. If a new lesion is equivocal (because of small size etc.), follow-up evaluation will clarify if it truly represents new disease and if PD is confirmed, progression should be declared using the date of the initial scan on which the lesion was discovered.

11.1.4.4 Evaluation of Best Overall Response

The best overall response is the best response recorded from the start of the treatment until disease progression/recurrence (taking as reference for progressive disease the smallest measurements recorded since the treatment started). The patient's best response assignment will depend on the achievement of both measurement and confirmation criteria.

For Participants with Measurable Disease (*i.e.*, Target Disease)

Target Lesions	Non-Target Lesions	New Lesions	Overall Response	Best Overall Response when Confirmation is Required*
CR	CR	No	CR	≥4 wks Confirmation**
CR	Non-CR/Non-PD	No	PR	≥4 wks Confirmation**
CR	Not evaluated	No	PR	
PR	Non-CR/Non-	No	PR	

	PD/not evaluated			
SD	Non-CR/Non-PD/not evaluated	No	SD	Documented at least once ≥4 wks from baseline**
PD	Any	Yes or No	PD	no prior SD, PR or CR
Any	PD***	Yes or No	PD	
Any	Any	Yes	PD	

* See RECIST 1.1 manuscript for further details on what is evidence of a new lesion.
** Only for non-randomized trials with response as primary endpoint.
*** In exceptional circumstances, unequivocal progression in non-target lesions may be accepted as disease progression.

Note: Participants with a global deterioration of health status requiring discontinuation of treatment without objective evidence of disease progression at that time should be reported as "*symptomatic deterioration.*" Every effort should be made to document the objective progression even after discontinuation of treatment.

For Participants with Non-Measurable Disease (*i.e.*, Non-Target Disease)

Non-Target Lesions	New Lesions	Overall Response
CR	No	CR
Non-CR/non-PD	No	Non-CR/non-PD*
Not all evaluated	No	not evaluated
Unequivocal PD	Yes or No	PD
Any	Yes	PD

* 'Non-CR/non-PD' is preferred over 'stable disease' for non-target disease since SD is increasingly used as an endpoint for assessment of efficacy in some trials so to assign this category when no lesions can be measured is not advised

11.1.5 Duration of Response

Duration of overall response: The duration of overall response is measured from the time measurement criteria are met for CR or PR (whichever is first recorded) until the first date that recurrent or progressive disease is objectively documented (taking as reference for progressive disease the smallest measurements recorded since the treatment started, or death due to any cause. Participants without events reported are censored at the last disease evaluation).

Duration of overall complete response: The duration of overall CR is measured from the time measurement criteria are first met for CR until the first date that progressive disease is objectively documented, or death due to any cause. Participants without events reported are censored at the last disease evaluation.

Duration of stable disease: Stable disease is measured from the start of the treatment until the criteria for progression are met, taking as reference the smallest measurements recorded since the treatment started, including the baseline measurements.

11.1.6 Progression-Free Survival

Overall Survival: Overall Survival (OS) is defined as the time from registration to death due to any cause, or censored at date last known alive.

Progression-Free Survival: Progression-Free Survival (PFS) is defined as the time from registration to the earlier of progression or death due to any cause. Participants alive without disease progression are censored at date of last disease evaluation.

Time to Progression: Time to Progression (TTP) is defined as the time from randomization (or registration) to progression, or censored at date of last disease evaluation for those without progression reported.

11.2 Antitumor Effect – Intracranial lesion assessments

For the purposes of this study, participants should be re-evaluated for intracranial response every 6 weeks. In addition to a baseline scan, confirmatory scans should also be obtained at 4 weeks following initial documentation of objective response. Response and progression will be evaluated in this study using the modified RANO Response Criteria.

11.2.1 Definitions

Evaluable for objective response. Only those participants who have measurable CNS disease present at baseline, have received at least one cycle of therapy, and have had their disease re-evaluated will be considered evaluable for target disease response in the CNS. These participants will have their response classified according to the definitions stated below. (Note: Participants who exhibit objective disease progression prior to the end of cycle 1 will also be considered evaluable.)

Evaluable non-measurable evaluable disease. Participants who have lesions present at baseline that are evaluable but do not meet the definitions of measurable disease, have received at least one cycle of therapy, and have had their disease re-evaluated will be considered evaluable for non-target disease. The response assessment is based on the presence, absence, or unequivocal progression of the lesions.

11.2.2 Disease Parameters

Measurable disease. Measurable lesions are defined as those that can be accurately measured in at least one dimension (longest diameter to be recorded) as ≥10 mm with CT scan, MRI, or calipers by clinical exam. All tumor measurements must be recorded in millimeters (or decimal fractions of centimeters).

For multifocal intracranial disease, no more than 5 target measurable lesions (each ≥10mm in diameter in both dimensions) should be selected for measurement. Target lesions should be selected on the basis of their size (lesions with longest diameter), be representative of other lesions and lend themselves to reproducible repeated measurements.

Non-measurable disease. Unidimensionally measurable lesions, masses with margins not clearly defined, lesions with maximal diameter < 1cm.

11.2.3 Methods for Evaluation of Disease

All measurements should be taken and recorded in metric notation using a ruler, calipers, or a digital measurement tool. All baseline evaluations should be performed as closely as possible to the beginning of treatment and never more than 4 weeks before the beginning of the treatment.

The same method of assessment and the same technique should be used to characterize each identified and reported lesion at baseline and during follow-up. Imaging-based evaluation is preferred to evaluation by clinical examination unless the lesion(s) being followed cannot be imaged but are assessable by clinical exam.

Conventional head CT and brain MRI. This guideline has defined measurability of lesions on CT scan based on the assumption that CT thickness is 5mm or less. If CT scans have slice thickness greater than 5 mm, the minimum size of a measurable lesion should be twice the slice thickness.

11.2.4 Response Criteria

11.2.4.1 Evaluation of Intracranial Lesions

Complete Response (CR): Requires all of the following:

- complete disappearance of all enhancing measurable and nonmeasurable disease sustained for at least 4 weeks
- no new lesions
- stable or decreasing corticosteroids (the steroid dose at the time of the scan evaluation should be no greater than the dose at time of baseline scan)
- stable or improved clinically

Participants with non-measurable disease cannot have a complete response. The best possible response is stable disease.

Partial Response (PR): All of the following criteria must be met:

- Greater than or equal to 50% decrease compared to baseline in the sum of products of perpendicular diameters of all measurable enhancing lesions sustained for at least 4 weeks. In the absence of a confirming scan 4 weeks later,

this scan will be considered only stable disease.

- no progression of non-measurable disease.
- no new lesions
- stable or reduced corticosteroid dose (the steroid dose at the time of the scan evaluation should be no greater than the dose at time of baseline scan)
- stable or improved clinically

Progressive Disease (PD): At least one of the following must be true:

- ≥ 25% increase in the sum of products of all measurable lesions over smallest sum observed (over baseline if no decrease) using the same techniques as baseline
- clear worsening of any nonmeasurable disease*
- appearance of any new enhancing lesion/site
- clear clinical worsening (unless clearly unrelated to this cancer, e.g. seizures, medication side effects, complications of therapy, cerebrovascular events, infecion)
- failure to return for evaluation due to death or deteriorating condition

* Progression of non-measurable CNS lesions is defined as follows:

- a lesion initially at baseline </=5mm in diameter that increases to >/= 10mm in diameter, or
- a lesion initially at baseline 6-9 mm in diameter that increases by at least 5 mm in diameter.

Stable Disease (SD): All of the following criteria must be met:

- Does not qualify for CR, PR or progression
- All measurable and non-measurable sites must be assessed using the same techniques as baseline
- Stable clinically.

A summary of the response criteria is in the below:

	Complete Response	Partial Response	Stable Disease	Progressive Disease
T1-Gd+	None	≥ 50% decrease	<50% decrease- <25% increase	≥25% increase*
New Lesion	None	None	None	Present*
Corticosteroids	Stable or decrease	Stable or decrease	Stable or decrease	Stable or increasing
Clinical Status	Stable or improved	Stable or improved	Stable or improved	Decrease*
Requirement for Response	All	All	All	Any*

NA=not applicable<br>#: Progression occurs when any of the criteria with * is present<br>Increase in corticosteroids alone will not be taken into account in determining progression in the absence of persistent clinical deterioriation

11.2.4.2 Evaluation of Best Overall Response

The best overall response is the best response recorded from the start of the treatment until disease progression/recurrence (taking as reference for progressive disease the smallest measurements recorded since the treatment started). The patient's best response assignment will depend on the achievement of both measurement and confirmation criteria. If a response recorded at one scheduled MRI does not persist at the next regular scheduled MRI, the response will still be recorded based on the prior scan, but will be designated as a non-sustained response. If the response is sustained, i.e. still present on the subsequent MRI at least four weeks later, it will be recorded as a sustained response, lasting until the time of tumor progression. Participants without measurable disease may only achieve SD or PD as their best "response."

11.2.4.3 Duration of Response

Duration of overall response: The duration of overall response is measured from the time measurement criteria are met for CR or PR (whichever is first recorded) until the first date that recurrent or progressive disease is objectively documented (taking as reference for progressive disease the smallest measurements recorded since the treatment started, or death due to any cause. Participants without events reported are censored at the last disease evaluation).

Duration of overall complete response: The duration of overall CR is measured from the time measurement criteria are first met for CR until the first date that progressive disease is objectively documented, or death due to any cause. Participants without events reported are censored at the last disease evaluation.
Duration of stable disease: Stable disease is measured from the start of the treatment until the criteria for progression are met, taking as reference the smallest measurements recorded since the treatment started, including the baseline measurements.

11.2.5 Study Continuation Beyond Initial Progressive Disease

Immunotherapeutic agents such as ipilimumab and nivolumab may produce antitumor effects by potentiating endogenous cancer-specific immune responses which may manifest as initial worsening of enhancement and edema on MRI or CT scans (i.e. pseudo-progression). In addition, the response patterns seen with immunotherapeutics may extend beyond the typical time course of responses seen with cytotoxic agents, and can manifest a clinical response after an initial increase in tumor burden or even the appearance of new lesions. For these reasons, the immune- related response criteria (irRC) have endorsed continuation of study therapy beyond initial radiographic evidence of progression for

clinically stable patients undergoing immune based therapies.

Therefore, the following adaptations will be used to assess response for patients treated on this study:

Potential Pseudo-progression: If radiologic imaging shows initial PD, subjects who are not experiencing significant clinical decline, may be allowed to continue study treatment for up to three months. Patients who have radiographic evidence of further progression after up to three months, or who decline significantly at anytime, will be classified as progressive with the date of disease progression back-dated to the first date that the subject met criteria for progression and such subjects will be discontinued from study therapy. Three months is the most common timeframe for pseudo-progression observed among patients with advanced melanoma or other solid tumors treated with PD-1/PD-L1 immune checkpoint blockade[17].

Among patients on this study with initial radiographic PD, tumor assessment should be repeated regularly in order to confirm PD with the option of continuing treatment as described below while awaiting radiologic confirmation of progression. If repeat imaging shows a stabilization or reduction in the tumor burden compared to the initial scan demonstrating PD, treatment may be continued / resumed. If repeat imaging after up to three months confirms progressive disease, then the date of disease progression will be the first date the subject met criteria for progression and subjects will be discontinued from study therapy. Subjects who have confirmed disease progression will discontinue study medication and enter the follow up/survival phase of the study. In determining whether or not the tumor burden has increased or decreased, investigators should consider all target lesions as well as non-target lesions.

Tumor Enhancement to Define Progression: RANO expanded the previously utilized Macdonald criteria[18] to include the development of "significantly" increased T2 or FLAIR abnormality in the definition of progressive disease because such changes can be a major component defining radiographic progression following therapeutic use of VEGF/VEGFR-targeting therapeutics which are known to elicit potent anti-permeability changes that limit contrast uptake. However, immune based therapies are expected to be associated with inflammatory changes that may include edema. Therefore, our study will define radiographic progressive disease by assessment of enhancing tumor burden only and will not include assessment of T2 or FLAIR changes as outlined in RANO because:

- There is no expectation that immunotherapy agents including PD-1 inhibitors will falsely diminish enhancing tumor burden as has been noted with anti-angiogenic therapies; and
- Immune based therapies are expected to induce inflammatory responses which may be associated with increased edema and T2/FLAIR changes. Such radiographic finding may inaccurately be interpreted to represent tumor progression (i.e. pseudoprogression).

In subjects who have initial evidence of radiographic PD, it is at the discretion of the

treating physician whether to continue a subject on study treatment for up to three months pending confirmation of PD on follow-up imaging. This clinical judgment decision should be based on the subject's overall clinical condition, including performance status, clinical symptoms, and laboratory data. Subjects may receive study treatment while waiting for confirmation of PD if they are not experiencing significant clinical decline and if:

- The subject is believed to demonstrate clinical benefit from the study regimen as determined by the treating physician;
- The subject is adequately tolerating study therapy.

When feasible, study therapy should not be discontinued until radiographic progression is confirmed. This allowance to continue treatment despite initial radiologic progression takes into account the observation that some subjects can have a transient tumor flare in the first few months after the start of immunotherapy, but with subsequent disease response[19]. Subjects that are exhibiting significant neurologic decline are not required to have repeat imaging for confirmation of progressive disease.

Participants with progressive radiographic findings are encouraged to undergo surgical intervention in order to delineate pseudo-progression due to inflammation associated with PD-1 blockade from true tumor progression. Participants with histopathologic findings of significant immune infiltrate and evolving gliosis will be allowed to continue study therapy. In contrast, those with clear evidence of progressive tumor by histopathologic evaluation will be defined as progressive and discontinued from study therapy. For such patients, the date of tumor progression will be the first date the participant met radiographic criteria for PD.

11.3 Response Review

All brain MRI scans will be reviewed centrally by the DF/HCC Tumor Imaging Metrics Core (TIMC). CNS response will be assessed centrally using the RANO criteria outlined in Section 12.1.4.

All non-CNS scans will be reviewed centrally by the DF/HCC Tumor Imaging Metrics Core (TIMC). Extracranial response will be assessed centrally using RECIST criteria.

11.4 Other Response Parameters

<u>Cerebrospinal Fluid:</u> Cerebrospinal fluid will collected as part of clinical care. Leptomeningeal response will be defined as below:

Response	Cytologic	Neurologic	Radiographic
Complete Response	Reversion to (-) CSF cytology (or NA)	Disappearance of all disease related symptoms present prior to therapy (or NA)	Complete disappearance of all detectable radiographic EOD (or NA)

Partial Response	> 50% decrease in malignant CSF cytology	Improvement in neurologic symptoms or no worsening of symptoms	Improvement in radiographic evidence of disease. No new sites of measurable or assessable CNS disease
Stable Disease	Not fulfilling the criteria for CR or PR, but not progressive disease	Not fulfilling the criteria for CR or PR, but not progressive disease	Not fulfilling the criteria for CR, or PR but is not progressive disease
Progressive Disease	> 50% increase in malignant CSF cytology	Development of new neurologic symptoms related to neoplastic meningitis	Increase in radiographic evidence of disease. Appearance of new sites of disease.

Leptomeningeal response will be a secondary outcome.

The immune related Response Criteria (irRC) will also be used in this study to support secondary efficacy endpoints for extracranial lesiona. Definitions of the immune-related Response Criteria (irRC) are below and are presented in Appendix B:

- Complete Response (CR): Disappearance of all lesions in two consecutive observations not less than 4 wk apart
- Partial Response (PR): ≥50% decrease in tumor burden compared with baseline in two observations at least 4 wk apart
- Stable Disease (SD): 50% decrease in tumor burden compared with baseline cannot be established nor 25% increase compared with nadir
- Progressive Disease (PD): At least 25% increase in tumor burden compared with nadir (at any single time point) in two consecutive observations at least 4 wk apart

Index Lesion	Non-Index Lesion	New Measurable Lesions	New unmeasurable lesions	% change in tumor burden	Overall IR Response
CR	CR	No	No	-100%	irCR
PR	Any	Any	Any	≥ -50%	irPR
PR	Any	Any	Any	< -50% to <25%	irSD
PR	Any	Any	Any	≥ +25%	irPD
SD	Any	Any	Any	< -50% to <+25%	irSD
SD	Any	Any	Any	≥ +25%	irPD
PD	Any	Any	Any	≥ +25%	irPD

12. DATA REPORTING / REGULATORY REQUIREMENTS

Adverse event lists, guidelines, and instructions for AE reporting can be found in Section 7.0 (Adverse Events: List and Reporting Requirements).

12.1 Data Reporting

12.1.1 Method

The ODQ will collect, manage, and perform quality checks on the data for this study.

12.1.2 Responsibility for Data Submission

Investigative sites within DF/HCC or DF/PCC are responsible for submitting data and/or data forms to the ODQ according to the schedule set by the ODQ.

Form	Submission Timeline
Eligibility Checklist	Complete prior to registration with ODQ
On Study Form	Within 14 days of registration
Baseline Assessment Form	Within 14 days of registration
Treatment Form	Within 10 days of treatment administration
Adverse Event Report Form	Within 10 days of AE assessment/notification
Response Assessment Form	Within 10 days of response assessment
Off Treatment/Off Study Form	Within 14 days of completing treatment or being taken off study for any reason
Follow up/Survival Form	Within 14 days of the protocol defined follow up visit date or call

12.2 Data Safety Monitoring

The DF/HCC Data and Safety Monitoring Committee (DSMC) will review and monitor toxicity and accrual data from this study. The committee is composed of clinical specialists with experience in oncology and who have no direct relationship with the study. Information that raises any questions about participant safety will be addressed with the Overall PI and study team.

The DSMC will review each protocol up to four times a year or more often if required to review toxicity and accrual data. Information to be provided to the committee may include: up-to-date participant accrual; current dose level information; DLT information; all grade 2 or higher unexpected adverse events that have been reported; summary of all deaths occurring with 30 days of intervention for Phase I or II protocols; for gene therapy protocols, summary of all deaths while being treated and during active follow-up; any response information; audit results, and a summary provided by the study team. Other information (e.g. scans, laboratory values) will be provided upon request.

12.3 Multicenter Guidelines

N/A

12.4 Collaborative Agreements Language

N/A

13. STATISTICAL CONSIDERATIONS

This is a single arm phase II trial to investigate the therapeutic effect of ipilimumab and nivolumab in leptomeningeal carcinomatosis.

13.1 Study Design/Endpoints

Primary endpoint: Rate of overall survival at 3 months

Secondary Endpoints:
1. Toxicity by CTCAE criteria (grade 3 or higher hematologic toxicity; grade 3 or higher neurologic toxicity)
2. Intracranial response in patients with leptomeningeal metastases
3. Extracranial response as determined by RECIST and irRC
4. Leptomeningeal disease response as defined in section 11.4
5. Time to the first occurrence of extracranial disease progression, or death from any cause
6. Time to the first occurrence of intracranial disease progression, or death from any cause

Exploratory Endpoints: Genetic alterations

13.2 Sample Size, Accrual Rate and Study Duration

Accrual of up to 20 patients may be required to achieve 18 evaluable patients.

The primary endpoint is the rate of overall survival at 3 months (OS3). Any patient whose vital status at 3 months cannot be determined will be counted as having died. A Simon two-stage design will be used to compare a null hypothesis OS3 of 18% against an alternative of 43%. Nine patients will be enrolled in the first stage. If 0 or 1 patients are alive at 3 months, the trial will be stopped early. If 2 or more patients are alive at 3 months, we will enroll an additional 9 patients. If at least 6 patients among the total of 18 patients are alive at 3 months, then the treatment would be promising in the cohort. This design has a type-I error of 8% (target 10%) and power of 86% (target 85%). If the null hypothesis is true, then the probability is 0.50 of stopping at the end of the first stage.

In addition, we will assess for the primary outcome of any histologic, molecular subtype (e.g., ER/PR-positive, HER2-positive breast cancer, EGFR mutant lung cancer) with 6 patients enrolled for that particular subtype. If no patient within a particular histologic subtype has achieved the primary outcome, then patients with that histology will no longer be eligible to enroll in the trial. Under the alternative hypothesis (i.e., OS3 = 43%), the probability is 0.03 of observing no patients with OS3 among 6 patients.

The accrual rate is based on our institutional patient volume of 3-4 patients per month with leptomeningeal carcinomatosis seen in our multidisciplinary brain metastasis clinic. We estimate that 1-2 patients per month will be eligible for this protocol. Therefore, 20 patients will be accrued within 10-20 months. Based on six months of follow-up (median survival of patients is approximately 2-4 months), total study duration will be 24 months.

13.3 Stratification Factors

N/A

13.4 Interim Monitoring Plan

N/A

13.5 Analysis of Primary Endpoints

The primary endpoint is the rate of overall survival at 3 months (OS3). Any patient whose vital status is unknown due to loss of follow-up will be classified as having died for purposes of estimating the primary endpoint. The proportion of patients alive at 3 months will be summarized with a 90% confidence interval estimated using the method of Atkinson and Brown, which allows for the two-stage design. Based on a sample of 18 patients, the confidence interval will be no wider than 0.42.

13.6 Analysis of Secondary Endpoints

Safety and Tolerability: All adverse events recorded during the trial will be summarized for all patients having received one or more doses of ipilimumab and nivolumab. The data for each cohort will be summarized separately, although safety data may be combined, if appropriate. The proportions of patients with grade-3 or higher hematologic toxicities or grade-3 or higher neurologic toxicities will be presented with 90% exact binomial confidence intervals. The incidence of events that are new or worsening from the time of first dose of treatment will be summarized according to system organ class and/or preferred term, severity (based on CTCAE grade), type of adverse event, and relation to study treatment. Deaths reportable as SAEs and non-fatal serious adverse events will be listed by patient and tabulated by primary system organ class, and type of adverse event.

Intracranial/Extracranial Response: The proportion of patients with intracranial response of intraparenchymal brain metastases (CR or PR by RANO) will be presented with a two-sided, 90% exact binomial confidence interval. For a sample of 18 patients, the confidence interval will have maximum width of 0.42. Extracranial response rates (CR or PR) according to RECIST and irRC will be summarized. Rates will be accompanied by two-sided 90% exact binomial confidence intervals. Cases where RECIST and irRC responses do not agree will be documented.

Leptomeningeal Disease Response: The proportion of patients with leptomeningeal disease response (CR or PR) will be presented with a two-sided, 90% exact binomial confidence interval.

For a sample of 18 patients, the confidence interval will have maximum width of 0.42.

Extracranial PFS and Intracranial PFS: Extracranial PFS (EPFS) is defined as the time from first dose of study drug to documented extracranial progression (per RECIST or irRC) or death, whichever occurs first. Intracranial PFS (IPFS) is defined as the time from first dose of ipilimumab and nivolumab to documented intracranial progression (RANO) or death, whichever occurs first. For both endpoints, the follow-up of patients who have neither died nor progressed at the time of analysis will be censored at the date of last adequate disease assessment. Each cohort will be presented separately. Median EPFS and IPFS will be presented and accompanied by 90% confidence intervals estimated using log(-log(survival)) methodology. Point estimates at 3 and 6 months will also be presented with confidence intervals.

13.7 Analysis of Exploratory Endpoints

Biomarkers of blood and tissue: Analyses of blood and tissue biomarkers will be based on descriptive statistics, as appropriate for the biomarker.

To examine response according to pre-treatment levels of biomarkers, the study sample will be divided retrospectively according to the primary endpoint of response or non-response. Pre-treatment biomarker levels will be summarized descriptively for the two response groups and compared using Wilcoxon rank-sum tests for markers measured on a continuous scale, or Fisher's exact tests for those measured on a categorical scale. Where appropriate, visualization of the relationship between baseline marker levels and the distributions of PFS or OS will employ Kaplan-Meier estimates stratified by biomarker levels. Medians of the time-to-event endpoints will be shown with two-sided 90% confidence intervals; the distributions of PFS and OS will be compared across biomarker strata using the log-rank test.

Changes in biomarkers between pretreatment and progression or treatment discontinuation will be calculated (post-pre) for each patient and summarized descriptively. In addition, the correlation of changes in tissue biomarkers with changes in measures of peripheral blood markers will be explored graphically, or by appropriate statistical methods based on data availability, to assess associations.

13.8 Reporting and Exclusions

13.8.1 Evaluation of Toxicity

All participants who have received at least one dose of nivolumab and ipilimumab will be evaluable for toxicity from the time of their first treatment.

13.8.2 Evaluation of the Primary Efficacy Endpoint

To be included in the assessment of the primary objective, patients need to receive at least one cycle.

14. PUBLICATION PLAN

The results should be made public within 24 months of reaching the end of the study. The end of the study is the time point at which the last data items are to be reported, or after the outcome data are sufficiently mature for analysis, as defined in the section on Sample Size, Accrual Rate and Study Duration. If a report is planned to be published in a peer-reviewed journal, then that initial release may be an abstract that meets the requirements of the International Committee of Medical Journal Editors. A full report of the outcomes should be made public no later than three (3) years after the end of the study.

REFERENCES

1. Brastianos PK, Brastianos H, Eichler AF. Leptomeningeal Carcinomatosis. In: Palmieri D, ed. Central Nervous System Metastasis, the Biological Basis and Clinical Consideraiton. New York: Springer; 2013:187-201.
2. Raizer JJ, Hwu WJ, Panageas KS, et al. Brain and leptomeningeal metastases from cutaneous melanoma: survival outcomes based on clinical features. Neuro-oncology 2008;10:199-207.
3. Glantz MJ, Jaeckle KA, Chamberlain MC, et al. A randomized controlled trial comparing intrathecal sustained-release cytarabine (DepoCyt) to intrathecal methotrexate in patients with neoplastic meningitis from solid tumors. Clin Cancer Res 1999;5:3394-402.
4. Grossman SA, Finkelstein DM, Ruckdeschel JC, Trump DL, Moynihan T, Ettinger DS. Randomized prospective comparison of intraventricular methotrexate and thiotepa in patients with previously untreated neoplastic meningitis. Eastern Cooperative Oncology Group. J Clin Oncol 1993;11:561-9.
5. Chapman PB, Hauschild A, Robert C, et al. Improved survival with vemurafenib in melanoma with BRAF V600E mutation. N Engl J Med 2011;364:2507-16.
6. Flaherty KT, Robert C, Hersey P, et al. Improved Survival with MEK Inhibition in BRAF-Mutated Melanoma. N Engl J Med 2012.
7. Hauschild A, Grob JJ, Demidov LV, et al. Dabrafenib in BRAF-mutated metastatic melanoma: a multicentre, open-label, phase 3 randomised controlled trial. Lancet 2012;380:358-65.
8. Hodi FS, O'Day SJ, McDermott DF, et al. Improved survival with ipilimumab in patients with metastatic melanoma. N Engl J Med 2010;363:711-23.
9. Robert C, Ribas A, Wolchok JD, et al. Anti-programmed-death-receptor-1 treatment with pembrolizumab in ipilimumab-refractory advanced melanoma: a randomised dose-comparison cohort of a phase 1 trial. Lancet 2014;384:1109-17.
10. Robert C, Thomas L, Bondarenko I, et al. Ipilimumab plus dacarbazine for previously untreated metastatic melanoma. N Engl J Med 2011;364:2517-26.
11. Wolchok JD, Kluger H, Callahan MK, et al. Nivolumab plus Ipilimumab in Advanced Melanoma. N Engl J Med 2013.
12. Long GV, Trefzer U, Davies MA, et al. Dabrafenib in patients with Val600Glu or Val600Lys BRAF-mutant melanoma metastatic to the brain (BREAK-MB): a multicentre, open-label, phase 2 trial. Lancet Oncol 2012.
13. Margolin K, Ernstoff MS, Hamid O, et al. Ipilimumab in patients with melanoma and brain metastases: an open-label, phase 2 trial. Lancet Oncol 2012;13:459-65.
14. Margolin K, Ernstoff MS, Hamid O, et al. Ipilimumab in patients with melanoma and brain metastases: an open-label, phase 2 trial. Lancet Oncol 2012;13:459-65.
15. Ahmed KA, Stallworth DG, Kim Y, et al. Clinical outcomes of melanoma brain metastases treated with stereotactic radiation and anti-PD-1 therapy. Ann Oncol 2016;27:434-41.
16. Bot I, Blank CU, Brandsma D. Clinical and radiological response of leptomeningeal melanoma after whole brain radiotherapy and ipilimumab. J Neurol 2012;259:1976-8.
17. Brahmer JR, Tykodi SS, Chow LQ, et al. Safety and activity of anti-PD-L1 antibody in patients with advanced cancer. N Engl J Med 2012;366:2455-65.
18. Macdonald DR, Cascino TL, Schold SC, Jr., Cairncross JG. Response criteria for

phase II studies of supratentorial malignant glioma. J Clin Oncol 1990;8:1277-80.
19. Wolchok JD, Hoos A, O'Day S, et al. Guidelines for the evaluation of immune therapy activity in solid tumors: immune-related response criteria. Clin Cancer Res 2009;15:7412-20.

APPENDIX A PERFORMANCE STATUS CRITERIA

ECOG Performance Status Scale		Karnofsky Performance Scale	
Grade	Descriptions	Percent	Description
0	Normal activity. Fully active, able to carry on all pre-disease performance without restriction.	100	Normal, no complaints, no evidence of disease.
		90	Able to carry on normal activity; minor signs or symptoms of disease.
1	Symptoms, but ambulatory. Restricted in physically strenuous activity, but ambulatory and able to carry out work of a light or sedentary nature (*e.g.*, light housework, office work).	80	Normal activity with effort; some signs or symptoms of disease.
		70	Cares for self, unable to carry on normal activity or to do active work.
2	In bed <50% of the time. Ambulatory and capable of all self-care, but unable to carry out any work activities. Up and about more than 50% of waking hours.	60	Requires occasional assistance, but is able to care for most of his/her needs.
		50	Requires considerable assistance and frequent medical care.
3	In bed >50% of the time. Capable of only limited self-care, confined to bed or chair more than 50% of waking hours.	40	Disabled, requires special care and assistance.
		30	Severely disabled, hospitalization indicated. Death not imminent.
4	100% bedridden. Completely disabled. Cannot carry on any self-care. Totally confined to bed or chair.	20	Very sick, hospitalization indicated. Death not imminent.
		10	Moribund, fatal processes progressing rapidly.
5	Dead.	0	Dead.

APPENDIX B TUMOR ASSESSMENT PER IMMUNE-RELATED RESPONSE CRITERIA

Immune-related Response Criteria (irRC) are derived from modified World Health Organization (mWHO) conventions. Assessments of lymph nodes are derived from current RECIST guidelines.

Definitions of Measurable/non-Measurable Lesions

All measurable and non-measurable lesions should be assessed at Screening and at the defined tumor assessment time points. Additional assessments may be performed, as clinically indicated for suspicion of progression.

Measurable Lesions

Measurable lesions are lesions that can be accurately measured in 2 perpendicular diameters, with at least 1 diameter ≥ 20 mm and the other dimension ≥ 10 mm (10 mm x 10 mm for spiral CT with cuts of 5 mm). The area will be defined as the product of the largest diameter with its perpendicular.

Lymph nodes may also be considered measurable. To be considered pathologically enlarged and measurable, a lymph node must be at least 15 mm in short axis when assessed by CT scan (CT scan slice thickness recommended to be no greater than 5 mm).

Non-Measurable Lesions

Non-measurable (evaluable) lesions are all other lesions, including unidimensional measurable disease and small lesions (lesions without at least 1 diameter ≥ 20 mm, or pathological lymph nodes with short axis □ 15 mm), and any of the following: lesions occurring in a previously irradiated area (unless they are documented as new lesions since the completion of radiation therapy), bone lesions, leptomeningeal disease, ascites, pleural or pericardial effusion, lymphangitis cutis/pulmonis, abdominal masses that are not pathologically/cytologically confirmed and followed by imaging techniques and cystic lesions. Lymph nodes that have a short axis < 10 mm are considered non-pathological and should not be recorded or followed.

Definitions of Index/non-Index Lesions

Index Lesions

Measurable lesions, up to a maximum of 5 lesions per organ and ten lesions in total, must be identified as index lesions to be measured at Screening. The index lesions should be representative of all involved organs. In addition, index lesions must be selected based on their size (eg, lesions with the longest diameters), their suitability for accurate repeat assessment by imaging techniques, and how representative they are of the subject's tumor burden. At Screening, a Sum of the Product Diameters (SPD) for all index lesions will be calculated and considered the baseline SPD. The baseline sum will be used as the reference point to determine the objective tumor response of the index lesions at tumor assessment.

Non Index Lesions

Measurable lesions, other than index lesions, and all sites of non-measurable disease, will be identified as non-index lesions. Non-index lesions will be evaluated at the same assessment time points as the index lesions. In subsequent assessments, changes in non-index lesions will contribute only in the assessment of complete response.

Calculation of Sum of Product of Diameters (SPD)
Sum of Product of Diameters is an estimate of tumor burden. The 2 greatest perpendicular diameters are used to estimate the size of each tumor lesion. The SPD is calculated as the sum of the product of the diameters for index tumor lesions. Several variations of the SPD are identified for the purpose of classification of tumor responses.

SPD at Baseline: The sum of the product of the diameters for all index lesions identified at baseline prior to treatment on Day 1.

SPD at tumor assessment: For every on-study tumor assessment collected per protocol or as clinically indicated, the SPD at tumor assessment will be calculated using tumor imaging scans. All index lesions and all new measurable lesions that have emerged after baseline will contribute to the SPD at tumor assessment (irSPD).

SPD at NADIR: For tumors that are assessed more than 1 time after baseline, the lowest value of the SPD (SPD Baseline or SPD at tumor assessment) is used to classify subsequent tumor assessments for each subject. The SPD at tumor assessment using the irRC for progressive disease incorporates the contribution of new measurable lesions. Each net percentage change in tumor burden per assessment using irRC accounts for the size and growth kinetics of both old and new lesions as they appear. In this study the irRC as defined by the Investigator will serve as the basis of key endpoints for efficacy analyses and guide clinical care.

Definition of Index Lesion Response
Immune-related Complete Response (irCR), which is defined as complete disappearance of all index lesions. Lymph nodes that shrink to < 10 mm short axis are considered normal.

Immune-related Partial Response (irPR), which is defined as a decrease, relative to baseline, of 50% or greater in the sum of the products of the 2 largest perpendicular diameters of all index and all new measurable lesions (ie, Percentage Change in Tumor Burden), in the absence of irCR.

Note: the appearance of new measurable lesions is factored into the overall tumor burden, but does not automatically qualify as progressive disease until the SPD increases by ≥ 25% when compared to SPD at nadir

Immune-related Stable Disease (irSD), which is defined as not meeting the criteria for irCR or irPR, in the absence of immune-related progressive disease (irPD)

Immune-related Progressive Disease (irPD), which is defined as at least a 25% increase in Tumor Burden (ie, taking sum of the products of all index lesions and any new measurable lesions) when compared to SPD at nadir.

Definition of Non-Index Lesion Response
Immune-related Complete Response (irCR), which is defined as complete disappearance of all non-index lesions. Lymph nodes that shrink to < 10 mm short axis are considered normal.

Immune-related Partial Response (irPR), non-index lesion(s) are not considered in the definition of PR, this term does not apply.

Immune-related Stable Disease (irSD), non-index lesion(s) are not considered in the definition of SD, this term does not apply.

Immune-related Progressive Disease (irPD), increases in number or size of non-index lesion(s) does not constitute progressive disease unless/until Tumor Burden increases by 25% (ie, the SPD at nadir of index lesions and any new measurable lesions increases by the required amount).

Impact of New Lesions on irRC
New lesions alone do not qualify as progressive disease. However their contribution to total tumor burden is included in the SPD which in turn feeds into the irRC for tumor response. Therefore, new non-measurable lesions will not discontinue any subject from the study.

Definition of Overall Response Using irRC Will Be Based on the Following Criteria:

- Immune-related Complete Response (irCR): Complete disappearance of all tumor lesions (index and non-index), together with no new measurable or unmeasurable lesions, for at least 4 weeks from the date of documentation of irCR. All lymph nodes short axes must be < 10 mm.
- Immune-related Partial Response (irPR): The sum of the products of the 2 largest perpendicular diameters of all index lesions is measured and captured as the SPD baseline. At each subsequent tumor assessment, the sum of the products of the 2 largest perpendicular diameters of all index lesions and of new measurable lesions are added together to provide the Immune Response Sum of the Product of the Diameters (irSPD). A decrease, relative to baseline of the irSPD of 50% or greater is considered an irPR, in the absence of irCR. Must be confirmed no less than 4 weeks from the first irPR.
- Immune-related Stable Disease (irSD): irSD is defined as the failure to meet criteria for immune complete response or immune partial response, in the absence of progressive disease.
- Immune-related Progressive Disease (irPD): It is recommended in difficult cases (eg, increase in SPD or irSPD accompanied with significant individual lesion regression, “mixed response”, or in presence of stable or improving performance status/clinical condition) to confirm PD at the following tumor assessment. Any of the following will constitute progressive disease:
    - At least 25% increase in the SPD of all index lesions over nadir SPD calculated for these lesions
    - At least a 25% increase in the SPD of all index lesions and new measurable lesions
    - (irSPD) over the nadir SPD calculated for the index lesions.
