# Supplementary material for: Phase II study of ipilimumab and nivolumab in leptomeningeal carcinomatosis
Source: Nat Commun. 2021 Oct 12;12:5954. doi: 10.1038/s41467-021-25859-y (PMC8511104; doi:10.1038/s41467-021-25859-y)
Supplement: Supplementary file 2 — Reporting Summary [file 41467_2021_25859_MOESM2_ESM.pdf]

nature research

Corresponding author(s): Priscilla Brastianos

Last updated by author(s): January 19, 2021

Reporting Summary

Nature Research wishes to improve the reproducibility of the work that we publish. This form provides structure for consistency and transparency in reporting. For further information on Nature Research policies, see our Editorial Policies and the Editorial Policy Checklist.

Statistics

For all statistical analyses, confirm that the following items are present in the figure legend, table legend, main text, or Methods section.

n/a	Confirmed	
☐	☒	The exact sample size (*n*) for each experimental group/condition, given as a discrete number and unit of measurement
☐	☒	A statement on whether measurements were taken from distinct samples or whether the same sample was measured repeatedly
☐	☒	The statistical test(s) used AND whether they are one- or two-sided<br>*Only common tests should be described solely by name; describe more complex techniques in the Methods section.*
☐	☒	A description of all covariates tested
☐	☒	A description of any assumptions or corrections, such as tests of normality and adjustment for multiple comparisons
☐	☒	A full description of the statistical parameters including central tendency (e.g. means) or other basic estimates (e.g. regression coefficient) AND variation (e.g. standard deviation) or associated estimates of uncertainty (e.g. confidence intervals)
☐	☒	For null hypothesis testing, the test statistic (e.g. *F*, *t*, *r*) with confidence intervals, effect sizes, degrees of freedom and *P* value noted<br>*Give P values as exact values whenever suitable.*
☒	☐	For Bayesian analysis, information on the choice of priors and Markov chain Monte Carlo settings
☒	☐	For hierarchical and complex designs, identification of the appropriate level for tests and full reporting of outcomes
☒	☐	Estimates of effect sizes (e.g. Cohen's *d*, Pearson's *r*), indicating how they were calculated

Our web collection on statistics for biologists contains articles on many of the points above.

Software and code

Policy information about availability of computer code

Data collection	Clinical data is collected with InForm Software (Version 6.2).
Data analysis	All statistical analyses were conducted using SAS 9.4 (SAS Institute Inc., Cary, NC, USA).

For manuscripts utilizing custom algorithms or software that are central to the research but not yet described in published literature, software must be made available to editors and reviewers. We strongly encourage code deposition in a community repository (e.g. GitHub). See the Nature Research guidelines for submitting code & software for further information.

Data

Policy information about availability of data

All manuscripts must include a data availability statement. This statement should provide the following information, where applicable:

- Accession codes, unique identifiers, or web links for publicly available datasets
- A list of figures that have associated raw data
- A description of any restrictions on data availability

The full study protocol is available as Supplementary Note 1 in the Supplementary Information file. Any requests for additional clinical data will be reviewed by the Dana-Farber/Harvard Cancer Center (DF/HCC) Institutional Review Board (IRB). Patient-related data not included in the paper were generated as part of a clinical trial and are subject to patient confidentiality. Any data and materials (e.g. tissue samples or imaging data) that can be shared will need approval from the DF/HCC IRB and a Material Transfer Agreement in place. All data shared will be deidentified. Any requests for clinical data should be addressed to Priscilla K. Brastianos (pbrastianos@mgh.harvard.edu).

Field-specific reporting

Please select the one below that is the best fit for your research. If you are not sure, read the appropriate sections before making your selection.

- [x] Life sciences
- [ ] Behavioural & social sciences
- [ ] Ecological, evolutionary & environmental sciences

For a reference copy of the document with all sections, see nature.com/documents/nr-reporting-summary-flat.pdf

Life sciences study design

All studies must disclose on these points even when the disclosure is negative.

Sample size	With a historical median overall survival of approximately 5-6 weeks in this patient population, (as determined from previous reports in the literature [please see references 1, 4-13 as noted in our manuscript] and our institutional database), a Simon two-stage design was used to compare a null hypothesis that OS3 would be 18% against an alternative of 44%. Nine patients were to be enrolled in the first stage. If 0 or 1 patients are alive at 3 months, the trial would stop early. If 2 or more patients are alive at 3 months, an additional 9 patients would be enrolled into the second stage until 18 patients were accrued thus satisfying our pre-specified requirement for sufficient statistical power in a cohort with a known limited survival. If at least 6 patients among the total of 18 patients are alive at 3 months, then the treatment would be considered promising in the cohort. This design had a type-I error of 8% (target 10%) and power of 86% (target 85%). If the null hypothesis were true, then the probability would be 0.50 of stopping at the end of the first stage of the Simon design. On October 23, 2018, a third patient was alive at three months; therefore, enrollment continued without pause into the second stage.
Data exclusions	No data was excluded from the analysis.
Replication	The pre-specified analytic cohort consisted of 18 patients who were enrolled. Enrolled patients were screened with pre-defined eligibility criteria, received dosing regimens based on primary tumor histology according to manufacturer guidelines, were followed at pre-specified intervals for imaging and clinical assessment, and imaging results were evaluated using standardized criteria (iRANO and RECIST 1.1). These pre-defined, standardized approaches were taken to mitigate variability in studying a heterogeneous patient cohort and ensure reproducibility of our findings in future clinical trials and/or routine clinical care. We have been careful to note in the manuscript that validation of our findings is needed in larger patient cohorts.
Randomization	This was a single arm, non-randomized phase II study as there is no standard treatment for patients with LMD. Enrolled patients met pre-specified criteria including age 18 years or older, histologically confirmed disease from any solid tumor with an ECOG PS < 2, normal organ and marrow function, life expectancy greater than 3 weeks, were on a stable dose of dexamethasone of 2 mg or less for 7 days prior to initiation of treatment, and had leptomeningeal meningitis as defined by positive cytology or biopsy, or had imaging findings consistent with leptomeningeal meningitis if cerebrospinal fluid cytology was negative. The pre-defined eligibility criteria were used to mitigate variability in an otherwise heterogeneous patient cohort.
Blinding	Blinding is not relevant to this study. This is a single arm Phase II study.

Reporting for specific materials, systems and methods

We require information from authors about some types of materials, experimental systems and methods used in many studies. Here, indicate whether each material, system or method listed is relevant to your study. If you are not sure if a list item applies to your research, read the appropriate section before selecting a response.

Materials & experimental systems

n/a	Involved in the study	
x		Antibodies
x		Eukaryotic cell lines
x		Palaeontology and archaeology
x		Animals and other organisms
	x	Human research participants
	x	Clinical data
x		Dual use research of concern

Methods

n/a	Involved in the study	
x		ChIP-seq
x		Flow cytometry
x		MRI-based neuroimaging

Human research participants

Policy information about studies involving human research participants

Population characteristics	Eighteen patients were enrolled at the Massachusetts General Hospital and the Dana-Farber Cancer Institute from February 2018 to April 2019. The majority of patients were female (61%) and white (77.8%) with a median age of 54 years (range 36-70; Table 1). Primary diagnosis of breast cancer occurred in 44% (N=8) of patients; 50% of these primary tumors were estrogen receptor (ER) positive, 25% were progesterone receptor (PR) positive, 25% were human epidermal growth factor receptor 2 (HER2) positive and 25% were triple negative. Primary diagnoses of anaplastic astrocytoma, esophageal adenocarcinoma, ependymoma, gastroesophageal junction adenocarcinoma, glioblastoma, non-small cell lung cancer (NSCLC), ovarian carcinoma and small cell lung cancer occurred, respectively, in one patient each. Two patients had a primary diagnosis of melanoma. No patients in the enrolled cohort had a known BRAF or EGFR mutation or an ALK rearrangement in their primary tumor. The median time between primary cancer diagnosis and study enrollment was 29.5 months (range: 3 to 276 months). At the time of primary tumor diagnosis, 22% (N=4) of patients had brain metastases, one patient had cerebrospinal fluid (CSF) involvement and 11% (N=2) had known extracranial disease. At the time of trial enrollment, LMD was confirmed in 72.2% of patients (N=13) by positive CSF cytology and 27.7% (N=5) by imaging), and 72.2% (N=13) of patients had two or more parenchymal brain metastases. Sixty-one percent (N=11) had known extracranial disease with most common sites being lung (N=6) and lymph nodes (N=3). Patients were heavily pre-treated having received an average of 3.1 prior systemic therapies (range: 1-8). No patients received concurrent radiation, surgery nor intrathecal therapy while on protocol. Thirty-three percent (N=6) of patients received prior radiation therapy for their primary tumor while fifty-percent (N=9) of patients received prior radiation for metastatic disease. Sixty-one percent (N=11) received CNS-directed radiation; 44% (N=8) completed their course prior to enrollment and 17% (N=3) completed their course after trial participation. Among patients who received CNS-directed radiation, 45% (N=5) received whole-brain radiation therapy (WBRT), 18% (N=2) received stereotactic radiosurgery, 18% (N=2) received intensity-modulated radiation therapy (IMRT), and 18% (N=2) received craniospinal irradiation. Seventy-two percent (N=13) received prior surgery for their primary tumor. Fifty percent (N=9) reported additional operations for metastatic disease (median number of operations of 2 (average 2.7, range: 1 to 8)). Twenty-eight percent (N=5) of patients received CNS-directed surgery for disease prior to trial enrollment; one of these patients received an additional operation after trial participation. Eighty-three percent (N=15) of patients had a ventriculoperitoneal shunt and/or ommaya reservoir placed but no concurrent intrathecal therapies were administered during trial enrollment. Thirty-nine percent (N=7) of patients reported additional malignancies which were not considered to be active or progressing including Hodgkin's lymphoma (N=1), melanoma (N=1), carcinoma of cervix (N=1), neuro-endocrine carcinoid tumor of the appendiceal orifice (N=1), esophageal carcinoma (N=1) and breast cancer (N=1). One patient had a compound nevus with mild melanocytic dysplasia (N=1). One patient had a stable lung nodule whose biopsy status was unknown. Seventy-eight percent (N=14) of patients received corticosteroids at any point while enrolled on trial. Of these 14 patients, 50% (N=7) were receiving corticosteroids at baseline during trial enrollment while the other 50% (N=7) initiated corticosteroids throughout the course of trial participation. Of those 7 patients already receiving corticosteroids at baseline, 5 later required increased dosing, one later tolerated decreased dosing and one of these patients required no corticosteroid dose change over the course of the trial.
Recruitment	Patients were recruited through our respective institutions predominantly via referral from the solid tumor disease programs and, to a lesser extent, referral from outside physicians. There certainly is an established referral pattern in our institutions whereby patients with breast and lung cancer are more likely to be referred to Neurooncology teams than patients with other solid tumor malignancies. However, the reason for this is that involvement of the central nervous system and, particularly, leptomeningeal metastases in these patients is more common. Further, the availability of standard of care pembrolizumab and other immune checkpoint inhibition as well as molecular targeted therapies is greater in patients with lung cancer and melanoma, which likely led to further selection of patients with breast cancer. Additionally, it is possible that geographic and socioeconomic factors that determine access to our institutions at the DF/HCC may have been a source of selection bias.
Ethics oversight	Dana-Farber/Harvard Cancer Center Institutional Review Board

Note that full information on the approval of the study protocol must also be provided in the manuscript.

Clinical data

Policy information about clinical studies

All manuscripts should comply with the ICMJEguidelines for publication of clinical research and a completedCONSORT checklist must be included with all submissions.

Clinical trial registration	Clinicaltrials.gov identifier NCT02939300
Study protocol	Submitted with the manuscript
Data collection	Eighteen patients were enrolled at the Massachusetts General Hospital and the Dana-Farber Cancer Institute from February 2018 to April 2019. Clinical data is collected with InForm Software (Version 6.2).
Outcomes	The primary end point was rate of overall survival at 3 months (OS3). Secondary objectives included toxicity, CNS response rate and progression free survival (PFS) for both parenchymal brain metastases and LMD using immune Response Assessment in Neurooncology (iRANO)21, and extracranial response rate and PFS using Response Evaluation Criteria in Solid Tumors (RECIST) 1.1. All radiographic images were reviewed centrally by radiologists through the Tumor Imaging Metrics Core (TIMC) using these prespecified imaging criteria.
